# Species-Specific Antimonial Sensitivity in *Leishmania* Is Driven by Post-Transcriptional Regulation of AQP1

**DOI:** 10.1371/journal.pntd.0003500

**Published:** 2015-02-25

**Authors:** Goutam Mandal, Srotoswati Mandal, Mansi Sharma, Karen Santos Charret, Barbara Papadopoulou, Hiranmoy Bhattacharjee, Rita Mukhopadhyay

**Affiliations:** 1 Department of Cellular Biology and Pharmacology, Florida International University, Herbert Wertheim College of Medicine, Florida, United States of America; 2 CHU de Quebec Research Center and Department of Microbiology-Infectious Disease and Immunology, University Laval, Quebec, Canada; McGill University, CANADA

## Abstract

*Leishmania* is a digenetic protozoan parasite causing leishmaniasis in humans. The different clinical forms of leishmaniasis are caused by more than twenty species of *Leishmania* that are transmitted by nearly thirty species of phlebotomine sand flies. Pentavalent antimonials (such as Pentostam or Glucantime) are the first line drugs for treating leishmaniasis. Recent studies suggest that pentavalent antimony (Sb(V)) acts as a pro-drug, which is converted to the more active trivalent form (Sb(III)). However, sensitivity to trivalent antimony varies among different *Leishmania* species. In general, *Leishmania* species causing cutaneous leishmaniasis (CL) are more sensitive to Sb(III) than the species responsible for visceral leishmaniasis (VL). *Leishmania* aquaglyceroporin (AQP1) facilitates the adventitious passage of antimonite down a concentration gradient. In this study, we show that *Leishmania* species causing CL accumulate more antimonite, and therefore exhibit higher sensitivity to antimonials, than the species responsible for VL. This species-specific differential sensitivity to antimonite is directly proportional to the expression levels of *AQP1* mRNA. We show that the stability of *AQP1* mRNA in different *Leishmania* species is regulated by their respective 3’-untranslated regions. The differential regulation of *AQP1* mRNA explains the distinct antimonial sensitivity of each species.

## Author Summary

The degree of response to antimonial drugs varies widely between species and even among strains of the same species of the protozoan parasite *Leishmania*. However, the molecular mechanism(s) is unknown. In this study, we show that *Leishmania* aquaglyceroporin AQP1 drives this species-specific antimonial resistance. Aquaglyceroporins are channel proteins that facilitate the passage of small uncharged molecules, such as glycerol and water, across the biological membranes. AQP1 helps the parasite cope with the osmotic challenges it faces during its life cycle. Additionally, AQP1 is an adventitious facilitator of antimonite, the active form of pentavalent antimonial drugs. We show that AQP1 expression level is species-specific, and less AQP1 in visceral species compared to the cutaneous species results in increased resistance to antimonials. We also demonstrate that the 3’-untranslated regions (3’-UTR) of the AQP1 mRNA is a major determining factor of species-specific regulation of AQP1. Along with water homeostasis, aquaglyceroporins are also involved in directed cell migration. The variable levels of AQP1 in different *Leishmania* species may enable them to find their appropriate niches in vertebrate hosts and cope with the species-specific osmotic challenges during their life cycles.

## Introduction

Leishmaniasis is a protozoan parasitic infection in humans and other mammals that is transmitted by the bites of sandflies. The infection is caused by more than 20 different *Leishmania* species. The clinical manifestations range from self-healing cutaneous leishmaniasis (CL) to a potentially life threatening mucocutaneous leishmaniasis (MCL) [1] to the lethal, if untreated, visceral lesihmaniasis (VL) [2]. The disease is endemic in parts of 88 countries in five continents—the majority of the affected countries are in the tropics and subtropics. Approximately two million new cases are estimated to occur annually, of which 1.5 million are categorized as CL and 500,000 as VL. The parasite exists in two distinct morphological forms. The promastigotes form resides in the insect gut and appears to have slipper-like bodies with long flagella. The vertebrate forms of the parasite, amastigotes, have spherical, oval-shaped, aflagelleted bodies that reside in the macrophages of mammalian hosts. The first line of treatment against all forms of leishmaniasis is the pentavalent antimony-containing drugs sodium stibogluconate (Pentostam) and meglumine antimonite (Glucantime). However, drug resistance is a major impediment to the treatment of leishmaniasis. For example, approximately 60% of the patients in India do not respond to antimonial treatment due to acquired resistance [3].

Mechanisms of antimonial resistance in *Leishmania* have been explored extensively for several decades and are considered to be multifactorial [4] [5]. We have shown that laboratory-raised arsenic resistant *L*. *tarentolae*, which are cross resistant to antimonials, overproduced trypanothione (T[SH]_2_) [6], the major reduced thiol in Kinetoplastida [7], and conferred resistance by providing excess Sb-[TS]_2_ conjugates for the efflux pump in the plasma membrane [8]. The Sb-[TS]_2_ conjugates were shown to be sequestered into small intracellular vesicles near the flagellar pocket [9]. These mechanisms also seemed to play an active role in the pathogenic *Leishmania* [4] and also in field isolates [10] [11]. Amastigote-specific pentavalent antimonial reducing capability has also been implicated in VL [12]. Variability in the frequency of incidence of clinical antimonial resistance among *Leishmania* species has been reported [13]. However, it is not known whether an intrinsic variation in antimonial sensitivity exists among different *Leishmania* species. We reported that *L*. *major* was 50–70 times more sensitive to antimonite when compared to *L*. *infantum* [14]. Sarkar et al (2012) reported that *Leishmania* strains causing self-healing CL exhibited greater susceptibility towards oxidative stress as a result of low thiol content [15]. However, a comprehensive species wide study of all antimonial resistance markers reported so far is absent, and hence, the mechanism(s) of species-specific antimonial sensitivity is unknown.

We discovered the first aquaglyceroporin from *Leishmania* (AQP1) and showed its direct relationship to antimonite [Sb(III)], the active component of Pentostam and Glucantime, uptake [14] and resistance [16]. Overexpression of AQP1 in *Leishmania* cells led to hypersensitivity to antimonite, and disruption of one of the two *LmAQP1* alleles in *L*. *major* conferred a 10-fold increase in resistance to Sb(III) [14]. Later, these findings were corroborated in field isolates from India [17,18] and Nepal [19]. Besides the metalloids arsenite [As(III)] and Sb(III), the water conduction capacity of AQP1 is 65% of that of the classical water channel, human AQP1. Unlike the *Trypanosoma* and *Plasmodium* AQPs, AQP1 is a mercurial independent water channel. It also conducts glycerol, glyceraldehyde, dihydroxyacetone and sugar alcohols. We have also identified AQP1’s key role in osmoregulation and osmotaxis, which play crucial functions during parasite transmission [20]. Also, AQP1 is the first aquaglyceroporin to be exclusively localized in the flagellum of any organism. In intracellular amastigotes, it is localized in the flagellar pocket, rudimentary flagellum, and contractile vacuoles [20]. We have shown the involvement of flexible loop C of AQP1 in determining the substrate specificity of the channel [21,22]. Additionally, we showed that AQP1 was positively regulated at the post-translational level by a mitogen activated protein kinase 2 [17]. Therefore, AQP1 plays a major role in *Leishmania* cellular physiology and drug response.

During the course of our research with AQP1, we noticed that the muccocutaneous and cutaneous species were much more sensitive to Sb(III) when compared to the visceral species. Since AQP1 is the sole facilitator of Sb(III) in *Leishmania*, we asked whether AQP1 is driving this species-specific antimony sensitivity. In the absence of RNA polymerase II promoters, *Leishmania* genes are constitutively transcribed from large gene clusters as polycistronic pre-mRNAs. Steady-state levels of mature monocistronic mRNAs are regulated post-transcriptionally primarily by trans-splicing and polyadenylation [23,24]. Several examples in *Leishmania* species support the notion that post-transcriptional regulation of developmentally expressed transcripts involves sequences present mainly in the 3’-UTR [25,26,27,28], and more rarely in intergenic regions between tandemly repeated genes [29,30]. The 3’-UTR also regulates logarithmic-stationary phase gene regulation [31].

In this study, we mapped and cloned the 3’-UTRs of *AQP1* mRNA from six different *Leishmania* species representative of different clinical pathologies and endemic regions. Each approximately 1.8-kb 3’-UTR is highly U-rich (30%) with only 49% GC (in a highly GC-rich [∼ 60%] genome), and contains several well-known instability elements described in higher eukaryotes. Although the AQP1 protein sequences among these six species are more than 80% homologous, 3’-UTRs of AQP1 mRNAs differ significantly. We show that the species-specific antimonial sensitivity in *Leishmania* is uniquely driven by AQP1, and that it is mediated by post-transcriptional regulation through the respective distinct 3’-UTR of each species-specific *AQP1* mRNA.

## Results

### Intrinsic difference in species-specific antimonial sensitivity in *Leishmania*

Work here was carried out on six *Leishmania* species: three cutaneous (*L*. *major*, *L*. *tropica* and *L*. *panamensis*), one mucocutaneous (*L*. *braziliensis*) and two visceral (*L*. *infantum* and *L*. *donovani*). Our rationale for choosing these species was to represent every endemic continent (Asia [*L*. *donovani*, *L*. *infantum*, *L*. *major* and *L*. *tropica*], Africa [same as Asia], Europe [*L*. *infantum*, *L*. *major and L*. *tropica*] and Americas [*L*. *infantum*, *L*. *braziliensis and L*. *panamensis*]); clinical manifestation (VL- *L*. *donovani*, *L*. *infantum*; CL- rest of the species; MCL- *L*. *braziliensis*); and mode of transmission (anthroponotic- *L*. *donovani* and *L*. *tropica*; zoonotic- rest of the species). Intrinsic difference in Sb(III) sensitivity in the representative six selected species was determined by exposing the promastigotes at increasing concentrations of potassium antimonyl tartrate. *L*. *infantum* was the least sensitive species. The EC_50_ data showed that it was 1.4 times more resistant when compared to *L*. *donovani* and 46, 15, 20 and 7 times more resistant than the cutaneous species, namely, *L*. *major*, *L*. *tropica*, *L*. *braziliensis* and *L*. *panamensis* respectively (Table 1). However, the cutaneous species also differed in sensitivity to Sb(III) among themselves. *L*. *tropica*, *L*. *braziliensis* and *L*. *panamensis* were 3, 2.3 and 6 times more resistant to Sb(III), respectively, when compared to *L*. *major* (Table 1).

**Table 1:** Antimonial sensitivity of different *Leishmania* species.

Species	EC_50_	
	[Sb(III)] μM	[Sb(V)] μM
*L*. *braziliensis*	10 ± 2	55 ± 7
*L*. *donovani*	144 ± 5	800 ± 23
*L*. *infantum*	199 ± 22	1,100 ± 42
*L*. *major*	4.3 ± 0.3	44 ± 6
*L*. *panamensis*	27 ± 4	128 ± 12
*L*. *tropica*	13 ± 1	115 ± 9

EC_50_ values for Sb(III) for each cell type were determined for the promastigote form of the parasite. EC_50_ values for Sb(V) were determined for intracellular amastigotes.

To study whether this species-specific antimony sensitivity was also operational in amastigotes, we determined the EC_50_ of potassium hexahydroxy antimonate [Sb(V)] using intra-macrophagial amastigote model. We did not use pentavalent organo-antimonials as contaminating Sb(III) levels can represent more than 30% of total Sb [32]. The EC_50_ data showed that *L*. *infantum* was the least sensitive species among the six we tested. It was 1.4, 25, 9.6, 20, and 8.6 times more resistant when compared to *L*. *donovani*, *L*. *major*, *L*. *tropica*, *L*. *braziliensis*, *and L*. *panamensis*, respectively (Table 1). Again there was a slight variation in sensitivity among the cutaneous species. *L*. *tropica*, *L*. *braziliensis* and *L*. *panamensis* were 2.6, 1.3 and 3 times more resistant to SbV when compared to *L*. *major* (Table 1). In general, the CL species were much more sensitive to antimony compared to the VL species.

### CL species accumulate more Sb(III) than the VL species

**Fig 1:**
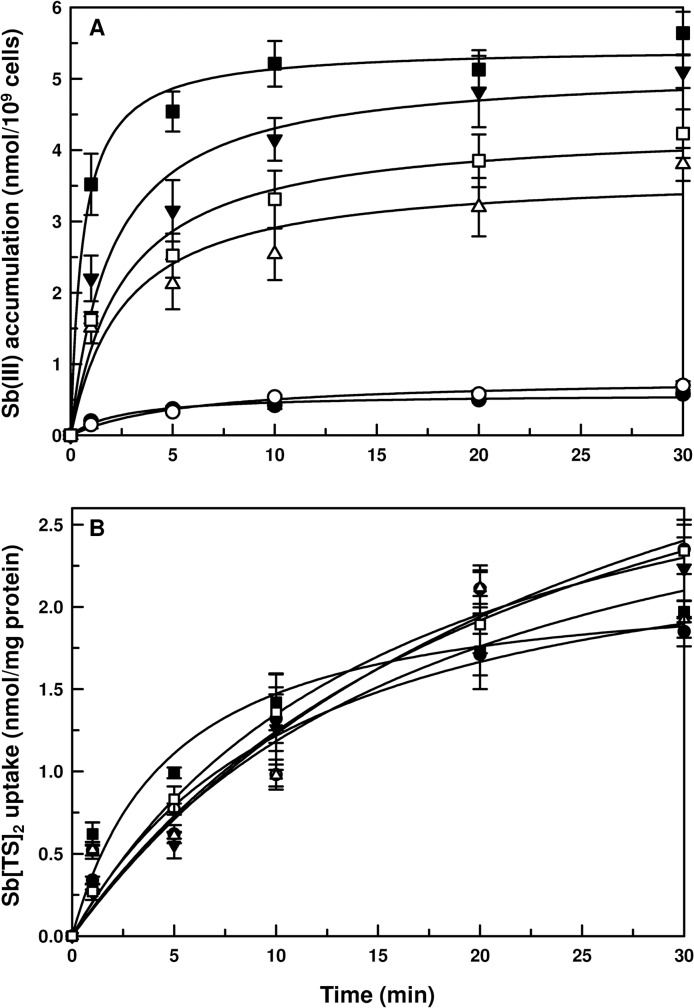
Sb(III) uptake and efflux by different species of *Leishmania*. **A**. Promastigotes of different species of *Leishmania* were exposed to 10 μM potassium antimony tartrate [Sb(III)]. Cells were harvested at different time points and antimony accumulation was estimated using ICP-MS. **B**. Everted plasma membrane enriched vesicles were prepared from promastiogtes of different species and transport assayed with 0.1 mM Sb(TS)_2_ and 10 mM ATP as energy source. The values at each time point were corrected for non-specific binding by subtraction of the values obtained with 10 mM AMP. Data were expressed as mean ± SE of three independent experiments in triplicate.-●- *L*. *donovani*,-○- *L*. *infantum*,-▼- *L*. *major*,-△- *L*. *tropica*,-■- *L*. *braziliensis*,-□- *L*. *panamensis*.

To understand this differential intrinsic antimonial sensitivity among the species in greater detail, we first examined the time-dependent intracellular accumulation of Sb(III) in the promastigotes. *L*. *braziliensis* showed the fastest and highest accumulation of Sb(III) at any given time followed by *L*. *major*, *L*. *panamensis* and *L*. *tropica* (Fig. 1A). The lowest rate and total accumulation of Sb(III) were observed in *L*. *donovani* and *L*. *infantum* (Fig. 1A). Generally, the antimony sensitive CL species accumulated more total antimony when compared to the VL species. Did this signify lower uptake or increased efflux in the VL species and higher uptake or slower efflux in the CL species? To answer this question, we prepared everted membrane vesicles from promastigotes of each species, and Sb(TS)_2_ conjugate accumulation was measured in the presence of 10 mM ATP as an energy source. The rates of transport of Sb-[TS]_2_ conjugate in the everted membrane vesicles of the six species were not significantly different from each other (Fig. 1B). These results indicate that differential sensitivity among different species is not due to a change in the efflux rate of antimony.

### Factors involved in species-specific antimonial sensitivity

**Fig 2:**
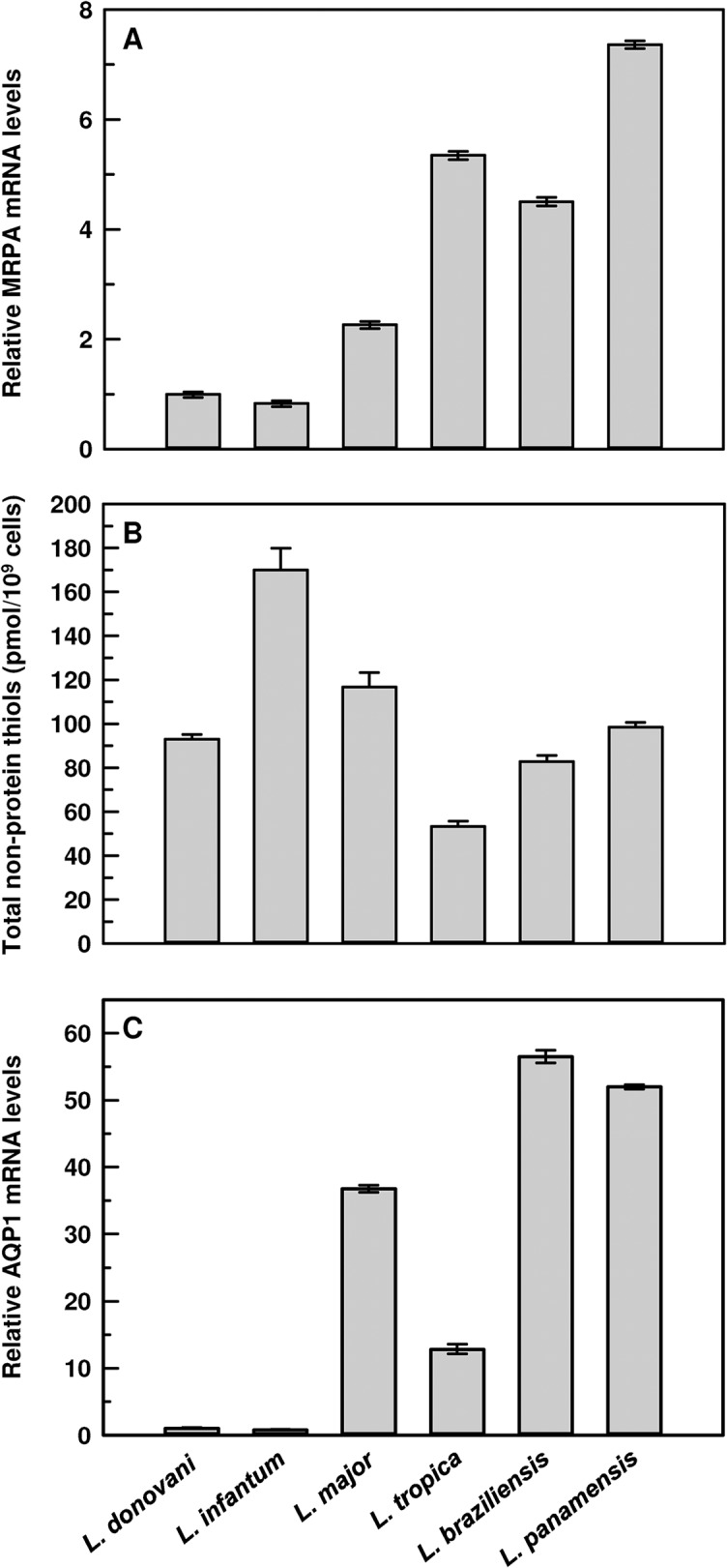
Common factors of antimonial resistance in different species of *Leishmania*. **A**. Levels of *MRPA* mRNA: Total RNA was isolated from promastigotes of different species and *MRPA* mRNA expression levels were estimated using qPCR. Relative (with respect to *L*. *donovani*) MRPA expression levels were calculated using 2^-ΔΔCt^ method. Data were expressed as mean ± SD of three independent experiments in triplicate. **B**. Levels of total non-protein thiols: Promastigotes from different species of *Leishmania* were harvested and proteins were precipitated using tricloroacetic acid. Total non-protein thiols were estimated using dithionitrobenzoic acid. Data were expressed as mean ± SE of three independent experiments in triplicate. **C**. Levels of *AQP1* mRNA: Total RNA was isolated from promastigotes of different species and *AQP1* mRNA expression levels were estimated using qPCR. Relative (with respect to *L*. *donovani*) AQP1 expression levels were calculated using 2^-ΔΔCt^ method. Data were expressed as mean ± SD of three independent experiments in triplicate.

Mechanisms of antimonial resistance in *Leishmania* have been proposed to be multifactorial. Four major components shown to be modulated in many laboratory-raised and clinical antimonial resistant *Leishmania* spp. are: (i) higher MRPA level for greater intracellular sequestration of Sb-[TS]_2_ conjugates; (ii) overproduction of total thiols, specifically T[SH]_2_; (iii) faster efflux of Sb-[TS]_2_ conjugates through an unknown efflux system in the plasma membrane; and (iv) downregulation of AQP1. Thus, we examined the *MRPA* mRNA levels in all six species first. The CL species showed much more *MRPA* mRNA when compared to the VL species. *L*. *panamensis* had the highest level of *MRPA* mRNA, which was about 7.4 fold more compared to *L*. *donovani* (Fig. 2A). *L*. *donovani* and *L*. *infantum* had similar levels of *MRPA* mRNA. The levels of *MRPA* mRNA in *L*. *major*, *L*. *tropica*, *L*. *braziliensis* and *L*. *panamensis* were approximately 2.3, 5.4, 4.5 and 7.4 fold respectively, compared to *L*. *donovani* (Fig. 2A). Therefore, having more *MRPA* mRNA does not justify the CL species being more sensitive to the antimonials. More mRNA does not necessarily result in more protein. However, the difference at the MRPA protein level between the CL and VL species does not explain the difference in antimony sensitivity between the species.

Next, we measured the total non-protein thiol levels among all the species. There was no correlation between total thiol levels and species-specific antimonial sensitivity (Fig. 2B). For example, three CL species, *L*. *major*, *L*. *braziliensis*, and *L*. *panamensis*, and one VL species, *L*. *donovani*, showed similar levels of non-protein total thiols (Fig. 2B). Another CL species, *L*. *tropica*, showed almost one-half of the thiol levels compared to other CL species, whereas *L*. *infantum* had approximately double that of *L*. *donovani*. Fig. 1B shows that the activity of the efflux pump was similar in all six species. Taken together, these data indicated that the levels of total Sb(III) accumulation can only be different if the rate of uptake differed among the species.

Next, we measured the mRNA levels of AQP1—the only Sb(III) facilitator in *Leishmania*. Relative (normalized against *L*. *donovani*) *AQP1* mRNA levels were calculated by the 2^-ΔΔCt^ method. The *AQP1* mRNA levels corroborated with our EC_50_ data in promastigotes and intracellular amastigotes. *L*. *infantum* showed the lowest level of *AQP1* mRNA among the species tested. *L*. *major*, *L*. *tropica*, *L*. *braziliensis* and *L*. *panamensis* produced 37, 13, 56 and 52 fold more *AQP1* mRNA, respectively, when compared to *L*. *donovani* (Fig. 2C). Therefore, the CL species showed much more *AQP1* mRNA accumulation than the VL species, which also corroborated our Sb(III) accumulation data (Fig. 1A).

### Osmoregulatory capacity of promastigotes is species-specific

**Fig 3:**
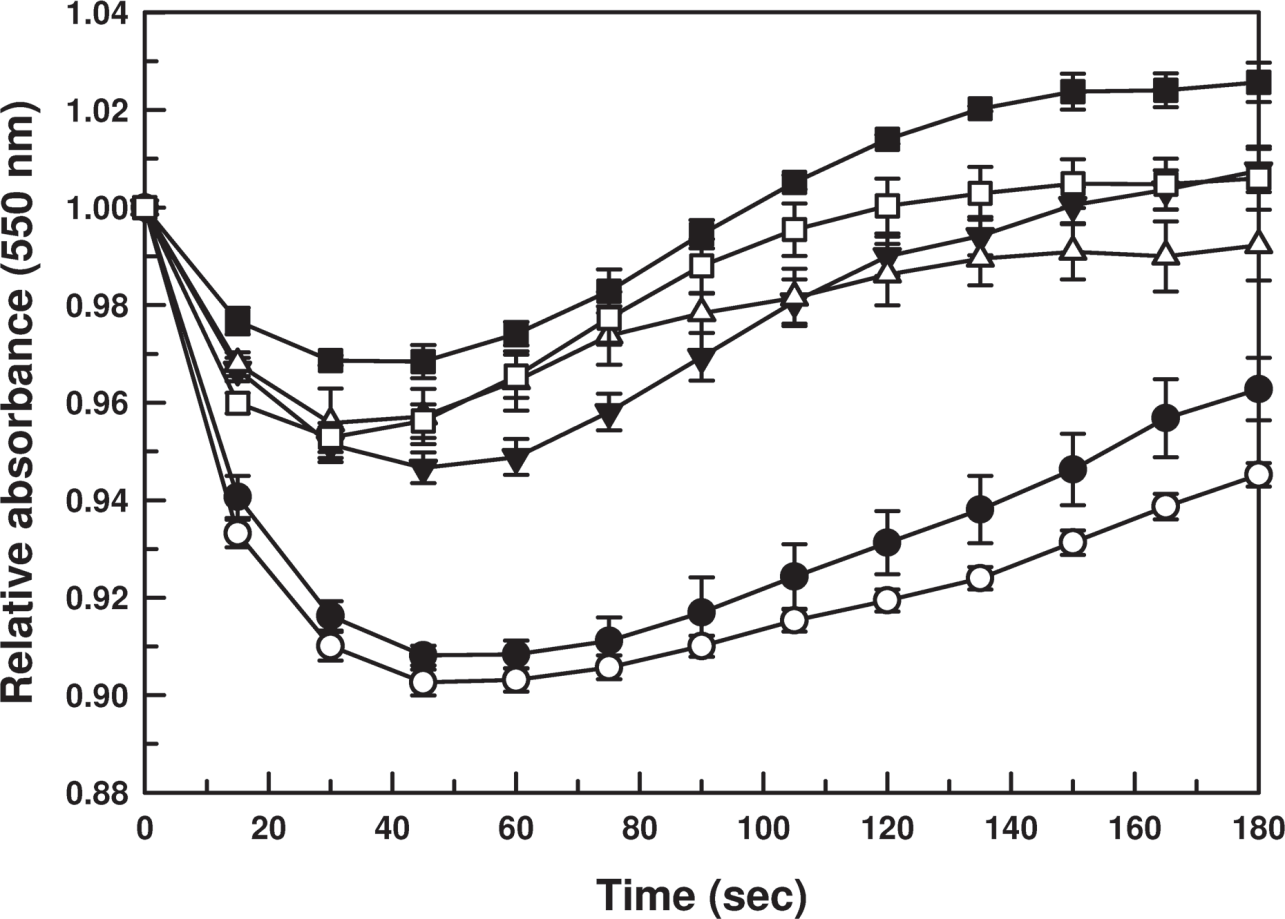
Volume regulation of promastigotes of different species of *Leishmania*. Promastigotes of different species of *Leishmania* were subjected to a hypo-osmotic shock, and the relative changes in cell volume were measured by monitoring the absorbance at 550 nm. Data were expressed as mean ± SE of three independent experiments in triplicate.-●- *L*. *donovani*,-○- *L*. *infantum*,-▼- *L*. *major*,-△- *L*. *tropica*,-■- *L*. *braziliensis*,-□- *L*. *panamensis*.

We have shown that the physiological function of AQP1 in *Leishmania* is osmoregulation. The strategic flagellar localization of AQP1 helps the parasite sense the changing osmotic environments between the vector and the host [20]. We could not detect native AQP1 expression at the protein levels by our anti-peptide polyclonal anti-AQP1 antibody in wild type *Leishmania* promastigotes [20]. Thus, it was reasonable to determine the functionality of AQP1 in all six species by determining their osmoregulatory capacities under hypo-osmotic shock (50% reduction in extracellular osmolarity), as we previously showed that osmoregulatory capacity was directly proportional to the AQP1 protein levels [20] [17] in the membrane. We observed that the CL species osmoregulate more efficiently when compared to the VL species, suggesting that CL species express more functional AQP1 than the VL species (Fig. 3). This corroborated with their EC_50_ (Table 1), Sb(III) accumulation (Fig. 1A) and *AQP1* mRNA levels (Fig. 2C), i.e., promastigotes with less *AQP1* mRNA (VL species) accumulated less amount of Sb(III), which resulted in higher resistance to antimonials when compared to promastigotes with more *AQP1* mRNA (CL species). *L*. *donovani* and *L*. *infantum* promastigotes swelled more rapidly (drops in absorbance) and recovered their volumes (rises in absorbance) slowly when compared to all CL species (Fig. 3). It is interesting to note that the CL *species L*. *braziliensis* and *L*. *panamensis* showed the highest levels of *AQP1* mRNA (Fig. 2C), and they were the most efficient osmoregulators (Fig. 3). Among the VL species, *L*. *infantum* showed the highest antimonial resistance and was the poorest osmoregulator. It swelled more than *L*. *donovani* and recovered even slower from the hypo-osmotic shock (Fig. 3). There is a very strong correlation between the antimony sensitivity among the species, the osmoregulatory capacity and AQP1 mRNA levels of the promastigotes.

### *AQP1* mRNA is more stable in the CL species

**Fig 4:**
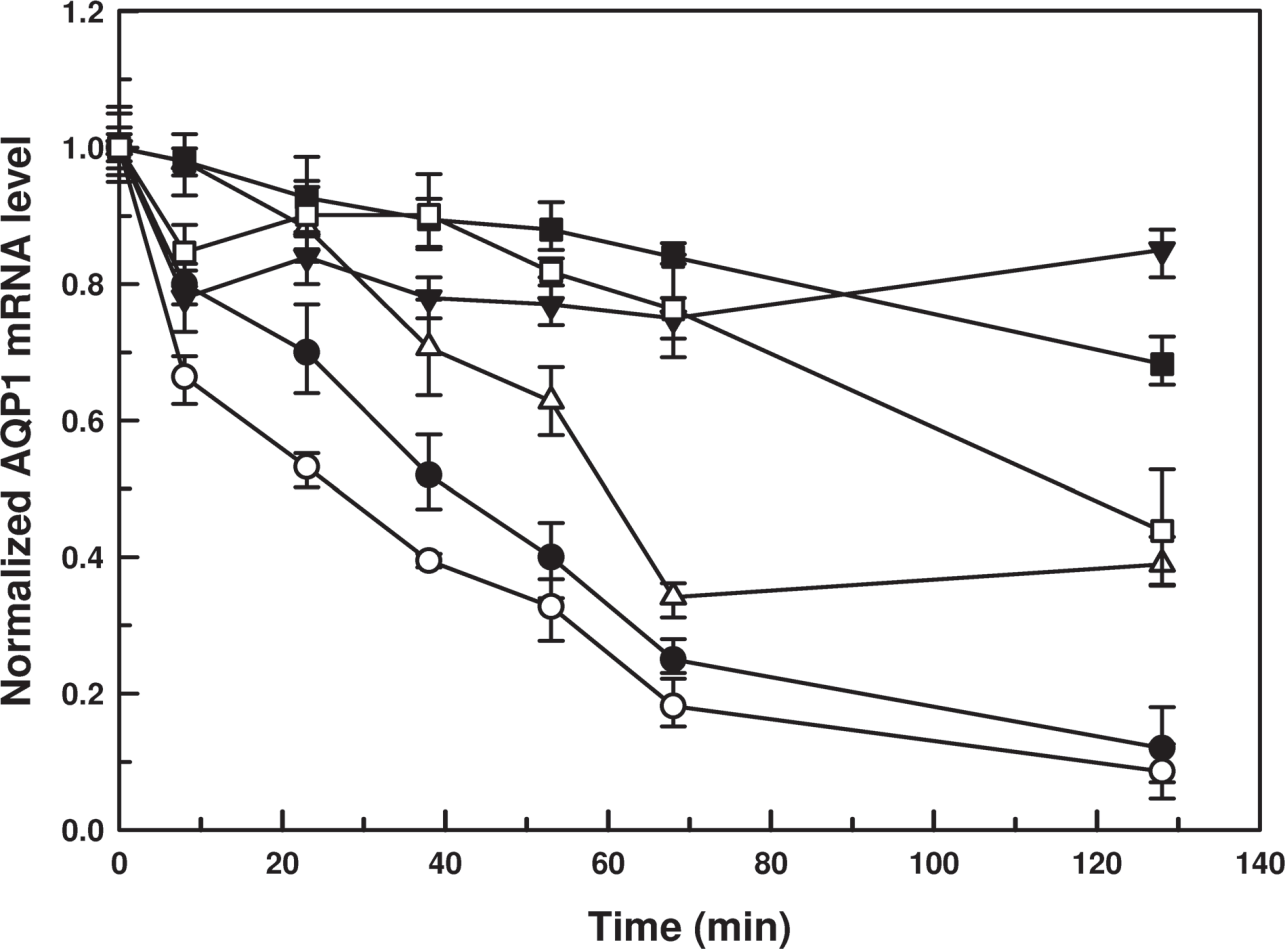
Stability of *AQP1* mRNA in promastigotes of different species of *Leishmania*. Promastigotes of different species of *Leishmania* were exposed to sinefungin followed by actinomycin D treatment. Cells were harvested just before the exposure of actinomycin D (0 minute) and at different time points after the exposure to actinomycin D. Total RNA was isolated and *AQP1* mRNA levels were estimated using qPCR. Relative (with respect to 0 minute) *AQP1* mRNA levels were calculated using 2^-ΔΔCt^ method. Data were expressed as mean ± SD of three independent experiments in triplicate.-●- *L*. *donovani*,-○- *L*. *infantum*,-△- *L*. *major*,-▼- *L*. *tropica*,-■- *L*. *braziliensis*,-□- *L*. *panamensis*.

Since *Leishmania* does not have any transcriptional control, we investigated the stability of *AQP1* mRNA in all six species to determine if any post-transcriptional regulation is active in lowering the mRNA levels in the VL species. To determine the turnover rate of *AQP1* mRNA, the mid-log phase promastigotes of all species were treated with sinefungin (to stop pre-mRNA processing) [33] followed by actinomycin D (to inhibit transcription), [34] and cells were harvested up to 130 minutes. The decay of *AQP1* mRNA was determined by qPCR and normalized against the 0 minute (time of addition of actinomycin D). The half-lives of *AQP1* mRNA from VL species, i.e., *L donovani* and *L*. *infantum*, were determined to be 40 min and 26 min, respectively; on the other hand, the half-lives of the *AQP1* mRNA from CL species, such as *L*. *tropica* and *L*. *panamensis*, were 57 and 117 min, respectively. The half-lives of the *AQP1* mRNA in *L*. *braziliensis* and *L*. *major* were estimated to be > 130 min (Fig. 4). Therefore, *AQP1* mRNA from the CL species was much more stable than from the VL species, which follows a similar trend that was observed in their respective steady-state levels of *AQP1* mRNA: *L*. *braziliensis* ≥ *L*. *panamensis* > *L*.*major* > *L*. *tropica* > *L*. *donovani* > *L*. *infantum* (Fig. 2C).

### Cloning of the 3’-UTRs of *AQP1* mRNA from different species

Since mRNA stability in *Leishmania* is mostly controlled by the 3’-UTR [23], we cloned the individual 3’-UTRs of *AQP1* mRNA from all six species. The length of the 3’-UTR of the *AQP1* mRNA was approximately 1.8 kb in all species examined as mapped by 3’-RACE PCR. The protein sequence alignment of AQP1 from all six species showed that they were very close to each other, with an overall similarity of about 88% (S1 Fig.). Additionally, the overall identity between the open reading frames (ORF) of *AQP1* in all six species was about 66% (S2 Fig.). However, 3’-UTRs from all six species were more divergent and showed an overall identity of about 24% (S3 Fig.). A greater similarity was seen between the pairs. *L*. *infantum* and *L*. *donovani* 3’-UTRs were 99% identical (S4 Fig.). The CL species 3’-UTRs can be divided into two groups. *L*. *tropica* and *L*. *major* were 78% identical (S5 Fig.), while *L*. *panamensis* and *L*. *braziliensis* 3’-UTRs were 94% identical (S6 Fig.). Thus, based on their 3’-UTR sequences, the six species we examined can be divided into three distinct groups: VL (*L*. *donovani* and *L*. *infantum*), CL1 (*L*. *major* and *L*. *tropica*) and CL2 (*L*. *braziliensis* and *L*. *panamensis*) groups.

### Role of the 3’-UTR in species-specific stability of AQP1 mRNA

**Fig 5:**
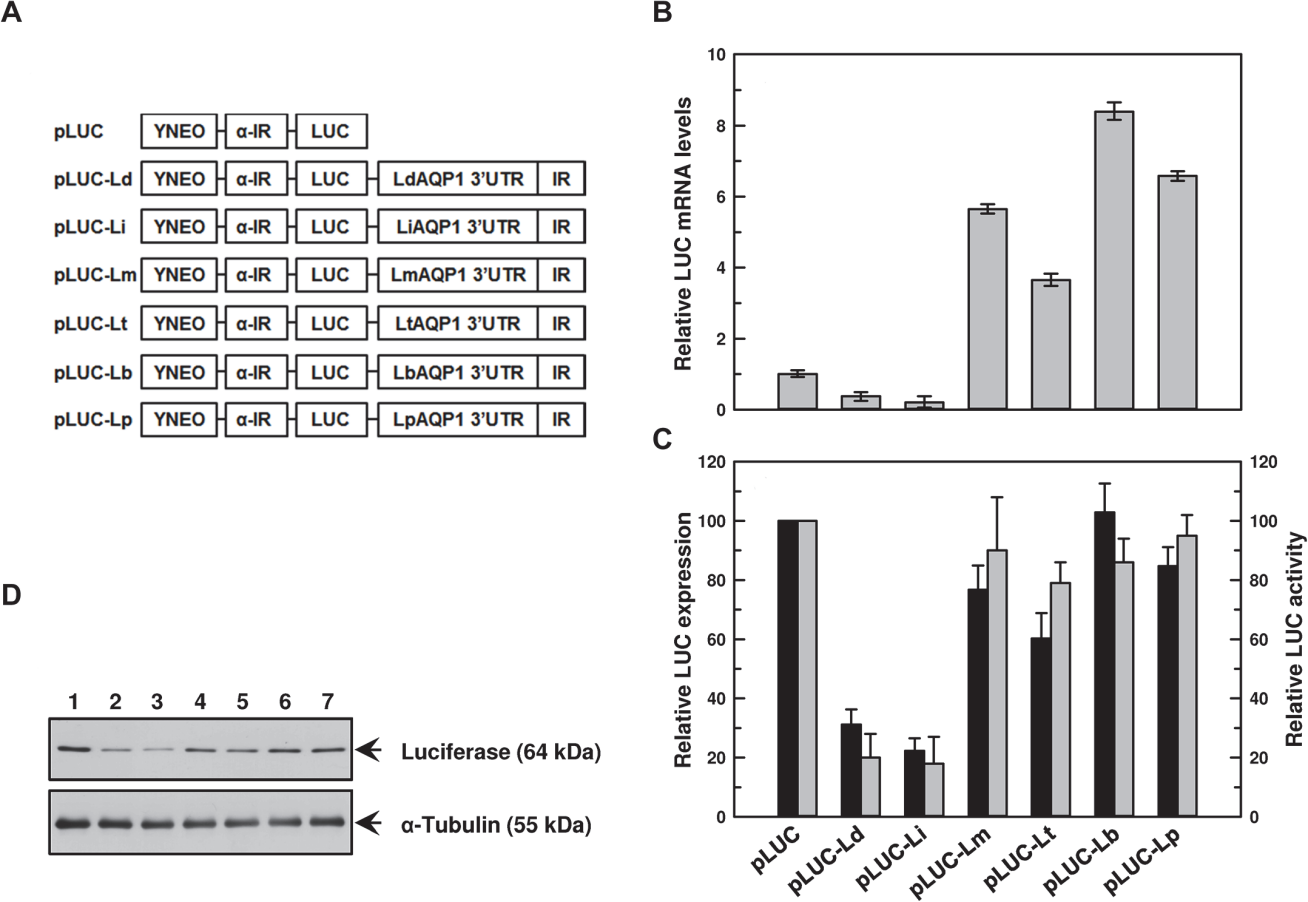
Effect of *AQP1* 3’-UTR from different species of *Leishmania* on levels of *LUC* mRNA, protein expression and activity when transfected in promastigotes of *L*. *donovani*. **A**. Chimeric constructs with the luciferase (LUC) reporter gene placed under the control of the 3’-UTR of the AQP1 mRNA from different *Leishmania* species were prepared to assess the role of these sequences in the species-specific regulation process. The neomycin resistance gene (NEO) and *LUC* transcripts in pSPYNEOαLUC vector were processed either by a 92 synthetic polypyrimidine stretch (Y90AG) (YNEO) or by the intergenic region of the α-tubulin gene (α-IR), respectively. The 3’-UTR (approximately 1.8 kb) followed by a 200 bp intergenic sequence of the *AQP1* 3’-UTR (IR) from different species were PCR amplified from the genome of different species of *Leishmania* and introduced at the 3’ end of the *LUC* gene. The LUC-expressing constructs were transfected into different species of *Leishmania*, and the effects of the AQP1 3’-UTR on *LUC* mRNA and protein expression and activity were measured. **B**. *LUC* mRNA levels: Total RNA was isolated from promastigotes of *L*. *donovani* expressing different chimeric constructs of *LUC*, and *LUC* mRNA expression levels were estimated using qPCR. Relative (with respect to *LUC*) *LUC* mRNA expression levels were calculated using 2^-ΔΔCt^ method. Data were expressed as mean ± SD of three independent experiments in triplicate. **C**. LUC activity and expression: Estimation of LUC activity (□) was carried out using whole cell lysates. Percent LUC activity was calculated keeping vector control at 100%. Data were expressed as mean ± SE of three independent experiments in triplicate. **D**. Representative Western blot analysis of transfected promastigotes: Whole cell (1 x 10^6^/lane) lysates of different transfectants were fractionated on SDS-PAGE and blotted onto nitrocellulose membrane. Levels of LUC expressions were detected using an anti-luciferase antibody. α-tubuline was used as loading control. Lanes: 1. pLUC, 2. pLUC-Ld, 3. pLUC-Li, 4. pLUC-Lm, 5. pLUC-Lt, 6. pLUC-Lb, and 7. pLUC-Lp. Amount of luciferase expression (■) relative to cells transfected with pSPYNEOαLUC was estimated by densitometric analysis using ImageJ software followed by normalization against the amount of α-tubulin of the respective cells. Error bars were calculated from the mean ± SE of two independent experiments. *Ld- L*. *donovani*, *Li- L*. *infantum*, *Lm- L*. *major*, *Lt- L*. *tropica*, *Lb- L*. *braziliensis*, *Lp- L*. *panamensis*.

Based on the data presented above, we concluded that the stability of *AQP1* mRNA plays a significant role in dictating the species-specific antimonial sensitivity in *Leishmania*. Given that 3’-UTR sequences between VL and CL species are very divergent, we hypothesized that these sequences are responsible for the species-specific differential *AQP1* mRNA stability. To determine the underlying mechanism, a series of chimeric constructs were made with the full length 3’-UTR (∼ 1.8 kb) of the *AQP1* mRNA from each of the six species by cloning them at the 3’ end of the luciferase (*LUC*) reporter gene (Fig. 5A). To direct accurate 5’ and 3’ processing of the *LUC* chimeric transcripts, these cassettes were flanked by an upstream α-tubulin intergenic (IR) region and by a downstream IR region (∼ 200 bp) of each species-specific 3’-UTR of *AQP1* mRNA. In trypanosomatids, polyadenylation is often directed by trans-splicing signals that are located 100–400 nucleotides downstream of the polyadenylation site [28,35,36]. These chimeras were expressed from an episomal plasmid pSPYNEOαLUC with neomycin phosphotransferase (NEO) as a marker. The vector alone, where LUC expression was only regulated by the intergenic regions of α-tubulin, is referred to in the present work as pLUC. Each of the species-specific *LUC*-*AQP1-*3’-UTR constructs were named pLUC-Ld (*L*. *donovani*), pLUC-Li (*L*. *infantum*), pLUC-Lm (*L*. *major*), pLUC-Lt (*L*. *tropica*), pLUC-Lb (*L*. *braziliensis*), and pLUC-Lp (*L*. *panamensis*) (Fig. 5). Six individual chimeric constructs and the vector alone were transfected into the six species generating 42 transfectants. We also evaluated the relative copy number of *LUC*–containing plasmids by qPCR using pteridine reductase 1 (PTR1) as the housekeeping control, the levels of which are similar in all transfectants (S1A Table). The proper processing of 3’ end of UTRs from all transfectants with chimeric plasmids was determined by 3’-RACE PCR and sequencing. They had identical processing when compared to the 3’-UTR ends of the native *AQP1* mRNA. Therefore, it was reasonable to deduce that the LUC expression and activity in the transfectants would largely depend upon the steady-state levels of *LUC* mRNA dictated by their stability in each species. We thus tested the role of each of the six 3’-UTRs in regulating *LUC* mRNA steady-state levels, stability, LUC protein levels and LUC activity in a species-specific manner.

*L*. *donovani* transfected with pLUC-Li produced the lowest levels of *LUC* mRNA when compared to pLUC control, whereas pLUC-Ld produced slightly more (only 0.37 fold). However, accumulation of the *LUC* mRNA under the control of 3’-UTRs from the CL species was 3 to 8 fold higher than that of the pLUC control (Fig. 5B). pLUC-Lt, pLUC-Lm, pLUC-Lb, and pLUC-Lp transfectants produced 3.7, 5.7, 8.4 and 6.6 fold more *LUC* mRNA, respectively, compared to pLUC alone (Fig. 5B). These data corroborated with our *LUC* mRNA stability, LUC protein expression and activity, suggesting that regulation occurs at the level of *AQP1* mRNA stability. Indeed, *LUC* mRNA was most unstable in pLUC-Li transfectants, with a half-life of 25 min (Table 2). pLUC-Ld extended that for 39 min, and for pLUC-Lt, the half-life was 83 min. pLUC-Lm, pLUC-Lb, pLUC-Lp transfectants gave rise to the most stable *LUC* mRNA, extending their half-lives to over 130 min (Table 2). Western blot with an anti-luciferase antibody and densitometric analysis using α-tubulin as a loading control revealed that luciferase protein expression was lowest in pLUC-Li transfectants, at only about 21%, which resulted in about 18% luciferase activity when compared to pLUC control (Fig. 5C and D). pLUC-Ld, pLUC-Lm, pLUC-Lt, pLUC-Lb and pLUC-Lp transectants expressed 1.5, 4.5, 2.6, 5.5, and 4.7 times more luciferase, respectively, when compared to pLUC-Li. This is also comparable with LUC activity, which was increased in the following order in the transfectants: pLUC-Li < pLUC-Ld < pLUC-Lt < pLUC-Lb < pLUC-Lm < pLUC-Lp (Fig. 5C). Similar results were obtained when transfecting the chimeric *LUC* constructs with the 3’-UTRs in *L*. *infantum*. The 3’-UTRs from the CL species generated 3 to 5 fold more *LUC* mRNA when compared to the VL species (S7A Fig.). The CL 3’-UTRs also made *LUC* mRNA more stable (higher half-life) in *L*. *infantum* (Table 2). These results also corroborated with LUC expression and activity (S7B Fig.).

**Table 2:** Half-lives of *LUC* mRNA under the control of species-specific 3’ UTRs of AQP1 mRNA.

Type of leishmaniasis	Host species	LUC mRNA half-lives (minute)*						
		pLUC	pLUC-Ld	pLUC-Li	pLUC-Lm	pLUC-Lt	pLUC-Lb	pLUC-Lp
**VL**	***L*. *donovani***	>130	36 (32 to 44)	26 (23 to 29)	>130	74 (65 to 120)	>130	>130
	***L*. *infantum***	>130	56 (52 to 72)	54 (48 to 57)	>130	99 (88 to 111)	>130	>130
**CL**	***L*. *major***	>130	>130	>130	>130	>130	>130	>130
	***L*. *tropica***	>130	79 (72 to 120)	75 (70 to 110)	>130	>130	>130	>130
	***L*. *braziliensis***	>130	77 (72 to 103)	72 (66 to 100)	>130	>130	96 (80 to 106)	>130
	***L*. *panamensis***	>130	64 (62 to 91)	73 (68 to 104)	>130	117 (91 to >130)	95 (78 to 120)	>130

*The stability curves from where the half-lives were calculated are provided in the supplementary information (S12 Fig.)

**Fig 6:**
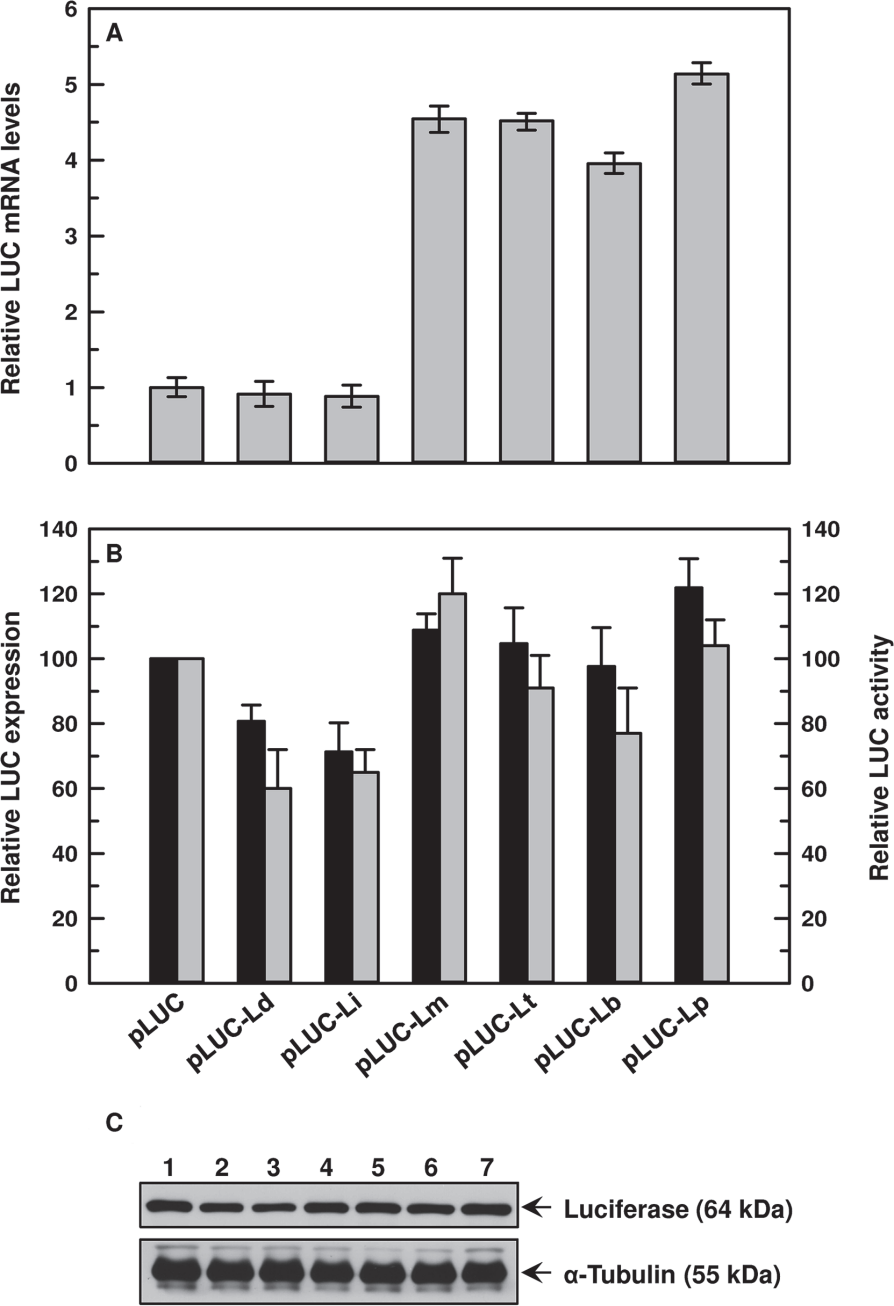
Effect of *AQP1* 3’-UTR from different species of *Leishmania* on levels of *LUC* mRNA, protein expression and activity when transfected in promastigotes of *L*. *major*. **A**. *LUC* mRNA levels: Total RNA was isolated from promastigotes of *L*. *major* expressing different chimeric constructs of *LUC*, and *LUC* mRNA expression levels were estimated using qPCR. Relative (with respect to *LUC*) *LUC* mRNA expression levels were calculated using 2^-ΔΔCt^ method. Data were expressed as mean ± SD of three independent experiments in triplicate. **B**. LUC activity and expression: Estimation of LUC activity (□) was carried out using whole cell lysates. Percent LUC activity was calculated keeping vector control at 100%. Data were expressed as mean ± SE of three independent experiments in triplicate. **C**. Representative Western blot analysis of transfected promastigotes: Whole cell (1 x 10^6^/lane) lysates of different transfectants were fractionated on SDS-PAGE and blotted onto nitrocellulose membrane. Levels of LUC expressions were detected using an anti-luciferase antibody. α-tubulin was used as loading control. Lanes: 1. pLUC, 2. pLUC-Ld, 3. pLUC-Li, 4. pLUC-Lm, 5. pLUC-Lt, 6. pLUC-Lb, and 7. pLUC-Lp. Amount of luciferase expression (■) relative to cells transfected with pSPYNEOαLUC was estimated by densitometric analysis using ImageJ software followed by normalization against the amount of α-tubulin of the respective cells. Error bars were calculated from the mean ± SE of two independent experiments. *Ld- L*. *donovani*, *Li- L*. *infantum*, *Lm- L*. *major*, *Lt- L*. *tropica*, *Lb- L*. *braziliensis*, *Lp- L*. *panamensis*.

When transfected in *L*. *major*, pLUC-Ld and pLUC-Li constructs produced similar basal levels of *LUC* mRNA when compared to pLUC alone. However, the 3’-UTRs from the CL species generated 4–5 fold more *LUC* mRNA (Fig. 6A). These data corroborated with our *LUC* mRNA stability, LUC protein expression and activity. *LUC* mRNA produced from all constructs in *L*. *major* were quite stable, and the half-life was determined to be >130 min (Table 2). Densitometric analysis revealed that LUC expression (Fig. 6C) is similar in all CL UTR constructs. pLUC-Li and pLUC-Ld transfectants express LUC at 77% and 93% of pLUC control (Fig. 6B), respectively. The highest LUC activity was observed in cells expressing LUC from pLUC-Lm, followed by pLUC-Lp, pLUC-Lt and pLUC-Lb. Similar results were obtained when transfecting the chimeric *LUC* constructs with the 3’-UTRs in *L*. *tropica*. *LUC* mRNA produced from all constructs in *L*. *tropica* were quite stable, and the half-life was determined to be >130 min, except in pLUC-Li and pLUC-Ld, which were 87 min and 89 min, respectively (S8A Fig., Table 2). Four CL 3’-UTRs regulated to produce 85–101% LUC protein compared to pLUC control, which resulted in 81–100% LUC activities in pLUC-Lm, pLUC-Lt, pLUC-Lb and pLUC-Lp transfectants (S8B Fig.).

**Fig 7:**
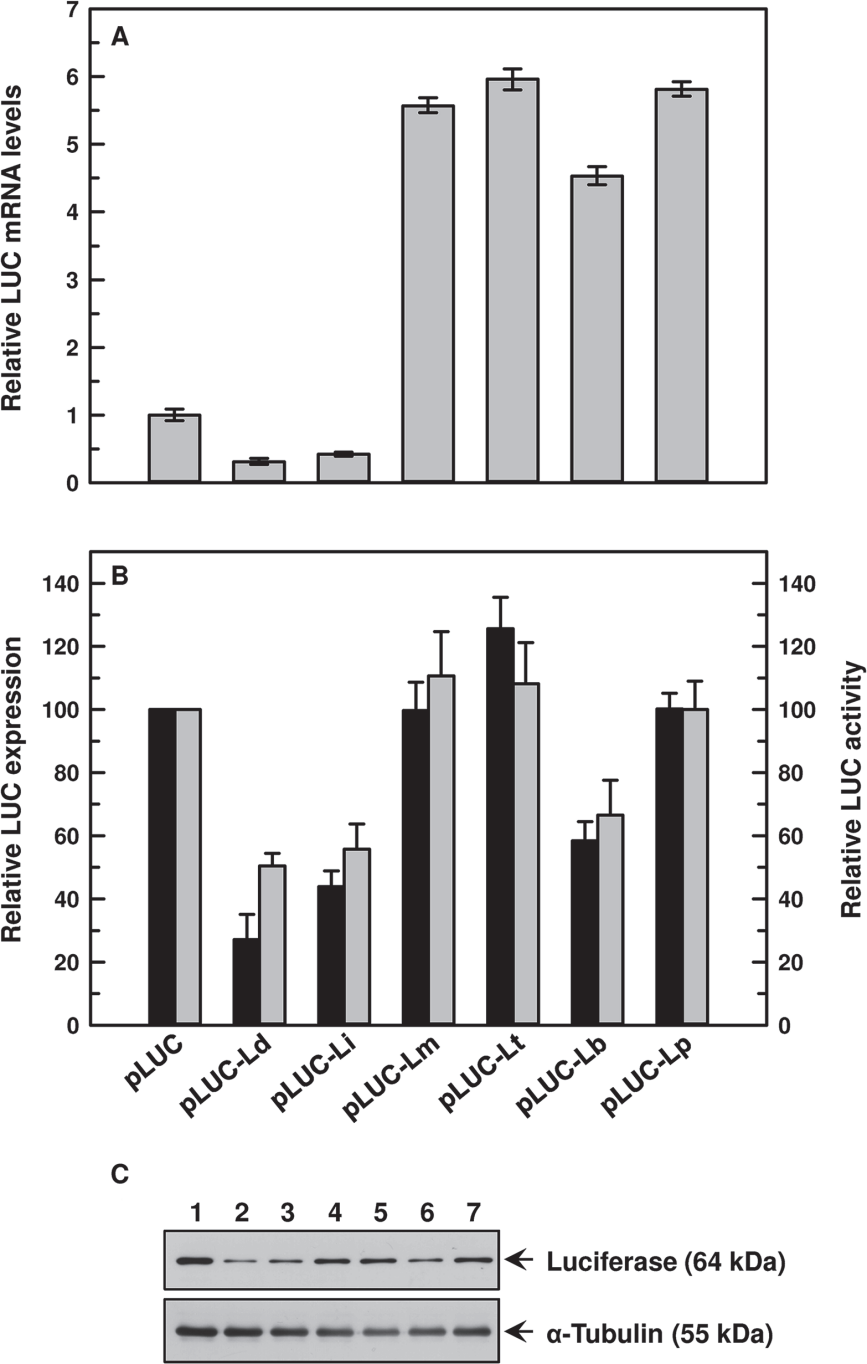
Effect of *AQP1* 3’-UTR from different species of *Leishmania* on levels of *LUC* mRNA, protein expression and activity when transfected in promastigotes of *L*. *braziliensis*. **A**. *LUC* mRNA levels: Total RNA was isolated from promastigotes of *L*. *braziliensis* expressing different chimeric constructs of *LUC* and *LUC* mRNA expression levels were estimated using qPCR. Relative (with respect to *LUC*) *LUC* mRNA expression levels were calculated using 2^-ΔΔCt^ method. Data were expressed as mean ± SD of three independent experiments in triplicate. **B**. LUC activity and expression: Estimation of LUC activity (□) was carried out using whole cell lysates. Percent LUC activity was calculated keeping vector control at 100%. Data were expressed as mean ± SE of three independent experiments in triplicate. **C**. Representative Western blot analysis of transfected promastigotes: Whole cell (1 x 10^6^/lane) lysates of different transfectants were fractionated on SDS-PAGE and blotted onto nitrocellulose membrane. Levels of LUC expressions were detected using an anti-luciferase antibody. α-tubulin was used as loading control. Lanes: 1. pLUC, 2. pLUC-Ld, 3. pLUC-Li, 4. pLUC-Lm, 5. pLUC-Lt, 6. pLUC-Lb, and 7. pLUC-Lp. Amount of luciferase expression (■) relative to cells transfected with pSPYNEOαLUC was estimated by densitometric analysis using ImageJ software followed by normalization against the amount of α-tubulin of the respective cells. Error bars were calculated from the mean ± SE of two independent experiments. *Ld- L*. *donovani*, *Li- L*. *infantum*, *Lm- L*. *major*, *Lt- L*. *tropica*, *Lb- L*. *braziliensis*, *Lp- L*. *panamensis*.

Promastigotes of *L*. *braziliensis* transfected with pLUC-Ld construct produced the lowest levels *LUC* mRNA when compared to pLUC alone, whereas pLUC-Li produced a little more (only 0.42 fold). However, the UTRs from the CL species generated 4.5–6.0 fold more *LUC* mRNA compared to pLUC cells (Fig. 7A). pLUC-Lb transfectants produced 4.5 fold more *LUC* mRNA, whereas pLUC-Lm, pLUC-Lt, and pLUC-Lp transfectants generated about 6 fold more (Fig. 7A) compared to pLUC. This data corroborated with our *LUC* mRNA stability, LUC protein expression and activity. *LUC* mRNAs produced from pLUC-Lm, pLUC-Lt and pLUC-Lp constructs in *L*. *braziliensis* were quite stable, and the half-lives were determined to be >130 min. The half-lives of *LUC* mRNAs generated from pLUC-Ld, pLUC-Li and pLUC-Lb were 84 min, 80 min and 98 min, respectively (Table 2). Densitometric analysis of LUC expression showed 72–75% of pLUC control in pLUC-Ld and pLUC-Li transfectants, which resulted in 60–65% of pLUC control LUC activity in those cells (Fig. 7B). The CL 3’-UTRs behaved in a similar manner, producing 101–122% luciferase protein compared to pLUC control, which resulted in 100–110% of pLUC control LUC activity in pLUC-Lm, pLUC-Lp and pLUC-Lt transfectants (Fig. 7B and C). However, LUC activity from pLUC-Lb construct was 86% compared to pLUC alone. Similar results were obtained in *L*. *panamensis* transfectants. The CL 3’-UTRs generated more stable *LUC* mRNAs with longer half-lives compared to the VL species (S9A Fig., Table 2). Higher LUC protein expression and activity were also observed in the CL species (S9B Fig.).

### Role of the 3’-UTR in species-specific functionality of AQP1

**Fig 8:**
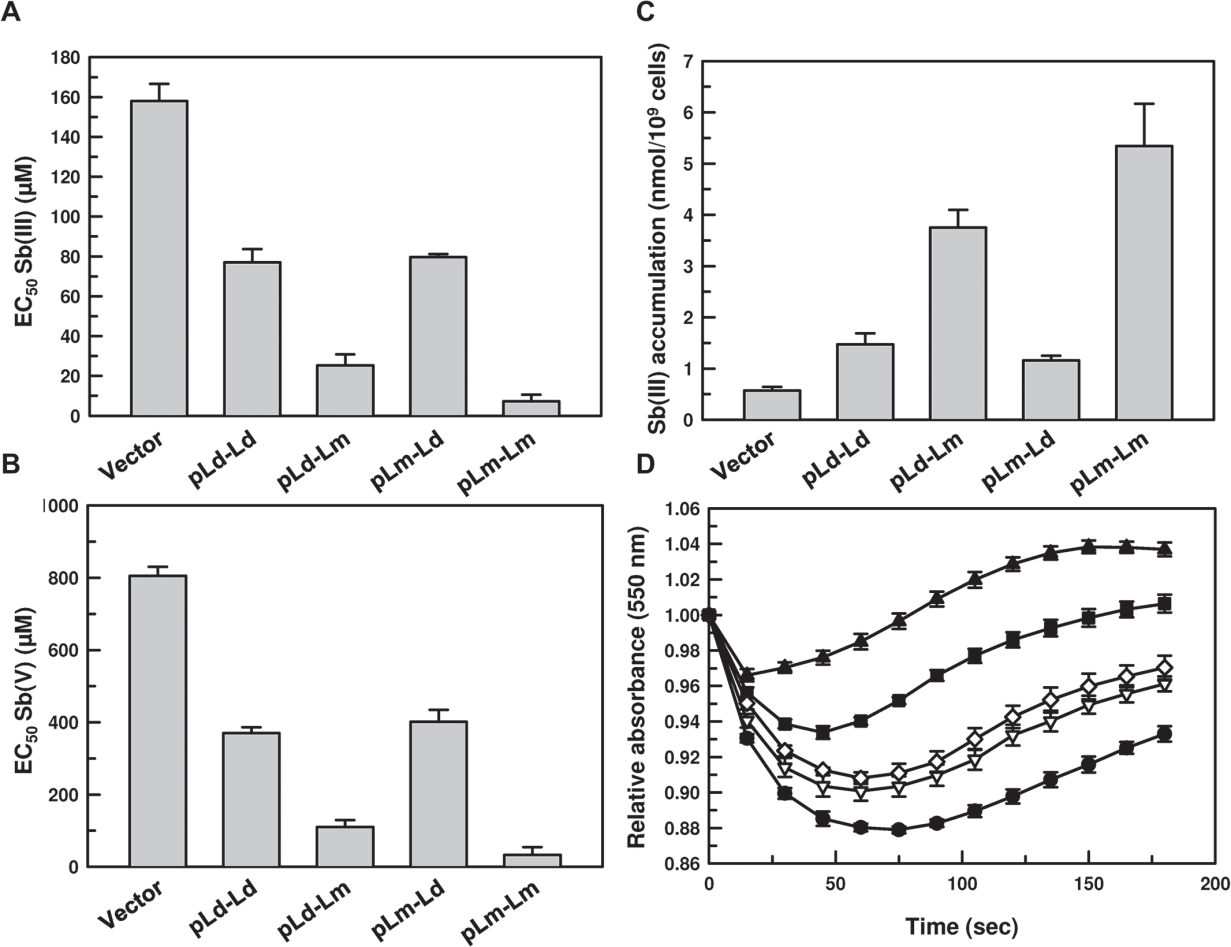
Role of the 3’-UTR in species-specific functionality of AQP1. **A**. Sb(III) sensitivity of *L*. *donovani* promastigotes overexpressing different constructs of AQP1-3’-UTR. **B**. Sb(V) sensitivity of intracellular amastigotes of *L*. *donovani* overexpressing different constructs of AQP1-3’-UTR. **C**. Sb(III) uptake by *L*. *donovani* promastigotes overexpressing different constructs of AQP1-3’-UTR. **D**. Volume regulation of *L*. *donovani* promastigotes overexpressing different constructs of AQP1-3’-UTR;-●- Vector alone,-○- LdAQP1-Ld-3’-UTR (pLd-Ld),-*▼-*LdAQP1-Lm-3’-UTR (pLd-Lm),-△-LmAQP1-Ld-3’-UTR (pLm-Ld), -■-LmAQP1-Lm-3’-UTR (pLm-Lm), Data were expressed as mean ± SE of three independent experiments in triplicate.

Lastly, we argued that if 3’-UTR was driving the species-specific antimony sensitivity, swapping the 3’-UTRs between Ld and Lm-AQP1 should lead to contrasting antimonial sensitivity of a visceral species. Thus we cloned LdAQP1 and LmAQP1 ORFs into pSP72-YHYG-αtubIR with their native 3’-UTRs and also chimeric constructs where the 3’-UTRs were swapped. The four constructs, namely, LdAQP1-Ld3’-UTR, LdAQP1-Lm3’-UTR, LmAQP1-Ld3’-UTR, and LmAQP1-Lm3’-UTR, along with the vector alone control, were transfected into the *L*. *donovani* strain LdBOB. We evaluated the relative copy number of AQP1-3’-UTR-containing plasmids by qPCR using hygromycin phosphotransferase as the target and PTR1 as the housekeeping control, the levels of which were similar in all transfectants (S1B Table). Sb(III) sensitivity of the transfectants were measured in promastigotes (Fig. 8A) and intracellular amastigotes (Fig. 8B). As expected, overexpression of AQP1 made the VL strain hypersensitive to Sb(III) when compared to the vector alone control albeit to a different degree depending on which type of 3’-UTR the ORF had at its 3’ end in both promastigotes and amastigotes. The VL species was 6–24 times more sensitive to Sb(III) in both stages of the parasite whenever Lm-3’-UTR was present at the 3’ end of AQP1 when compared to the Ld-3’-UTR constructs (Fig. 8A and B). A similar trend was observed during intracellular accumulation of Sb(III). *L*. *donovani* promastigotes accumulated significantly more Sb(III) overexpressing AQP1 with Lm-3’-UTR constructs (Fig. 8C). It was interesting to note that the osmoregulatory capacity of the VL species improved considerably with Lm-3’-UTR at the 3’ end of AQP1, whereas promastigotes overexpressing AQP1 with Ld-3’UTR were still poor osmoregulators, although better than the vector alone controls (Fig. 8D). These data convincingly show that 3’-UTR of AQP1 plays a major role in determining the species-specific antimonial sensitivity of *Leishmania* and that the effect is not stage-specific.

## Discussion

Although antimonial drugs are still the first line of treatment against all types of leishmaniasis, treatment failure is often a major cause of concern. The issue of treatment in American CL (ACL) is even more complex because of the factors that often influence the efficacy of the drugs, including the intrinsic and acquired variation in the sensitivities of the different *Leishmania* species [37]. Pentavalent antimonial drugs are the most prescribed treatments for American CL and MCL. The WHO recommends treating ACL with pentavalent antimonials at a dose of 20 mg/kg daily for 28 days [38]. There is no single effective treatment for all species of *Leishmania*. The choice of treatment strategy is based on geographical location and the infecting species [39]. Because of regional and species variability in treatment, doses of antimonials cannot be standardized, and local physicians determine appropriate dosages based on experience [40]. Therefore, it is clinically recognized that species and even strain-specific (regional) antimonial sensitivities are prevalent, which creates major impediments to adopting a single dose strategy for all types of leishmaniasis. There is also molecular and phenotypic heterogeneities that emerged in a natural *L*. *donovani* population from Nepal under antimonial treatment pressure. It has been proposed that each genetically distinct population can develop an antimonial resistant phenotype with a different molecular basis [41]. However, no single mechanism was identified for *L*. *donovani*. On the other hand, *Leishmania* strains causing self-healing CL were proposed to have greater susceptibility towards oxidative stress because they produced less non-protein thiols when compared to the VL species from the Indian subcontinent [15]. Here, we address this controversial issue of species-specific antimonial sensitivity in *Leishmania* by examining four Old World *Leishmania* species such as *L*. *donovani*, *L*. *infantum*, *L tropica* and *L*. *major*, and two New World species, namely *L*. *panamensis* and *L*. *braziliensis*. Our goal was to examine all commonly used drug resistant markers and determine whether there are any direct correlations. It is interesting to note that when some *L*. *infantum* clinical isolates from the Middle East showed CL phenotype, they produced 3–4 fold more AQP1 mRNA [42]. Therefore, the results of our study can be utilized for a larger study with clinical isolates and strains, but outside the scope of the work presented here.

The most commonly used antimonial resistance markers are: (i) MRPA[9]; (ii) thiols [6,11,41]; (iii) an unknown efflux system [8,43]; and (iv) AQP1 [16,19,44,45,46]. We examined carefully each of these factors in all six species. First, there is a correlation between the amount of MRPA mRNA and species-specificity (Fig. 2A). However, this is in contrast to the experimental evidence that MRPA is generally overexpressed in drug resistant *Leishmania* isolates [47]. MRPA is known to transport drug-thiol conjugates in *Leishmania* [9] and higher eukaryotes, including mammals [48,49]. Therefore, if correlated to the species-specific antimonial sensitivity, the *MRPA* mRNA levels in the CL species should have been lower along with lower non-protein thiol levels compared to the VL species. However, there was no discrimination in non-protein thiol levels between the VL and CL species (Fig. 2B), the second commonly used marker in antimonial resistance. Thus, we conclude that MRPA and non-protein thiol levels are not correlated to the species-specific antimonial sensitivity in *Leishmania*. The third factor is the Sb-[TS]_2_ efflux pump, but the rate of efflux is similar in all six species (Fig. 1B). The fourth factor is downregulation of AQP1, and this seems to be driving the species-specific antimonial sensitivity. The two VL species consistently showed less *AQP1* mRNA (Fig. 2C), Sb(III) accumulation (Fig. 1A) and osmoregulatory capacity (Fig. 3). The parameters we studied to determine AQP1 functionality at the protein level and 3’-UTR derived mechanism(s) were extremely difficult to achieve with intracellular amastigotes, the clinically relevant form of the parasite, which need to be generated by in vitro macrophage infections. Axenic amastigote was developed to overcome this limitation, but was not successful for virulent *Leishmania* and specifically the CL species [50]. Also, we showed a similar pattern of antimonial resistance in the intracellular amastigotes of the VL species when compared to the CL species (Table 1). Hence, we chose to work with promastigotes, the vector form of the parasite.

In this study, we established that the species-specific antimonial sensitivity in *Leishmania* is being driven by the regulation of AQP1 at the mRNA level. Additionally, we showed that *AQP1* mRNA is highly unstable in the VL species compared to the CL species (Fig. 4). Since, *Leishmania* does not have any transcriptional control, we determined the role of the *AQP1* 3’-UTRs in the species-specific stabilization of *AQP1* mRNA using the luciferase reporter assay. As expected, the VL species *AQP1* 3’-UTR renders *LUC* mRNA unstable in all six species except in *L*. *major* (Table 2), which was corroborated by their basal *LUC* mRNA levels (Figs. 5A, B, 7A and S7A–S9A) and LUC activity (Figs. 5C, 7B and S7B–S9B). Much less *LUC* mRNA accumulates under the control of the VL 3’-UTRs both in VL and CL *Leishmania* species (Figs. 5B–7A and S7A–S9A), suggesting that there is no species-specific factor responsible for the regulation at the level of *AQP1* mRNA stability, but rather it is the differences in sequences of the 3’-UTRs that make the trans-acting factor(s) more conducive to binding or not and to stabilizing or destabilizing the *AQP1* mRNA. The correlation between *LUC* mRNA levels and LUC protein activity is not linear in *L*. *major* (Fig. 6), *L*. *tropica* (S8 Fig.) and *L*. *panamensis* (S9 Fig.) for the VL 3’-UTRs, suggesting that less mRNA is not necessarily associated with reduced protein levels. This suggests the presence of other factors (or differential expression of these factors) in some CL species that could increase translation rates, despite the fact that amounts of mRNA are low for certain genes including *LUC*. However, levels of AQP1 native mRNA (Fig. 2C) seem to be correlated linearly with protein expression as it corroborated with functional properties of AQP1, such as osmoregulation (Fig. 3) and Sb(III) accumulation (1A). The CL 3’-UTRs are similarly stable in VL species as they are in CL species, suggesting that there is no effect from any species-specific trans-acting factor. It was interesting to note that all 3’-UTRs of *AQP1* provided *LUC* mRNA with the highest level of stability in *L*. *major* (Fig. 7), which is the most antimonial sensitive species under investigation. The most compelling evidence that the 3’-UTRs play a major role in AQP1 species-specific functionality comes from the fact that Lm-3’-UTR made Ld-AQP1 function three times more efficiently compared to its native 3’UTR (Fig. 8) and vice-versa. Additionally, *AQP1* 3’-UTR of *L*. *braziliensis* stabilized *LUC* mRNA, resulting in more LUC activity and protein expression in the VL species than in its native environment (Figs. 5 and 8), leading us to propose that *L*. *braziliensis* might harbor unique factors that are specific for its own AQP1 3’-UTR. However, basal level and stability of AQP1 mRNA in *L*. *braziliensis* were comparable to other CL species, such as *L*. *major* (Figs. 2C and 4). This difference could be attributed to the presence of specific *AQP1* ORF sequences upstream to the 3’-UTRs in the native mRNA. In this context, it is interesting to note that *L*. *braziliensis* and *L*. *panamensis* ORF sequences differ (S2 and S6 Figs.) significantly from the four other species. *L*. *donovani*, *L*. *infantum*, *L*. *major* and *L*. *tropica* ORF sequences are closer to each other, sharing 87% identity among them (S10 and S11 Figs.), whereas the overall identity among all six ORFs is 66% (S2 Fig.). Therefore, the role of AQP1 ORFs in determining the native mRNA stability warrants further research in this direction, which is in progress.

The fundamental question is why there is a species-specific regulation of AQP1 in *Leishmania*. AQP1 is an adventitious facilitator of Sb(III); therefore, this species-specific antimonial resistance driven by AQP1 is a bonus that the VL species enjoy during treatment. Although acquired antimonial resistance in *Leishmania* is multifactorial, it is tempting to speculate that more antimonial resistant cases are observed in VL [13] due to down regulation of AQP1, because it is easy to downregulate something in which the intrinsic trend of higher mRNA instability leading to less production is already in place because the VL species likely does not need efficient osmoregulation. On the other hand, the physiological function of AQP1 is osmoregulation, and we showed that the CL species are better osmoregulators (Fig. 3). Is it possible that the CL species face greater osmotic challenges during vector to host transmission, or vice-versa, and more AQP1 helps them to overcome that barrier? AQPs are also implicated in a number of unrelated physiologic processes and functions, such as lipid metabolism, cell migration, epidermal biology, cell adhesion, and neural signal transduction [51]. Thus, it is also tempting to speculate that species-specific AQP1 expression may help the respective species to find their appropriate niches, resulting in tissue tropism. In VL and most CL cases, a standard single dose of 20mg/kg/day for 28 days of antimonials has been mandated by WHO since 1990 [52]. However, our data emphasize that treating all types of leishmaniasis with the same systemic dosage of antimony may not be a good practice. A lower dosage of antimonials as treatment for the visceral infection (which may be the correct dose for the cutaneous species as they are more sensitive) may have been the reason for the emergence of a more drug-resistant phenotype in that species. These are ambitious and yet intriguing and fundamental questions in *Leishmania* biology. Thus, our novel finding of 3’-UTR driven species-specific regulation of AQP1 is going to drive new approaches in that direction.

## Materials and Methods

### Cell culture

Wild type *Leishmania donovani* strain LdBob (kind gift from Professor Stephen M. Beverley at the Washington University School of Medicine), *L*. *infantum* strain MHOM/MA/67/ITMAP-263, *L*. *major* strain LV39 (kind gift from Professor Marc Ouellette, Laval University, Quebec, Canada), *L*. *braziliensis* strain MHOM/BR/75/M2903 (from ATCC), *L*. *tropica* strain MHOM/IL/67/JERICHO II (from ATCC), and *L*. *panamensis* strain MHOM/PA/71/LS94 (from ATCC) were used in this study. Promastigotes were grown at 26°C as described before [17]. Promastigotes were also grown on blood-agar/ brain heart infusion (BHI) broth biphasic medium containing (a) solid phase of 1.7% agar, 3.7% BHI and defibrinated rabbit blood and (b) liquid phase of 3.7% BHI broth. Human leukemia monocyte cell line THP1 was purchased from ATCC and maintained according to supplier’s instruction.

### Antimony sensitivity assay

Antimony sensitivity of the promastigotes was determined as described previously [17]. Briefly, log phase promastigote cultures were diluted to 2 X10^6^ cells ml^−1^ in a culture medium containing various concentrations of Sb(III) in the form of potassium antimonyl tartrate (Sigma). Following 72 h incubation, cell growth was monitored from the absorbance at 600 nm using a microplate reader (Spectramax 340, Molecular Devices). Percentage survival was plotted against Sb(III) concentrations and EC_50_ was determined using SigmaPlot 11.0. Each assay was performed at least three times in triplicates. Error bars were calculated from the mean ± SE.

Antimony sensitivity of amastigotes inside macrophages was determined after infecting THP1 derived macrophages. Briefly, 5 X10^5^ THP1 cells/well/200 μl of RPMI were seeded in 16 chamber LabTek tissue culture slides (Nunc) and treated with 5 ng/ml phorbol myristate acetate (PMA) for 48h to differentiate into macrophages. Macrophages were infected with stationary phase promastigotes harvested from blood-agar/BHI biphasic medium at a parasite-to-macrophage ratio of 20:1 for 6 hours at 37^o^C with 5% CO_2_. Non-internalized promastigotes were washed away, and infected macrophages were treated with increasing concentrations of Sb(V) in the form of potassium hexahydroxoantimonate (Sigma) for 7 days. Medium was replaced every alternate day, and fresh drug was added. After 7 days, cells were stained with the Giemsa using Quick III Statpak kit (Astral Diagnostics). Numbers of amastigotes per 100 macrophages were determined by light microscopy. EC_50_ was calculated as described for promastigotes. Each assay was performed at least two times in triplicates. Error bars were calculated from the mean ± SE.

### Uptake assay

Log phase *Leishmania* promastigotes were washed twice with phosphate-buffered saline (PBS), pH 7.4 (Invitrogen) and suspended in PBS at a density of 10^8^ cells ml^−1^. Promastigotes were then incubated with 10 μM Sb(III), a 200- μl portion was filtered through a 0.22 μm nitrocellulose filter at different time points (1, 5, 10, 20 and 30 min), and the filter washed once with 5 ml of ice-cold PBS. The filters were digested with 0.4 ml of concentrated HNO_3_ (69–70%) (EM Science) for 1 h at 70^o^C, allowed to cool to room temperature, diluted with high pressure liquid chromatography grade water (Sigma) to produce a final concentration of HNO_3_ of approximately 3%, and then analyzed by a PerkinElmer SCIEX ELAN DRC-e inductively coupled plasma mass spectrometer. Standard solutions were prepared in the range of 0.5–10 p.p.b. in 3% HNO_3_ using antimony standards (Ultra Scientific). Each transport experiment was repeated at least three times with duplicate samples. Error bars were calculated from the mean ± SE.

Membrane vesicles were prepared from promastigotes of each species as described previously [6]. They were rapidly frozen in liquid nitrogen in small aliquots and stored at -80°C until use. The total protein content of the plasma membrane fractions was determined by a filter assay as described previously [53]. ATP dependent uptake of Sb(TS)_2_ was measured in the presence of 10 mM ATP as energy source, as described previously, with a few changes [6]. Briefly, vesicles were added at 0.5 mg of membrane protein/ml and incubated with 0.1 mM of Sb(TS)_2_ in a buffer containing 75 MM Hepes-KOH, pH 7.0/0.15 M KCl. Reaction was started by the addition of ATP at room temperature. At the indicated intervals, samples (0.1 ml) were removed and filtered on wet 0.22 μm nitrocellulose filter, and the filter washed once with 5 ml of ice-cold PBS. The membranes were digested with 70% HNO_3,_ and total Sb content was measured by ICP-MS as described above. Each transport experiment was repeated at least two times with triplicate samples. Error bars were calculated from the mean ± SE

### Cell volume measurements

Relative changes in cell volume following the induction of hypo-osmotic shock were measured as described earlier [54]. Briefly, log phase promastigotes were washed twice in PBS and re-suspended at a density of 10^9^ cells ml^−1^. One-hundred-microliter portions of the cell suspension were transferred to a microtiter plate. Hypo-osmotic shock was induced by dilution of the isotonic cell suspension with an equal volume of deionized water, and the absorbance at 550 nm was recorded every 15 sec for 3 min in a microplate reader (Spectramax 340, Molecular Devices). A decrease in absorbance corresponds to an increase in cell volume. Isosmotic control experiments consisted of dilution of cell suspensions with appropriate volumes of isosmotic buffer. All hypo-osmotic shock experiments were conducted at a final osmolarity of 150 mOsm (1:1 dilution of isosmotic buffer and water). Each experiment was repeated at least three times in triplicate. Error bars were calculated from the mean ± S.E.

### Isolation of nucleic acids

Genomic DNA was isolated using DNAzol reagent (Life Technologies). Total RNA from *Leishmania* promastigotes was isolated using TRIZOL reagent (Life Technologies) according to the manufacturer’s protocol. DNA was removed from total RNA preparation using TURBO DNA-free Kit (Ambion) according to the manufacturer’s instructions. Integrity of total RNA preparations was confirmed by denaturing agarose gel electrophoresis.

### Mapping and cloning of 3’-UTR

The AQP1 3’-UTR fragments from all six *Leishmania* species were mapped using 3’-RACE kit (Invitrogen) with 2 μg of total RNA as template according to the manufacturer’s protocol. *AQP1* gene specific primers (S2 Table) were designed according to genome sequences in TriTrypDB. The amplified 3’-UTR fragments for all the species were cloned into pGEMT-Easy vector (Promega) according to the manufacturer’s instructions and sequenced (Eton Biosciences). As the database sequence of AQP1 ORF of *L*. *tropica* is not available, we cloned its AQP1 ORF from *L*. *tropica* genomic DNA using primers (sense 5’- GAATTC ATGAACTCTCCTACAACCATGCC-3’ and antisense 5’- GCGGCCGC CTAACAGCTGGGCGGAATGAT-3’) designed against *L*. *major* AQP1 ORF. This *L*. *tropica* AQP1 ORF sequence was used to design primers (S2 Table) for subsequent mapping of *L*. *tropica* AQP1 3’-UTR by 3’-RACE as described above.

### Plasmid construction and transfection

Previously described [28] luciferase (LUC) expression vector for *Leishmania* pSPYNEOαLUC is referred to as LUC-control or pLUC in this study. The full-length 3′UTR of AQP1 and 200 base pairs beyond the 3’ end of the poly(A) site was PCR amplified from genomic DNA of each species using AccuPrime Taq DNA Polymerase (Invitrogen) and primers (S2 Table) with BamHI or SalI restriction sites inserted at 5’ and 3’ ends. To clone a similar 200 base pair sequence from *L*. *tropica*, a 555 base pairs fragment downstream to the poly(A) site was cloned using *L*. *tropica* genomic DNA using the sense primer (5’- GATGAGTGCACACGGCGTACTTC-3’) designed against *L*. *tropica* AQP1 ORF and the antisense primer (5’- ATGGTCGTACCACGCAAAGTCACC-3’) designed against *L*. *major* dtatbase (TriTrypDB) sequence. The sequence of this fragment was used to design primers (S2 Table) for subsequent cloning of *L*. *tropica* full-length 3′UTR of AQP1 and 200 base pairs downstream to the 3’ end of the poly(A) site. PCR products were cloned (cloning primers are described in S2 Table) into the pGEMT-Easy vector (Promega) and sequenced (Eton Bioscience) as described above. Different LUC-chimeric constructs were generated by digesting the pGEMT-Easy clones with BamHI or SalI (New England Biolab) and subcloned into the BamHI or SalI sites of LUC gene (3’ end) in the vector pSPYNEOαLUC, respectively. Directions of the cloned DNA segments were confirmed by sequencing. All plasmid constructs with forward orientation in respect to *LUC* open reading frame (ORF) were purified using QIAprep Spin Miniprep Kit (Qiagen). Purified plasmid constructs were transfected into Leishmania by electroporation. Briefly, stationary phase promastigotes were washed and resuspended in ice cold electroporation buffer (21 mM HEPES, 150 μM NaCl, 5 mM MgCl_2_, 120 mM KCl, 0.7 mM NaH_2_PO_4_, 6 mM glucose) at a density of 10^8^ cells/ml. Three hundred microliters of resuspended cells were transferred to 0.2 cm electroporation cuvette (Bio-rad) with 10 μg of plasmid DNA. Cells were electroporated in Gene Pulser (Bio-rad) at 0.45 kV and 500 μF. All transfectants were selected and maintained in presence of 60 μg/ml geneticin (G418) (Invitrogen). The relative copy numbers of different *LUC* constructs in different transfectants were determined by qPCR using total genomic DNA from each transfectant. The relative abundance of target amplicons between samples was estimated by the 2^−ΔΔCT^ method [55] using pteridine reductase 1 (PTR 1) as loading control. The processing of 3’ end of each 3’-UTR expressing from episomal copy was reconfirmed by 3’-RACE PCR as described above with gene specific primers designed from *LUC* ORF (S2 Table).

The full-length ORF of *AQP1* was PCR amplified from genomic DNA of *L*. *donovani* (LdAQP1) and *L*. *major* (LmAQP1) using AccuPrime Taq DNA Polymerase (Invitrogen) and primers (S2 Table) with BamHI and XbaI restriction sites inserted at 5’ and 3’ ends respectively. The full-length 3′-UTR of *AQP1* and 200 base pairs beyond the 3’ end of the poly(A) site were similarly PCR amplified using genomic DNA of *L*. *donovani* (Ld3’-UTR) and *L*. *major* (Lm3’-UTR) and primers (S2 Table) with XbaI restriction site inserted at 5’ and 3’ ends. PCR products were cloned into the pGEMT-Easy vector and sequenced as described above. pGEMT-Easy clones containing LdAQP1 and LmAQP1 were digested with BamH1 and XbaI (partial digestion with BamH1 for Ld*AQP1*) and the resultant *AQP1* ORF fragments were subcloned into pSP72-YHYG-αtubIR to generate LdAQP1-pSP72-YHYG-αtubIR and LmAQP1-pSP72-YHYG-αtubIR constructs. pGEMT-Easy clones containing Ld3’-UTR and Lm3’-UTR were digested with Xba1 and the resultant 3’UTR fragments were subcloned into both LdAQP1-pSP72-YHYG-αtubIR and LmAQP1-pSP72-YHYG-αtubIR constructs linearized with XbaI to generate LdAQP1-Ld3’-UTR-pSP72-YHYG-αtubIR (pLd-Ld), LdAQP1-Lm3’-UTR-pSP72-YHYG-αtubIR (pLd-Lm), LmAQP1-Ld3’-UTR-pSP72-YHYG-αtubIR (pLm-Ld) and LmAQP1-Lm3’-UTR-pSP72-YHYG-αtubIR (pLm-Lm) constructs (AQP1-3’-UTR constructs). Directions and integrity of the cloned DNA segments in *AQP1-*3’-UTR constructs were confirmed by sequencing. pSP72-YHYG-αtubIR (vector) and all *AQP1-*3’-UTR constructs were purified and transfected into *L*. *donovani* and *L*. *major* promastigotes as described above. All transfectants were selected and maintained in the presence of 300 μg/ml hygromycin B (Invitrogen). The relative copy numbers of different constructs in different transfectants were determined against hygromycin phosphotransferase gene by qPCR as described earlier.

### qPCR and qRT-PCR analyses

cDNA synthesis was carried out using 500 ng of total RNA and AccuScript High Fidelity 1^st^ strand cDNA synthesis kit (Agilent) according to the manufacturer’s instructions. The first-strand cDNA reaction mix was treated with 0.25N NaOH at 65^0^C for 30 minutes to degrade the template RNA molecules. The reaction mix was neutralized using equimolar hydrochloric acid and purified using Qiagen PCR purification kit according to the manufacturer’s instructions. For qPCR, 10 ng of genomic DNA and for qRT-PCR, 2 μl of diluted purified cDNA reaction corresponding to 6 ng of template RNA, were used in a 10 μl reaction containing forward and reverse primers for the target genes (S2 Table) and 1X iQSYBR Green supermix (Bio-rad). The reactions were run on an Eppendorf Realplex^2^ PCR machine in the following thermal cycling conditions: initial denaturation at 95^0^C for 3 minutes followed by 40 cycles of 95^0^C for 15 sec and 65^0^C for 20 sec. A final melting curve analysis was performed for each reaction to confirm that the PCR generated a single amplification product. Multiple primer sets against each target were designed using PrimerQuest software (Intergrated DNA technologies; http://www.idtdna.com/Primerquest/Home/Index?Display=SequenceEntry) and tested for their efficiency using the afore-mentioned thermal cycling conditions; the set(s) of primers showing efficiency between 98% to 102% were included in the current study. The relative abundance of target amplicons between samples was estimated using glyceraldehyde 3-phosphate dehydrogenase (GAPDH) as loading control by the 2^−ΔΔCT^ method [55]. Error bars were calculated from the mean ± SD of three independent experiments in triplicate. Similar expression levels of GAPDH in all six species were confirmed using β-tubulin as the loading control (S3 Table).

### RNA stability assays

To determine the half-lives of *AQP1* or *LUC* mRNA, mid-log phase promastigotes were treated with sinefungin (5 μM) (Sigma) for 15 min followed by incubation with 10 μg/ml of actinomycin D (Sigma) to arrest trans-splicing and transcription, respectively. Cells were harvested just before adding actinomycin D and considered as the zero min time point. Subsequently, cells were harvested at 15, 30, 45, 60 and 120 min; an additional eight min of processing time was added while presenting the data. Total RNA isolation and qRT-PCR analysis were performed as described above using *AQP1* or *LUC* ORF specific primers (S2 Table). Error bars were calculated from the mean ± SD of three independent experiments in triplicate.

### Luciferase activity assay

Mid-log phase promastigotes (5X10^6^) were lysed with 50 μl of lysis buffer (62.5 mM Tris-phosphate p^H^ 7.8, 5 mM DTT, 2.5% Triton X-100, 25% glycerol). A 10 μl of lysate was used to estimate the luciferase activity using Luc-Screen Extended-Glow Luciferase Reporter Gene Assay System (Life Technologies) according to the manufacturer’s instruction. Error bars were calculated from the mean ± SE of three independent experiments in triplicate.

### Western blot analysis

Whole cell lysates were prepared by lysing 1x10^7^ promastigotes from each transfectant in 100 μl of 1x Laemmli’s buffer [56]. 10 μl lysate was used to fractionate proteins on 12% SDS-PAGE. Fractionated proteins were electroblotted on nitrocellulose membranes (Whatman) and probed sequentially with polyclonal goat anti-luciferase (Promega) and monoclonal mouse anti-α-tubulin (Sigma). The labeling was visualized with horseradish peroxidase-conjugated mouse anti-goat (Pierce) and rabbit anti-mouse (Abcam) respectively using a Western Lightning Chemiluminescence Reagent Plus system (PerkinElmer). Amount of luciferase expression relative to cells transfected with pSPYNEOαLUC was estimated by densitometric analysis using ImageJ software followed by normalization against the amount of α-tubulin of the respective cells. Error bars were calculated from the mean ± SE of two independent experiments.

### Estimation of total thiols

The level of total intracellular non-protein thiol was measured in deproteinized cell extracts as described previously [6]. Briefly, log phase promastigotes (6 x 10^8^) were harvested, washed with PBS and suspended in 0.6 ml of 25% tricholoracetic acid. Cell debris and denatured protein were removed by centrifugation at 16,000g for 20 min at 4°C after 10 min incubation on ice. The thiol content of the supernatant solution was determined using 0.6 mM 5,5'-dithio-bis(2-nitrobenzoic acid) (DTNB) in 0.2 M sodium phosphate buffer (pH 8.0). The concentration of 2-nitro-5-thiobenzoate (TNB), derivatives of non-protein thiol-DTNB reaction, was estimated spectrophotometrically at 412 nm. The concentration of total thiols in the test supernatants was estimated against a standard curve of cysteine. Error bars were calculated from the mean ± SE of three independent experiments in triplicate.

## Supporting Information

S1 Fig Alignment of the protein sequences of AQP1 from six *Leishmania* species. The protein source and GenBank accession numbers of the aligned sequences are *L*. *donovani* (ABQ84980); *L*. *infantum* (CAM70318); *L*. *major* (XP_001684986); *L*. *tropica* (not annotated), *L*. *braziliensis* (ADU56881); *L*. *panamensis* (not annotated). Sequences were aligned using ClustalW2 and Boxshade server. The dashes indicate the gaps introduced to maximize sequence alignment. The black and grey regions indicate sequence identity and sequence similarity, respectively.
(PDF)

S2 Fig Alignment of the ORF sequences of AQP1 from six *Leishmania* species. The protein source and GenBank accession numbers of the aligned sequences are *L*. *donovani* (ABQ84980); *L*. *infantum* (CAM70318); *L*. *major* (XP_001684986); *L*. *tropica* (not annotated), *L*. *braziliensis* (ADU56881); *L*. *panamensis* (not annotated). Sequences were aligned using ClustalW2 and Boxshade server. The dashes indicate the gaps introduced to maximize sequence alignment.
(PDF)

S3 Fig Alignment of the 3’-UTR sequences of AQP1 mRNA from six *Leishmania* species. The 3’-UTRs from each species were cloned and sequenced as described in the materials and methods. Sequences were aligned using ClustalW2 and Boxshade server. The dashes indicate the gaps introduced to maximize sequence alignment.
(PDF)

S4 Fig Alignment of the 3’-UTR sequences of AQP1 mRNA from *L. donovani* and *L. infantum*. The 3’-UTRs from each species were cloned and sequenced as described in the materials and methods. Sequences were aligned using ClustalW2 and Boxshade server. The dashes indicate the gaps introduced to maximize sequence alignment.
(PDF)

S5 Fig Alignment of the 3’-UTR sequences of AQP1 mRNA from *L. major* and *L. tropica*. The 3’-UTRs from each species were cloned and sequenced as described in the materials and methods. Sequences were aligned using ClustalW2 and Boxshade server. The dashes indicate the gaps introduced to maximize sequence alignment.
(PDF)

S6 Fig Alignment of the 3’-UTR sequences of AQP1 mRNA from *L. braziliensis* and *L. panamensis*. The 3’-UTRs from each species were cloned and sequenced as described in the materials and methods. Sequences were aligned using Clustal omega and Boxshade server. The dashes indicate the gaps introduced to maximize sequence alignment.
(PDF)

S7 Fig Effect of *AQP1* 3’-UTR from different species of *Leishmania* on levels of *LUC* mRNA, protein expression and activity when transfected in promastigotes of *L*. *infantum*. **A**. *LUC* mRNA levels: Total RNA was isolated from promastigotes of *L*. *infantum* expressing different chimeric constructs of *LUC* and *LUC* mRNA expression levels were estimated using qPCR. Relative (with respect to *LUC*) *LUC* mRNA expression levels were calculated using 2^-ΔΔCt^ method. Data were expressed as mean ± SD of three independent experiments in triplicate. **B**. LUC activity and expression: Estimation of LUC activity (□) was carried out using whole cell lysates. Percent LUC activity was calculated keeping vector control at 100%. Data were expressed as mean ± SE of three independent experiments in triplicate. **C**. Representative western blot analysis of transfected promastigotes: Whole cell (1 x 10^6^/lane) lysates of different transfectants were fractionated on SDS-PAGE and blotted onto nitrocellulose membrane. Levels of LUC expressions were detected using an anti-luciferase antibody. α-tubulin was used as loading control. Lanes: 1. pLUC, 2. pLUC-Ld, 3. pLUC-Li, 4. pLUC-Lm, 5. pLUC-Lt, 6. pLUC-Lb, and 7. pLUC-Lp. Amount of luciferase expression (■) relative to cells transfected with pSPYNEOαLUC was estimated by densitometric analysis using ImageJ software followed by normalization against the amount of α-tubulin of the respective cells. Error bars were calculated from the mean ± SE of two independent experiments. *Ld- L*. *donovani*, *Li- L*. *infantum*, *Lm- L*. *major*, *Lt- L*. *tropica*, *Lb- L*. *braziliensis*, *Lp- L*. *panamensis*.
(PDF)

S8 Fig Effect of *AQP1* 3’-UTR from different species of *Leishmania* on levels of *LUC* mRNA, protein expression and activity when transfected in promastigotes of *L*. *tropica*. **A**. *LUC* mRNA levels: Total RNA was isolated from promastigotes of *L*. *tropica* expressing different chimeric constructs of *LUC*, and *LUC* mRNA expression levels were estimated using qPCR. Relative (with respect to *LUC*) *LUC* mRNA expression levels were calculated using 2^-ΔΔCt^ method. Data were expressed as mean ± SD of three independent experiments in triplicate. **B**. LUC activity and expression: Estimation of LUC activity (□) was carried out using whole cell lysates. Percent LUC activity was calculated keeping vector control at 100%. Data were expressed as mean ± SE of three independent experiments in triplicate. **C**. Representative Western blot analysis of transfected promastigotes: Whole cell (1 x 10^6^/lane) lysates of different transfectants were fractionated on SDS-PAGE and blotted onto nitrocellulose membrane. Levels of LUC expressions were detected using an anti-luciferase antibody. α-tubulin was used as loading control. Lanes: 1. pLUC, 2. pLUC-Ld, 3. pLUC-Li, 4. pLUC-Lm, 5. pLUC-Lt, 6. pLUC-Lb, and 7. pLUC-Lp. Amount of luciferase expression (■) relative to cells transfected with pSPYNEOαLUC was estimated by densitometric analysis using ImageJ software followed by normalization against the amount of α-tubulin of the respective cells. Error bars were calculated from the mean ± SE of two independent experiments. *Ld- L*. *donovani*, *Li- L*. *infantum*, *Lm- L*. *major*, *Lt- L*. *tropica*, *Lb- L*. *braziliensis*, *Lp- L*. *panamensis*.
(PDF)

S9 Fig Effect of *AQP1* 3’-UTR from different species of *Leishmania* on levels of *LUC* mRNA, protein expression and activity when transfected in promastigotes of *L*. *panamensis*. **A**. *LUC* mRNA levels: Total RNA was isolated from promastigotes of *L*. *panamensis* expressing different chimeric constructs of *LUC* and *LUC* mRNA expression levels were estimated using qPCR. Relative (with respect to *LUC*) *LUC* mRNA expression levels were calculated using 2^-ΔΔCt^ method. Data were expressed as mean ± SD of three independent experiments in triplicate. **B**. LUC activity and expression: Estimation of LUC activity (□) was carried out using whole cell lysates. Percent LUC activity was calculated keeping vector control at 100%. Data were expressed as mean ± SE of three independent experiments in triplicate. **C**. Representative western blot analysis of transfected promastigotes: Whole cell (1 x 10^6^/lane) lysates of different transfectants were fractionated on SDS-PAGE and blotted onto nitrocellulose membrane. Levels of LUC expressions were detected using an anti-luciferase antibody. α-tubulin was used as loading control. Lanes: 1. pLUC, 2. pLUC-Ld, 3. pLUC-Li, 4. pLUC-Lm, 5. pLUC-Lt, 6. pLUC-Lb, and 7. pLUC-Lp. Amount of luciferase expression (■) relative to cells transfected with pSPYNEOαLUC was estimated by densitometric analysis using ImageJ software followed by normalization against the amount of α-tubulin of the respective cells. Error bars were calculated from the mean ± SE of two independent experiments. *Ld- L*. *donovani*, *Li- L*. *infantum*, *Lm- L*. *major*, *Lt- L*. *tropica*, *Lb- L*. *braziliensis*, *Lp- L*. *panamensis*.
(PDF)

S10 Fig Phylogenetic tree analysis of AQP1 ORF sequences from different *Leishmania* species. Multiple sequence alignment for the ORF (open reading frame) was done using MUSCLE. This helps to eliminate poorly aligned positions and divergent regions. Phylogeny was built using PhyML program and tree was made using Figtree. Midpoint rooted tree depicts two different clades for mucocutaneous species (*L*.*braziliensis* and *L*.*panamensis*), cutaneous (*L*.*major* and *L*.*tropica*) and visceral species (*L*.*donovani* and *L*.*infantum*) based on their ORF sequences. According to the tree, there is 0.8% difference between the ORF sequences of *L*.*braziliensis* and *L*.*panamensis*. Sequences are similar for both the visceral species, whereas there is 2.8% difference between *L*.*major* and *L*.*tropica* sequences from the branch node.
(PDF)

S11 Fig Alignment of the ORF sequences of AQP1 from four *Leishmania* species. The protein source and GenBank accession numbers of the aligned sequences are *L*. *donovani* (ABQ84980); *L*. *infantum* (CAM70318); *L*. *major* (XP_001684986); *L*. *tropica* (not annotated). Sequences were aligned using ClustalW2 and Boxshade server. The dashes indicate the gaps introduced to maximize sequence alignment.
(PDF)

S12 Fig Stability of *LUC* mRNA in promastigotes of different species of *Leishmania*. Promastigotes of different species of *Leishmania* (each transfected with six different AQP1 3’-UTR chimeric constructs as described in Fig. 5A) were exposed to sinefungin followed by actinomycin D. Cells were harvested just before the exposure of actinomycin D (0 minute) and at different time points after the exposure with actinomycin D. Total RNA was isolated, and *LUC* mRNA levels were estimated using qPCR. Relative (with respect to 0 minute) *LUC* mRNA levels were calculated using 2^-ΔΔCt^ method. Data were expressed as mean ± SD of three independent experiments in triplicate.-●- pLUC,-○- pLUC-Ld,-*▼-* pLUC-Li,-△-pLUC-Lm,-■- pLUC-Lt,-□- pLUC-Lb,-◆- pLUC-Lp. **A:** *L*. *donovani*, **B:** *L*. *infantum*, **C**. *L*. *major*, **D**. *L*. *tropica*, **E**. *L*. *braziliensis*, and **F**. *L*. *panamensis*.
(PDF)

S1 Table A. Copy numbers of *LUC* in presence of species-specific 3’ UTRs of AQP1 mRNA at the 3’ end. **B**. Copy numbers of AQP1-3’-UTR constructs.
(PDF)

S2 Table List of primers used in this study. (PDF)

S3 Table Relative (to *L*. *donovani*) expression of GAPDH mRNA with respect to β tubulin mRNA in different species of *Leishmania*. (PDF)
