# Supplementary material for: Species-Specific Antimonial Sensitivity in Leishmania Is Driven by Post-Transcriptional Regulation of AQP1
Source: PLoS Negl Trop Dis. 2015 Feb 25;9(2):e0003500. doi: 10.1371/journal.pntd.0003500 (PMC4340957; doi:10.1371/journal.pntd.0003500)
Supplement: S1 Fig — The protein source and GenBank accession numbers of the aligned sequences are L. donovani (ABQ84980); L. infantum (CAM70318); L. major (XP_001684986); L. tropica (not annotated), L. braziliensis (ADU56881); L. panamensis (not annotated). Sequences were aligned using ClustalW2 and Boxshade server. The dashes indicate the gaps introduced to maximize sequence alignment. The black and grey regions indicate sequence identity and sequence similarity, respectively. (PDF) [file pntd.0003500.s001.pdf]

Figure S1

```
L. braziliensis     1 -MSVDRTLYPSYEGEVQLYLQKEDPEGMAIENHMDEDQ--QQRQKRDLTAQDTWPLYRYR
L. panamensis       1 -MSVDRTPYPSYEGEVQLYLQKEDPEGMAIENHMDEDQ--QQRQKRDLTAQDTWPLYRYR
L. major            1 MNSPTTMPLQCHDAETQLYVDKESPEGLPIGNQMHEEEEDQHESKRNFMSQNRWPLYRYR
L. tropica          1 MNSPTTMPPQCYEAEVQLCMDKEDPAGVPSGNRMHEEEEGQLENKRNFTLQNRWPLYKYR
L. donovani         1 MNSPTSTPPACYDAEVQLYMDKEDPEGVPIQNQMHEEEQGQLEGKRNFTSQNRWPLYKYR
L. infantum         1 MNSPTSTPPACYDAEVQLYMDKEDPEGVPIQNQMHEEEQGQLEGKRNFTSQNRWPLYKYR

L. braziliensis    58 WWIREYVAEFFGSFFLLSFGIGVTATTTFHAGNTASFQTNVSYLAITLGWGFGLAIALFI
L. panamensis      58 WWIREYVAEFFGSFFLLSFGIGVTATTTFHAGNTASFQTNVSYLAITLGWGFGLAIALFI
L. major           61 WRLREYVAEFFGTFFLVTFGTGVVATTVFHGGTTAMYQSNSSYLAITFGWAFGLAISLFL
L. tropica         61 WRIREYVAEFFGTFFLVTFGTGVIATTVFHAGNAASYQSNSSYLAITFGWGFGLTIGLFL
L. donovani        61 WWLREYVAEFFGTFFLVTFGTGVIATTVFHAGNAASYQSNSSYMAITFGWGFGLTIGLFL
L. infantum        61 WWLREYVAEFFGTFFLVTFGTGVIATTVFHAGNAASYQSNSSYMAITFGWGFGLTIGLFL

L. braziliensis   118 TMGVSGGHLNPAVTLANCAFGAFPWRKAPGFMLAQLLGAILGAANVYGLFKQHFDDAGVM
L. panamensis     118 TMGVSGGHLNPAVTLANCAFGAFPWRKAPGFMLAQLLGAILGAANVYGLFKQHFDDAAVM
L. major          121 SMAVSGGHLNPAVTLANCVFGTFPWVKLPGYFLAQFLGGFVGAANTYVLFKSHFDEAEKR
L. tropica        121 SMAVSGGHLNPAVTLANCVFGTFPWIKLPGYFLAQFLGGFVGAANTYMLFKSHFDDAQKM
L. donovani       121 SMAVSGGHLNPAVTLANCVFGAFPWIKLPGYFLAQFLGGLVGAANTYGLFKSHFDDAQKA
L. infantum       121 SMAVSGGHLNPAVTLANCVFGAFPWIKLPGYFLAQFLGGLVGAANTYGLFKSHFDDAQKA

L. braziliensis   178 LLPNETMASKFSGVFVTYPNVSNVFAVWSEIFNTMVLMMGILAINDNRMTPADGYKPVAV
L. panamensis     178 LLPNETMASKFSGVFVTYPNVSNVFAVWSEIFNTMVLMMGILAINDNRMTPADGYKPVAV
L. major          181 LLLNETMASKYGGIFATYPNVANTYAVWSEVFNTMALMMGILAITDARMTPAVDYKPVAI
L. tropica        181 LSPSETMASKYSGIFATYPNVANTYAVWSEVFNTMALMMGILAITDPRMTPAVNYKPVAI
L. donovani       181 LLPNETMASKYSGIFATYPNVANTYAVWSEVFNTMALMMGILAITDPRMTPAVNYKPVAI
L. infantum       181 LLPNETMASKYSGIFATYPNVANTYAVWSEVFNTMALMMGILAITDPRMTPAVNYKPVAI

L. braziliensis   238 GLLLFVIGITTGINSGYALNPTRDLGPRIFTAMLWGKEPFTLHGYYFWIPIVGPIAGALL
L. panamensis     238 GLLLFVIGITTGINSGYALNPTRDLGPRIFTAMLWGKEPFILHGYYFWIPIVGPIAGALL
L. major          241 GLLLFVIGIASGINSSYGLNPARDLSPRILSAMLWGSEPFTLHSYYFWIPLVVPFVGALF
L. tropica        241 GLLLFVIGITSGINSSYGLNPARDLSPRILSAILWGSEPFTLYSHYFWIPLVAPFVGALL
L. donovani       241 GLLLFVIGITSGINSSYGLNPARDLSPRILSAMLWGSEPFTLYSYYFWIPLVAPFVGALL
L. infantum       241 GLLLFVIGITTGINSSYGLNPARDLSPRILSAMLWGSEPFTLYSYYFWIPLVAPFVGALL

L. braziliensis   298 GMFLYVFCIIPSGA
L. panamensis     298 GMFLYVFCIIPSGA
L. major          301 GMFLYVFFIIPPSC
L. tropica        301 GMFLYVFFIIPPSC
L. donovani       301 GMFLYVFFIIPPNF
L. infantum       301 GMFLYVFFIIPPNF
```
