# Supplementary material for: Species-Specific Antimonial Sensitivity in Leishmania Is Driven by Post-Transcriptional Regulation of AQP1
Source: PLoS Negl Trop Dis. 2015 Feb 25;9(2):e0003500. doi: 10.1371/journal.pntd.0003500 (PMC4340957; doi:10.1371/journal.pntd.0003500)
Supplement: S2 Fig — The protein source and GenBank accession numbers of the aligned sequences are L. donovani (ABQ84980); L. infantum (CAM70318); L. major (XP_001684986); L. tropica (not annotated), L. braziliensis (ADU56881); L. panamensis (not annotated). Sequences were aligned using ClustalW2 and Boxshade server. The dashes indicate the gaps introduced to maximize sequence alignment. (PDF) [file pntd.0003500.s002.pdf]

Figure S2

```
L. braziliensis    1 ATGAGCGTCGACAGA---ACCCTCTATCCCTCCTATGAGGGCGAAGTCCAGCTCTACCTG
L. panamensis      1 ATGAGCGTCGACAGA---ACCCCCTATCCCTCCTATGAGGGCGAAGTCCAGCTCTACCTG
L. major           1 ATGAACTCTCCTACAACCATGCCTCTCCAGTGCCACGATGCCGAGACTCAGCTGTACGTG
L. donovani        1 ATGAACTCTCCTACAAGCACACCTCCCGCGTGCTACGATGCCGAGGTTCAGCTGTACATG
L. infantum        1 ATGAACTCTCCTACAAGCACACCTCCCGCGTGCTACGATGCCGAGGTTCAGCTGTACATG
L. tropica         1 ATGAACTCTCCTACAACCATGCCTCCCCAGTGCTACGAGGCCGAGGTTCAGCTGTGCATG

L. braziliensis   58 CAGAAAGAGGACCCGGAAGGGATGGCGATTGAAAACCACATGGACGA------GGATCAG
L. panamensis     58 CAGAAAGAGGACCCGGAAGGGATGGCGATTGAAAACCACATGGACGA------GGATCAG
L. major          61 GACAAGGAGAGCCCGGAAGGGCTCCCCATTGGAAACCAAATGCATGAGGAAGAGGAGGAC
L. donovani       61 GACAAGGAGGACCCGGAAGGGGTCCCCATTCAAAACCAAATGCACGAGGAAGAGCAGGGA
L. infantum       61 GACAAGGAGGACCCGGAAGGGGTCCCCATTCAAAACCAAATGCACGAGGAAGAGCAGGGA
L. tropica        61 GACAAGGAGGACCCGGCAGGGGTCCCCAGTGGAAACCGAATGCATGAGGAAGAGGAGGGA

L. braziliensis  112 CAGCAGAGGCAGAAACGAGACTTGACGGCGCAGGATACGTGGCCTCTTTACCGGTACCGA
L. panamensis    112 CAGCAGAGGCAGAAACGAGACTTGACGGCGCAGGATACGTGGCCTCTTTACCGGTACCGA
L. major         121 CAACACGAGAGTAAGCGAAACTTCATGTCGCAGAACAGATGGCCCCTCTACAGATACCGA
L. donovani      121 CAGCTCGAGGGTAAAAGGAACTTCACGTCGCAGAACAGGTGGCCCCTGTACAAATACCGA
L. infantum      121 CAGCTCGAGGGTAAAAGGAACTTCACGTCGCAGAACAGGTGGCCCCTGTACAAATACCGA
L. tropica       121 CAGCTCGAGAATAAAAGGAACTTCACGTTGCAGAACAGGTGGCCCCTCTACAAATACCGA

L. braziliensis  172 TGGTGGATACGCGAGTACGTTGCCGAGTTCTTCGGATCGTTTTTCCTCCTTTCCTTTGGC
L. panamensis    172 TGGTGGATACGCGAGTACGTTGCCGAGTTCTTCGGATCGTTTTTCCTCCTTTCCTTTGGC
L. major         181 TGGCGGCTACGCGAGTATGTTGCCGAGTTCTTCGGAACGTTTTTCCTCGTCACCTTTGGA
L. donovani      181 TGGTGGCTACGCGAGTATGTTGCCGAGTTCTTCGGCACGTTTTTCCTCGTCACCTTTGGA
L. infantum      181 TGGTGGCTACGCGAGTATGTTGCCGAGTTCTTCGGCACGTTTTTCCTCGTCACCTTTGGA
L. tropica       181 TGGCGGATCCGCGAGTATGTTGCCGAGTTCTTCGGAACGTTTTTCCTCGTCACCTTTGGA

L. braziliensis  232 ATCGGCGTCACCGCAACCACCACGTTTCACGCCGGTAACACAGCCTCTTTTCAGACAAAC
L. panamensis    232 ATCGGCGTCACCGCAACCACCACGTTTCACGCCGGTAACACAGCCTCTTTTCAGACAAAC
L. major         241 ACCGGCGTCGTTGCGACCACTGTGTTTCATGGCGGTACCACTGCCATGTACCAGTCCAAC
L. donovani      241 ACCGGCGTCATTGCTACCACTGTCTTTCACGCCGGTAACGCTGCAAGCTACCAGTCCAAC
L. infantum      241 ACCGGCGTCATTGCTACCACTGTCTTTCACGCCGGTAACGCTGCAAGCTACCAGTCCAAC
L. tropica       241 ACCGGCGTCATTGCGACCACTGTGTTTCACGCCGGTAACGCCGCCAGCTACCAGTCCAAC

L. braziliensis  292 GTGAGCTACCTCGCAATCACCTTGGGATGGGGTTTTGGGCTTGCCATTGCCCTTTTCATA
L. panamensis    292 GTGAGCTACCTCGCAATCACCTTGGGATGGGGTTTTGGGCTTGCCATTGCCCTTTTCATA
L. major         301 TCAAGCTACCTGGCCATCACCTTTGGCTGGGCATTTGGGCTCGCCATCAGCCTTTTCCTG
L. donovani      301 TCCAGCTACATGGCCATCACCTTTGGCTGGGGGTTTGGGCTCACTATCGGCCTTTTCCTG
L. infantum      301 TCCAGCTACATGGCCATCACCTTTGGCTGGGGGTTTGGGCTCACTATCGGCCTTTTCCTG
L. tropica       301 TCCAGCTACCTGGCCATCACCTTTGGCTGGGGGTTTGGGCTCACCATCGGCCTTTTCCTG

L. braziliensis  352 ACTATGGGTGTGTCCGGTGGTCACTTGAATCCGGCTGTAACACTCGCCAACTGCGCCTTT
L. panamensis    352 ACTATGGGTGTGTCCGGTGGTCACTTGAATCCGGCTGTAACACTCGCCAACTGCGCCTTT
L. major         361 AGCATGGCTGTGTCTGGTGGTCACTTGAACCCGGCCGTAACGCTGGCGAACTGTGTCTTT
L. donovani      361 AGCATGGCCGTGTCGGGTGGTCACCTGAACCCAGCTGTGACGCTGGCGAACTGTGTCTTT
L. infantum      361 AGCATGGCCGTGTCGGGTGGTCACCTGAACCCAGCTGTGACGCTGGCGAACTGTGTCTTT
L. tropica       361 AGCATGGCTGTGTCTGGTGGTCACTTGAACCCGGCCGTGACGCTGGCGAACTGTGTCTTT

L. braziliensis  412 GGCGCCTTTCCTTGGCGTAAAGCGCCAGGCTTTATGTTAGCCCAGCTTCTCGGTGCCATC
L. panamensis    412 GGCGCCTTTCCTTGGCGTAAAGCGCCAGGCTTTATGTTAGCCCAGCTTCTCGGTGCCATC
L. major         421 GGTACCTTTCCCTGGGTTAAGCTACCAGGCTATTTTCTAGCCCAGTTTCTCGGAGGTTTT
L. donovani      421 GGTGCCTTTCCCTGGATTAAGCTACCCGGCTATTTTCTCGCCCAATTTCTCGGAGGCTTG
L. infantum      421 GGTGCCTTTCCCTGGATTAAGCTACCCGGCTATTTTCTCGCCCAATTTCTCGGAGGCTTG
L. tropica       421 GGTACCTTTCCCTGGATTAAGCTACCAGGCTATTTTCTCGCCCAGTTTCTCGGAGGCTTT
```

```
L. braziliensis   472 TTGGGTGCGGCCAACGTCTATGGGCTCTTCAAGCAGCACTTTGACGACGCCGGGGTCATG
L. panamensis     472 CTGGGTGCGGCCAACGTCTATGGGCTCTTCAAGCAGCACTTTGACGACGCCGCGGTCATG
L. major          481 GTTGGTGCCGCCAACACCTACGTGCTCTTCAAATCGCACTTTGATGAAGCCGAAAAGAGG
L. donovani       481 GTTGGTGCCGCCAACACCTACGGGCTCTTCAAATCGCACTTTGACGACGCCCAAAAGGCC
L. infantum       481 GTTGGTGCCGCCAACACCTACGGGCTCTTCAAATCGCACTTTGACGACGCCCAAAAGGCC
L. tropica        481 GTTGGTGCCGCCAACACCTACATGCTCTTCAAATCCCACTTTGACGATGCCCAAAAGATG

L. braziliensis   532 TTGCTTCCAAACGAGACGATGGCCTCTAAGTTCAGCGGTGTCTTTGTCACATACCCGAAC
L. panamensis     532 TTGCTTCCAAACGAGACGATGGCCTCTAAGTTCAGCGGTGTCTTTGTCACATACCCGAAC
L. major          541 TTGCTTCTGAATGAAACGATGGCGTCCAAGTACGGCGGAATCTTCGCCACATACCCTAAT
L. donovani       541 TTGCTTCCGAACGAGACGATGGCGTCCAAGTACAGCGGAATCTTCGCCACATACCCTAAT
L. infantum       541 TTGCTTCCGAACGAGACGATGGCGTCCAAGTACAGCGGAATCTTCGCCACATACCCTAAT
L. tropica        541 TTGTCTCCGTCCGAAACGATGGCGTCCAAGTACAGCGGAATCTTCGCCACATACCCTAAT

L. braziliensis   592 GTATCGAACGTCTTTGCGGTGTGGAGTGAGATATTCAACACGATGGTGCTCATGATGGGC
L. panamensis     592 GTCTCGAACGTCTTTGCGGTGTGGAGTGAGATATTCAACACGATGGTGCTCATGATGGGC
L. major          601 GTTGCAAACACCTACGCAGTGTGGAGCGAGGTGTTCAACACCATGGCGCTCATGATGGGC
L. donovani       601 GTTGCAAACACCTACGCAGTGTGGAGTGAGGTGTTCAATACCATGGCGCTCATGATGGGC
L. infantum       601 GTTGCAAACACCTACGCAGTGTGGAGTGAGGTGTTCAATACCATGGCGCTCATGATGGGC
L. tropica        601 GTTGCAAACACCTACGCAGTGTGGAGCGAGGTGTTCAACACCATGGCGCTCATGATGGGC

L. braziliensis   652 ATTCTCGCCATCAACGATAACCGCATGACACCCGCCGATGGCTACAAGCCGGTTGCCGTG
L. panamensis     652 ATTCTCGCCATCAACGATAACCGCATGACACCCGCCGATGGCTACAAGCCGGTTGCCGTG
L. major          661 ATTCTCGCCATCACGGACGCTCGCATGACTCCCGCCGTCGACTACAAGCCGGTGGCTATT
L. donovani       661 ATTCTCGCCATCACGGATCCTCGCATGACTCCCGCCGTCAACTACAAGCCGGTGGCTATT
L. infantum       661 ATTCTCGCCATCACGGATCCTCGCATGACTCCCGCCGTCAACTACAAGCCGGTGGCTATT
L. tropica        661 ATCCTCGCCATCACGGACCCTCGCATGACTCCCGCCGTCAACTACAAGCCGGTGGCTATT

L. braziliensis   712 GGACTGCTGCTTTTCGTCATTGGTATCACGACAGGTATCAACTCTGGATATGCTCTCAAC
L. panamensis     712 GGACTGCTGCTTTTCGTCATTGGTATCACGACCGGTATCAACTCTGGATATGCTCTCAAC
L. major          721 GGACTACTGTTGTTTGTGATTGGCATCGCGTCAGGCATCAACTCTTCCTATGGCCTCAAC
L. donovani       721 GGACTACTGTTGTTTGTCATTGGCATCACGTCAGGCATCAACTCTTCCTATGGCCTCAAC
L. infantum       721 GGACTACTGTTGTTTGTCATTGGCATCACGTCAGGCATCAACTCTTCCTATGGCCTCAAC
L. tropica        721 GGACTACTGTTGTTTGTGATTGGCATCACGTCAGGCATCAACTCTTCCTATGGCCTCAAC

L. braziliensis   772 CCTACACGCGATCTCGGACCCCGTATATTCACAGCCATGCTCTGGGGCAAGGAGCCATTC
L. panamensis     772 CCTACACGCGATCTCGGACCCCGTATATTCACAGCCATGCTCTGGGGCAAGGAGCCATTC
L. major          781 CCCGCACGCGATTTGTCACCTCGCATACTCTCGGCCATGCTCTGGGGCTCGGAGCCTTTC
L. donovani       781 CCCGCACGCGACTTGTCACCTCGCATACTCTCGGCCATGCTCTGGGGCTCAGAGCCTTTC
L. infantum       781 CCCGCACGCGACTTGTCACCTCGCATACTCTCGGCCATGCTCTGGGGCTCAGAGCCTTTC
L. tropica        781 CCCGCACGCGATTTGTCGCCTCGCATACTCTCGGCCATCCTCTGGGGCTCGGAGCCTTTC

L. braziliensis   832 ACCCTGCACGGCTACTACTTCTGGATACCTATCGTCGGTCCGATTGCTGGCGCCCTTCTT
L. panamensis     832 ATCCTGCACGGCTATTACTTCTGGATACCCATCGTCGGTCCGATTGCTGGCGCCCTTCTT
L. major          841 ACGTTGCACAGCTACTACTTTTGGATACCTCTAGTCGTGCCGTTTGTTGGCGCCCTTTTC
L. donovani       841 ACGTTGTACAGCTACTACTTTTGGATACCTCTAGTCGCGCCGTTTGTTGGCGCCCTTCTC
L. infantum       841 ACGTTGTACAGCTACTACTTTTGGATACCTCTAGTCGCGCCGTTTGTTGGCGCCCTTCTC
L. tropica        841 ACGCTGTACAGCCACTACTTTTGGATACCTCTAGTCGCGCCGTTTGTTGGCGCCCTGCTC

L. braziliensis   892 GGCATGTTCTTGTACGTCTTTTGTATAATACCGAGCGGTGCGTAG
L. panamensis     892 GGCATGTTCTTGTACGTCTTTTGTATAATACCGAGCGGTGCGTAG
L. major          901 GGCATGTTCTTGTATGTCTTTTTTATCATTCCGCCCAGCTGTTAG
L. donovani       901 GGCATGTTCTTGTATGTCTTTTTCATCATTCCACCCAACTTCTAG
L. infantum       901 GGCATGTTCTTGTATGTCTTTTTCATCATTCCACCCAACTTCTAG
L. tropica        901 GGCATGTCTGTATGTCTTT--TTTATCATTCCGCCCAGCTGTTAG
```
