# Supplementary material for: Species-Specific Antimonial Sensitivity in Leishmania Is Driven by Post-Transcriptional Regulation of AQP1
Source: PLoS Negl Trop Dis. 2015 Feb 25;9(2):e0003500. doi: 10.1371/journal.pntd.0003500 (PMC4340957; doi:10.1371/journal.pntd.0003500)
Supplement: S3 Fig — The 3’-UTRs from each species were cloned and sequenced as described in the materials and methods. Sequences were aligned using ClustalW2 and Boxshade server. The dashes indicate the gaps introduced to maximize sequence alignment. (PDF) [file pntd.0003500.s003.pdf]

Figure S3

```
L. braziliensis     1 AGCTGCACTGCTG--CTCTAACTTGCTCTGCCATGTATACAACGTTTAGAGCTCGTACGT
L. panamensis       1 AGATGCACTGCTG--TGCTAACTTGCTCTGCCATGCCTACAGCGTTTAGAGCTCGTACGT
L. major            1 GCGTGCTTCGGTACCCCTGCCCTCTCTTTCCTGGGCGTACAAACACCACAGCTCGTGCTT
L. tropica          1 GCGTGCTTCGCTA--CCCCTGCTCTCCTTGCTGGGCGTACAAACTCCACAGCTCACGCTG
L. donovani         1 ACGTGCTTCGCTA--CCCCTGCTCTCTATGCTGGGTGTACAAACACCACAGCTCGTGCTG
L. infantum         1 ACGTGCTTCGCTA--CCCCTGCTCTCTATGCTGGGTGTACAAACACCACAGCTCGTGCTG

L. braziliensis    59 AATGACACGTTATCCTTTGCTTTATTACTACTACCCATACAATATACTGTTTTACACTTG
L. panamensis      59 AATGACACGTTATCCTTTGCTTTATTACTACTACCCATACAATATACTGTTTTACACTTG
L. major           61 GCTAACCATTTAATAT-TCTAAATTCATTATTGTTCTTATTTTACTGCTTTTCCCTTAAG
L. tropica         59 GCTGACCATTTACTTT-TTCCAATGCATTGTTT-TGGGGTATTTTTCTGCTTTCCTTGTG
L. donovani        59 ACTGACCATTTACTCT-TTCCGATTTATTATTATTATTATTTTTCTCTGCTTTCCCTCTG
L. infantum        59 ACTGACCATTTACTCT-TTCCGATTTATTATTATTATTATTTTTCTCTGCTTTCCCTCTG

L. braziliensis   119 ACCTGATGACCCGAGGCACCTCACTGCTGTATTAACATTGAGAACCAACTCTGTGGAGAA
L. panamensis     119 ACCTGATGACCCGAGGCACCTCACTGCTGTATCAACATTGAGAACCCACTCTGTGGAGAA
L. major          120 CCTTGATGACGCAAGCCACCTCACCGTTCTATCAGGGTCCAATGGCCACTCTGCGGGAAC
L. tropica        117 CCTTGATGGCGCGAGCCACCTCACCGCTCTATCAGGGTCCAGTGCCCACTCTGCGGGAAC
L. donovani       118 CCTTTATGACGCGAGCCACCTCACCGTTGTATCAGGGTCCCGTGCCCACTCTGCGGGAAC
L. infantum       118 CCTTTATGACGCGAGCCACCTCACCGTTGTATCAGGGTCCCGTGCCCACTCTGCGGGAAC

L. braziliensis   179 GCCAAGAGCCTGCAGTATTCCCTCGCATACGACTGTGCAAACTCTTGTAACTGGCAAACA
L. panamensis     179 GCCAAGAGCCTGCAGTATTCCCTCACATACGACTGTGCAAACTCTTGTAGCTGGCAAACG
L. major          180 GTCAAAAGCCTGCAGCCTGCCCTCGTGTCGGCGGAGAGGCTGG-----------------
L. tropica        177 GACAAAAGCCTGCAGCCTGCCCTCGTGTCGGCAGACAGGCTTG-----------------
L. donovani       178 GTCGAAAGCCTGCACCCTGCCATCGCCTCGGCGGACAGGCTTG-----------------
L. infantum       178 GTCGAAAGCCTGCACCCTGCCATCGCCTCGGCGGACAGGCTTG-----------------

L. braziliensis   239 TATACAGACAGGTGCTGTACTAGGGAGGCTTACAAGCATCATAAAGCTTGGAGCAGCTAA
L. panamensis     239 TATACAGACAGGTGCTGTGCTAGGGAGGCTTACAAGCATCATAAAGCTTGGAGCAGCTAA
L. major          223 ------AGCTGGCACTGTGCCGGAGAGACTCTCGGCTACGATGCTGGTTCTATCATCTCA
L. tropica        220 ------AGCTGACACTGTACCGGAGAGACTCGCCGCTACAATGCTGTTTCTATCAACCCG
L. donovani       221 ------GGCTGACACTGTACCGGAGACACTCGCGGCTACGATGCTGTTTCTATCATCTCG
L. infantum       221 ------TGCTGACACTGTACCGGAGACACTCGCGGCTACGATGCTGTTTCTATCATCTCG

L. braziliensis   299 CCTCACATCCTGTTCGCAATCTAGCGAATAAACAAACCCTCCTGCTCTTTATTTTCCGCT
L. panamensis     299 CCTCACATCCTGTTCGCAATCTAGCGAATAAACAAACCCTCCTGCTCTTTATTTTCTGCT
L. major          277 CTGCCAGTCGTTTCAGCGACTCGGCAAGCATG---CGCTTCCACTCAC----TGTCTGCT
L. tropica        274 CTGCCAGTCGCTTCAGCGACTCAGCAAGCATG---CGCTTCCACTCACTGCTTTTCTGCT
L. donovani       275 CTGCCAGTCGTTTCGACGACTCAGCAAGCATG---CGCTTCCAATCATTGCCCTTCTGCT
L. infantum       275 CTGCCAGTCGTTTCGACGACTCAGCAAGCATG---CGCTTCCAATCATTGCCCTTCTGCT

L. braziliensis   359 GTTGCTGCGGCGTGTGAGTGTTGGGCTTAGTCCAGGAGTGATCCTGGGCTATGTGCAGTA
L. panamensis     359 GTTGCTGCAGCGTGTTAGTGTTGGGCTTAGTCCTGGAGTGATCCTGGGCTATGTGCAGTA
L. major          330 TTTGCAGCGCTTCGGTAATACCGAGCGTGATCCCGCGCCTAGCCTGCGCTGTATGCAGCT
L. tropica        331 TTTGCGGCGCTTCGGTAATACCGAGCGTGATCCTGGACCTAGCCTGCGCTGTATGCAGCT
L. donovani       332 TTTGCGGCGCTTCGGTAATACCGAGCGTGATTCTGGACCTGGCCTGCGCTGTATGCAGCG
L. infantum       332 TTTGCGGCGCTTCGGTAATACCGAGTGTGATTCTGGACCTGGCCTGCGCTGTATGCAGCG

L. braziliensis   419 GAACTGCAGCTGTTTCGACGATCTCCGACAGTATTGACAGCCCCTATTGCTTGAAGTAGC
L. panamensis     419 GAACTGCAGCTATCTCAACGATCTCCAACAGTATTGACAGCCCCTATTGCTTGAAGTAGC
L. major          390 CAATCNCCGATCGCCGCACCACACGAAACGGTGCTGGCCAGCCCGGCTCAATG-------
L. tropica        391 GAATCACCGATTGCTGCACCACACGAAACGGTGCTGGTCAGCCCGGCTCGATACAGCCGT
L. donovani       392 GAATCACGGTTTGCTGCACCACACGAAAGGGTGCTAGTCATCCCGTCTCGATACTGCCGT
L. infantum       392 GAATCACGGTTTTCTGCACCACACGAAAGGGTGCTAGTCATCCCGTCTCGATACTGCCGT
```

```
L. braziliensis   479 AACAAGGTAGCCGTTGTGGGGAGAACTGTGGTTGTACGTGTCTATGGGTGCCCACGTGCT
L. panamensis     479 AACAAGGTAGCCGATGTGGGGAGAACTGTGATTGTGCGCGTCTATGGGTGCCCACGTGCT
L. major          443 -------CAGCAACGGTA-GGTGAAGTGCTATTTTAAGGGTGAGC--CTGTCGACTTTTT
L. tropica        451 CGT---GCAGCAGCGGTA-GGTGAACTGCTATTTTAAGGGTGAGC--TTGTTGACTTTTT
L. donovani       452 CGT---GCAGCAGCCGTA-GGTGACCTGCTATTTTAAGGGTCAGT--TTGTTGACTTTCT
L. infantum       452 CGT---GCAGCAGCCGTA-G-TGACCTGCTATTTTAAGGGTCAGT--TTGTTGACTTTCT

L. braziliensis   539 CGCCATGGAATAGACACGTTCC------------CCCCCAAAATGCTTTCTCGTTGTCCA
L. panamensis     539 CGCCATGGAGTAGACACGTTCCTTCCCCCCCCCCCCCCCCAAATGCTTTCTCTTTGTCCA
L. major          493 TGCGATAGA---CGGACGCGCATTCCCCCCCCCCAACCCAAAAAAAAACGCTCTCTCTC
L. tropica        505 CGCGGTAGA---TGCACACGCGTTGCCAAAACAACAAAGAAACGCGCGCTCTCTCTCTG
L. donovani       506 TGCGAGAGA---TGAACACGCGTTGCCAAATAAAAAAAGAGCATGCTCTCTCTCTCTCTC
L. infantum       505 TGCGAGAGA---TGAACACGCGTTGCCAAATAAAAAAAGAGCATGCTCTCTCTCTCTCTC

L. braziliensis   587 --------GCTCCCTTGTTCGT---CTTGTTTTATTTTTTATTTTATTGTGCTACCCCTT
L. panamensis     599 --------GCTCCCTTGTTCGT---CTTGTTTTATTTTTTATTTTATTTTGCTACCCCTT
L. major          550 TCTGTTTTGCTTTCNTGCGCTTTGTGTTGCGCTCTTTATCATCTGC--TTTTTAGT----
L. tropica        562 --TGTTGTGCTCCCTTTGCGTTTGTCTTGCGTTCTTATCCTCTTCTTTTTTCAGTC----
L. donovani       563 TCTGCTGTGGTCCATTTGCGTTTATGCTGCTTTCTTTACCATTTTC--TTCTCAGC----
L. infantum       562 TCTGCTGTGGTCCATTTGCGTTTATGCTGCTTTCTTTACCATTTCT--TCTCAGCC----

L. braziliensis   636 AGCACTGCTTCTGCCAGTTATTTGGTTGCTGTACGGGATTTTGCGCAG-GGGCTGCTATT
L. panamensis     648 AGCACTGCTTCTGCCAGTTATTTGGTTGCTGTACGGGATTTTGCGCAGGGGGCTGCTATT
L. major          604 -----CCAATGGTGCTCTATTTT-T-----------------GTGCAGAAGGGTAAACTT
L. tropica        616 -----TGGGTGGTGCTCTCTTTT-G-----------------GTGCAGATGGGTGAAACT
L. donovani       617 -----CTGCTGGTGCTCTATTTT-T-----------------GTGCAGAAGGGTGAACTT
L. infantum       616 -----TGCTGGTGCTTCTATTTT-T-----------------GTGCAGAAGGGTGAACTT

L. braziliensis   695 GTCCTCG---GCAGGG-GGGAGCATCATCGCAGTCAAAGACAGACTTGTT--AACCAGAT
L. panamensis     708 GTCCTCG---GCAGGG-GGGAGCATCATCACAGTCAAAGACAAACTTGTT--AACCAGAT
L. major          641 GTCGCCGG--TAAGGGAG----CAACATGGTGGCCAACGATGACGTTGTC--AAACCTGT
L. tropica        653 TGTTCGCTCGTTAAGGGGGGCGACATGGGTCCGCCAACGATGACGCTCTTCAAAGCTGGT
L. donovani       654 TTCGCCCG--TAAGGGAG--CGACAT--GGTAGCCAACGACAACTTTCTA--CAGCCTGT
L. infantum       653 TTCGCCCG--TAAGGGAG--CGACAT--GGTAGCCAACGACAACTTTCTA--CAGCCTGT

L. braziliensis   749 GTTGTCTCTTCTG---CCCCGCGCTGCTGCAAAGGTGT-- G GAAGC GGCCAC-----
L. panamensis     762 GTTGTCTCTTCTG---CTCCGCGCTGCTGCAAAGGTGT--GGAGAAGCAGGCCAC-----
L. major          693 GTGATGTTTGCTTCG-CCTCGTACTGCTGCACTATCAA--GGATACGAAGGCGAATGGGT
L. tropica        713 GTGATGTTTGCTCTTGCTTCGTGCTGCTGCCACTTATCAGGAATTACGAAAGGCTACCTC
L. donovani       706 GTGATGTCTGCTCTG-CTTCGTGCTGCTGCACTCTCAA--GGATACGGAGGCTATTCGGC
L. infantum       705 GTGATGTCTGCTCTG-CTTCGTGCTGCTGCACTCTCAA--GGATACGGAGGCTATTCGGC

L. braziliensis   799 -TCTGGATATCTCTGTA-----TTTGTTCCCTACGTGATGCGCG--TGATAAGATATATG
L. panamensis     812 -TCTGGATATCTCTGTA-----TTTGTTCCCTACGTGATGTGCG--TGATAAGATATATG
L. major          750 CTATCTACACATTTGCGTCGGCGTCGTGTGCGCAGTGAAGTGAGTGTGTGTGTGTGTGTG
L. tropica        773 CGGTTCAATCTACACCATTGGCGTCCGGGTCTGTGTGCACCCGTGGAAAAGTTGTGGG--
L. donovani       763 CTATCTACACATTTGCTTTGGCGTCGGGTGCACAGTGAAGTTTGTGTGCGTGTGTGTG--
L. infantum       762 CTATCTACACATTTGCTTTGGCGTCGGGTGCACAGTGAAGTTTGTGTGCGTGTGTGTG--

L. braziliensis   851 TTTGCTTTCAAGGATCACCGCGCATAACTGTCCACAG-AGTGTGCGGGCCCTGTGC----
L. panamensis     864 TTTGCTTTCAAGGATCACCGCGCATAACTGTCCATAG-AGTGTGCGGGCCCTGTGC----
L. major          810 TGTGCTGCGAACGATCGATGCGCAT--GACTTTCAAAAGGTGTGCAGGTCTCATCCACAG
L. tropica        831 --TT------------------GTGGACTGCAAAAGAGTGTGTGAGCTTCGGTCCCACAG
L. donovani       821 --TGCTGTCAGCAATCAATACGCATACCTTTCAAAAAAGGTGTGCTGGCCTCGTCCACAG
L. infantum       820 --TGCTGTCAGCAATCAATACGCATACCTTTCAAAAAAGGTGTGCTGGCCTCGTCCACAG
```

```
L. braziliensis  906 ---------------------------------CGAGCATCTTCATTGGCGTTCTCTGTT
L. panamensis    919 ---------------------------------CGAGCATCTTCATTGACGTTCTCTGT-
L. major         868 CATCGTTTTCGTCCTAGGTGGCGTCTTCTTCGACAATTCGATGCGTTGGCTATTGCCTGT
L. tropica       871 GCATCGTTTCCTCGTCGATTG-CGTCTTTCCGTGGATCCATTGCGTTGCTATTGGCTGTT
L. donovani      879 CACCGTTTTCCTCCTACGTGGCGTATTCTCCGAGGATTCAATGCGTTGGCTTTTGCCTGT
L. infantum      878 CACCGTTTTCCTCCTACGTGGCGTATTCTCCGAGGATTCAATGCGTTGGCTTTTGCCTGT

L. braziliensis  933 TTGTCGTGACGTTCTTCTCCCAC--ATTCCACGTGGCGGTTACAACAACGA--TCTTACA
L. panamensis    945 ----CGTGACATCCTTCTCCCAC--ATTCCACGTGGCGGGTACAACAACGA--TCTTACA
L. major         928 TGCCGTGAAAGTATATAGTAGCTGTACCGCATGTCTGTGGTTCGGCTACCA--CCGTATA
L. tropica       930 TTCGTGAAAGTATCTTAGTAGCTGTACTGCGTGCCTTTGGTGCAGCTAACCACCGTAATA
L. donovani      939 GTCCGCGAAAGTATATATTGGCTGTAGTGCATGTCTTTGGTGCGACTACCG--CCGTATA
L. infantum      938 GTCCGCGAAAGTATATATTGGCTGTAGTGCATGTCTTTGGTGCGACTACCG--CCGTATA

L. braziliensis  989 CTGTTGCACGTTGGCATTTTGAGGAGGTTTCGCGTAAAACCATACGA--GTGTACCCGCA
L. panamensis    997 CTGTTGCACGTTGGCATTTTGAGGAGGTTTCGCGTAAAACCATACGA--GTGTACCCGCA
L. major         986 CTGCTATGTTTTGACATTTTGAGGAGTTCTCTCTCGCGAAAACAAA---GCGAAAAAGTG
L. tropica       990 TGGCTATGTTTTGACATTTGAGGAGCTTCTCTCTCGCGAAAGAAAAAGGCGAAACAAGTG
L. donovani      997 CTGCTATGCTTTGACATTTTGAGGAGCTCCCTCGAGGAAAAAGAAGCGAAAAACAAAACG
L. infantum      996 CTGCTATGCTTTGACATTTTGAGGAGCTCCCTCGAGGAAAAAGAAGCGAAAAACAAAACG

L. braziliensis 1047 ---------GCTCTGTGGACGCTTGCCCAGAACAGCCAACAAGAAATGAGGAATCCAAGA
L. panamensis   1055 ---------GCTCTGTGGACGCTTGCCCAGAACAGCCAACAAAAAATGAGGAATCCAAGA
L. major        1043 CCGTTAAGAACTCTCATGTCCCTTTGAGAAGATTGATCAGTGACAACGACGAGTCTAGCA
L. tropica      1050 CCGTTAAGAACTCTCGTGACCCTTTGAGCAGATTGGGCAGTGAAAACGACAAGTCTAGCA
L. donovani     1057 CCGTTAAGAACTCCCGTGACCGTTTGAGCAGACTGGGCAGTGAAAACGACGAGTCTGGCA
L. infantum     1056 CCGTTAAGAACTCCCGTGACCGTTTGAGCAGACTGGGCAGTGAAAACGACGAGTCTGGCA

L. braziliensis 1098 CATTGTCTTTTCTTGCCTGATTGTTTTGATGGCTTTTTTGTCGAGGTGTGTGTGTCTTTC
L. panamensis   1106 CATGGTCTTTTCTTGCCTGAATGTTTCGATGGCTTTTTTGTCGAGGTGTGTGTGTCTTTC
L. major        1103 AATGGACTTTCCTTTTTGTT--TCAGCCTTGCTCATATGTGTGATGTTC------CTACG
L. tropica      1110 AATAGACTTTCCTCTTTTTATTTCAGCCTTGCTCATCTCTATGATGTTC------CTACG
L. donovani     1117 AATGGGCTTTCCTTTTTTTTTC------ATGCTCACCTGTATGATTTTC------CTCCG
L. infantum     1116 AATGGGCTTTCCTTTTTTTTTC------ATGCTCACCTGTATGATTTTC------CTCCG

L. braziliensis 1158 TGCAGATTCCCGCCTTTTTTTTTTCATAACACTGCCGCGTGTTTGTGAGCGACCGCAGTG
L. panamensis   1166 TGCAGTTTCCCGCCTTT-TTTTTTCATAACACTGCCGCGTGTTTGTGAGCGACCGCAGTG
L. major        1155 TGCTTATCCTTTGAAGTTTCTTTTCTCAGCGCT-------CCTTTCGCTCTTGCGGCTGA
L. tropica      1164 TGCTTATCTGTTGAAGTTTTTCTCTTCTCTTTCAGCGCTCTTTTCTCTCTTTGCTGCTGA
L. donovani     1165 TGCTTATCTTTTGAATTTTCTCTCTTCAGCTCT-------CTTTTTGCTCTTGCTGCTGA
L. infantum     1164 TGCTTATCTTTTGAATTTTCTCTCTTCAGCTCT-------CTTTTTGCTCTTGCTGCTGA

L. braziliensis 1218 TAGAACTGTCTTAAAAAGAAAATGCAAAACGTTTTTTCTCCTATTTTTTTGTTTTTTTTT
L. panamensis   1225 TAGAACCGTCTTAAAAAGAAAATGCAAAACGTTTTTTCTCCTATTTTTTTGCTTTT----
L. major        1208 TAGCACTGAACAAAAC-GTTATTGCCGGGAAACCTTTC-CTTTCTGCGT-----------
L. tropica      1224 TAGTACTGAATTAAAC-GTCATTGCTGCAAAATCTTTTTCTTTCTGTGT-----------
L. donovani     1218 TAGCGATGGACGAAAA-GTTGTTGCTGCAAAATCTTTC-CTTTCTGCGT-----------
L. infantum     1217 TAGCGATGGACGAAAA-GTTGTTGCTGCAAAATCTTTC-CTTTCTGCGT-----------

L. braziliensis 1278 TGCTGAAACCAAAATGAAGTTTGTCTCTGCGGCTT--TTTTTCCTCCACTCTTTT-----
L. panamensis   1281 -GCTCAAACCAAAATGAAGTTTGTCTCTGCGTCTTTTTTTTTTCTCCACTCTGTT-----
L. major        1255 -TGTTTTAATATATATATATATATGTCTGTATCTTTTTTGTGTCTCACCCCTCTTCTTTC
L. tropica      1272 -TGTTCCAAGATGATTATATATGACTCTT----------TTTTTGTCTCACCCTTCTTTG
L. donovani     1265 -TGCTTCAAGATGATCACATATATGACTC------ATTTTTGTTTCACATCTCTTCTTTC
L. infantum     1264 -TGCTTCAAGATGATCACATATATGACTC------ATTTTTGTTTCACATCTCTTCTTTC
```

```
L. braziliensis 1331 ------------------------CGTGCCATGTTTGCTTGAAAATGTGCTGTTTC--CC
L. panamensis   1335 ------------------------TGTGCCATGTTTGCTTGAAAATGTGCTGTTTCCCTC
L. major        1314 CACTCTTTCTCCTTGTTCCCC-------CTTTTTTCGTTACGGAAAGCGAACTGTCTAAC
L. tropica      1321 CACCCTCTCTCCTTGCTAC-CCCTTTTTTTCTTTACTGAAAGCGAACTTTTGTTTCTAAC
L. donovani     1318 CACCTTTTCTCCTTGCTACCCTTTCTTTCTCTTTACTGAAAGCGAACCTTTTTTTCTACC
L. infantum     1317 CACCTTTTCTCCTTGCTACCCTTTCTTTCTCTTTACTGAAAGCGAACCTTTTTTTCTACC

L. braziliensis 1365 CCTTTTCGGTGCTTCTATTCAATGCTTAAGATGAAAGGAAGTCAAGGCGACGAATTGTAT
L. panamensis   1371 CCTTTTCGGTGCTTCTATTCAATGCTTAAGATAAAAGGAAGTCAAGGCGACGAATTCTAT
L. major        1367 GCTTTGCATTTCTTGGTTGAATGCCAGATTTCACGCGAAGCTGAGAAGGTGAACCTAGT
L. tropica      1380 GCTTTGCATTTCTTCGGTTGACTGCCGGATTTCAAGAGAAGCTGAGAAGGTGAACCGTGT
L. donovani     1378 TCTTAGCATTTCTTCGGTTGAATGCCCGAGTTCACGAAAAGCTGTGAAGGTGAACCGTGT
L. infantum     1377 TCTTAGCATTTCTTCGGTTGAATGCCCGAGTTCACGAAAAGCTGTGAAGGTGAACCGTGT

L. braziliensis 1425 TATCAACCGTCTACTCTTCCCACTATCTCTGTCCTATTCTACGGCGCACTTCGTACGCAA
L. panamensis   1431 TATCAACCGTCTACTCTTCCCACTATCTCTCTCCTGTTCTACGGCGCACTTCGTACGCAA
L. major        1427 TATCAGCAGCGTACTCTTCCTACTACCTCTATCACTTTCTTCTAACACTTCCACACAAAC
L. tropica      1440 TGCCAGCAGTGTACTCTTCCCACTACCTCTATCACTTTCTTGTAACACTTCCACACAGAC
L. donovani     1438 TATCAGCAGCGTATTCTTCCCACTACCTCTATCACTTTCTTCTAACACTTCCGCACACAC
L. infantum     1437 TATCAGCAGCGTATTCTTCCCACTACCTCTATCACTTTCTTCTAACACTTCCGCACAGAC

L. braziliensis 1485 CCCA-----------CCCACCCACCCACCCACAATG--CCACTGCAAAATGGGAACTCAG
L. panamensis   1491 CCCA-----------CCC--------ACCCACAATG--CCACTGCAAACTGGGAACTCAG
L. major        1487 ACACA------------CACACACACACACACCATGTTGCTGTGCGAATGCGAACCTGAG
L. tropica      1500 ACAGA--------------CACACACACACACCATGTCGCTGTGCGAATGCGAACCTGAG
L. donovani     1498 A--------------CACACACATAAACACACAATTTCGGTGTGCGAATGCAAACCTGAG
L. infantum     1497 ACACACAGACACACACACACACATAAACACACAATTTCGGTGTGCGAATGCAAACCTGAG

L. braziliensis 1532 GAGTTGTGAAGAGCGGATCCTACAGCTCAGAGTACCCTG-CGCCGAAACAGCGAGT----
L. panamensis   1530 GGGTTGTGAAGAGCGGATCCTACAGCTCGGAGTACACTG-CGCCGAAACAGTGAGT----
L. major        1535 TGGGTGCAAAGTGTGATTCCCATAACGATGAGCGTCACTTCACACTTACATTTTCTTGTT
L. tropica      1546 TGGCTGCAAAGCGTGATTCCCATAACGATGAGCGTCACGGCACGTTTACATTTTCTTGAT
L. donovani     1544 CGAGTGCAAAGCGTGATTCCCATAACTATGAGCGTCACTGCGCATTTACATTTTATTGTT
L. infantum     1557 CGAGTGCAAAGCGTGATTCCCATAACTATGAGCGTCACTGCGCATTTACATTTTATTGTT

L. braziliensis 1587 -------TTTTTTGTTGTTGTTTTCTCAGCGGCTAGGATGCGAAAGACTCAGTGCCCACG
L. panamensis   1585 -------TT-TTTGTTGTTGTTTTCTCAGCGGCTAGGATGCGAAAGACTCAGTGCCCACG
L. major        1595 TTCTTTTCCTTTTGTTTTGTCACTTTCCGGCATACCATGGGAAAAAGGTGAGTGCACACG
L. tropica      1606 TGCTTTCCTTTCCTTTTCAC---TCTCCG-GCATATCGTGCGAAAAGATGAGTGCACACG
L. donovani     1604 TGCTTTTCCTTTTTTTTAAC---TCTGCG-GCATGTCATGCGAAAAGATGAGTGCACATG
L. infantum     1617 TGCTTTTCCTTTTTTTAAAC---TCTGCG-GCATGTCATGCGAAAAGATGAGTGCACATG

L. braziliensis 1640 AGATTCTTCTTGCAGGAGGCCTGGTTTGCCTCTCAGGCGCCAGGCAAGTGAGTATCGTGG
L. panamensis   1637 AGATTCTTCTTGCAGGGGGCCTGGTTTGCCTCTCAGGCGCCAGGTAAGTGAGTATCGTGG
L. major        1655 GGGCGCTTCTTGCGTGAGGCTTGGCTTGCCTTGCACGCACTAGGTGACCCAGCGTGACGG
L. tropica      1662 GCGTACTTCTTGCGTGAGGCTTGGCTTGCCTTGCACGCGCTAGGTGACCCAGCGTGGCGG
L. donovani     1660 GCGTGCTTCCTGCGTGAGGCTTGGCTTGCCTTGCACGCGCAAGGTGACCCAGCGTCGCGG
L. infantum     1673 GCGTGCTTCCTGCGTGAGGCTTGGCTTGCCTCGCACGCGCAAGGTGACCCAGCGTCGCGG

L. braziliensis 1700 TATCAACTGGATGCTGATGCGGACTCAAAGAGAAACGATATACTTTCCATAGGTTTCCGT
L. panamensis   1697 TATTAACGGGAGGCTGATGCGGACTCAAAGAGAAGCGATTTACTTTCCATAGGTTTCCGT
L. major        1715 CAGGCAATGGATGCTGATGCTGTTTGAAAAAGAGGCAGGTGAAGTGCTATAAGTTTTCGT
L. tropica      1722 TAGGACATAGACGCCGATGCTGTCTGAAAAAGAAGCAGGTGAAGTTCTATAGGGTTTCGT
L. donovani     1720 TAGGAAATGGATGCTGATGTCGTCTGAAAAAGAAGCAGGCGAAGTGCTATAGGGTTTCGT
L. infantum     1733 TAGGAAATGGATGCTGATGCCGTCTGAAAAAGAAGCAGGCGAAGTGCTATAGGGTTTCGT
```

```
L. braziliensis 1760 CGCTACTGACGTCCCAGCGGCGAAAAAAGAGCACGCTGTCAGCATAAG-----
L. panamensis   1757 CGCTACTGACGTCCCAGCGGCGAAAAAAAAGCACGCTGTCAGCGTAAGGTAAG
L. major        1775 CGCAAGGAACTTTCTCGTGGCAAACGAACGGCAGCAAAAAAGGGAG-------
L. tropica      1782 CGCTAAGGGCTTTCCAGTGGC--------------------------------
L. donovani     1780 CGCTAAAAGCCTTCCGGTGGCAAACAAACCGCAAAAAAAAGAGAG--------
L. infantum     1793 CGCTAAAAGCCTTCCGGTGGCAAACAAACCGCAAAAAAAAGAGAG--------
```
