# Supplementary material for: Species-Specific Antimonial Sensitivity in Leishmania Is Driven by Post-Transcriptional Regulation of AQP1
Source: PLoS Negl Trop Dis. 2015 Feb 25;9(2):e0003500. doi: 10.1371/journal.pntd.0003500 (PMC4340957; doi:10.1371/journal.pntd.0003500)
Supplement: S4 Fig — The 3’-UTRs from each species were cloned and sequenced as described in the materials and methods. Sequences were aligned using ClustalW2 and Boxshade server. The dashes indicate the gaps introduced to maximize sequence alignment. (PDF) [file pntd.0003500.s004.pdf]

Figure S4

```
L. donovani    1 ACGTGCTTCGCTACCCCTGCTCTCTATGCTGGGTGTACAAACACCACAGCTCGTGCTGAC
L. infantum    1 ACGTGCTTCGCTACCCCTGCTCTCTATGCTGGGTGTACAAACACCACAGCTCGTGCTGAC

L. donovani   61 TGACCATTTACTCTTTCCGATTTATTATTATTATTATTTTTCTCTGCTTTCCCTCTGCCT
L. infantum   61 TGACCATTTACTCTTTCCGATTTATTATTATTATTATTTTTCTCTGCTTTCCCTCTGCCT

L. donovani  121 TTATGACGCGAGCCACCTCACCGTTGTATCAGGGTCCCGTGCCCACTCTGCGGGAACGTC
L. infantum  121 TTATGACGCGAGCCACCTCACCGTTGTATCAGGGTCCCGTGCCCACTCTGCGGGAACGTC

L. donovani  181 GAAAGCCTGCACCCTGCCATCGCCTCGGCGGACAGGCTTGGGCTGACACTGTACCGGAGA
L. infantum  181 GAAAGCCTGCACCCTGCCATCGCCTCGGCGGACAGGCTTGTGCTGACACTGTACCGGAGA

L. donovani  241 CACTCGCGGCTACGATGCTGTTTCTATCATCTCGCTGCCAGTCGTTTCGACGACTCAGCA
L. infantum  241 CACTCGCGGCTACGATGCTGTTTCTATCATCTCGCTGCCAGTCGTTTCGACGACTCAGCA

L. donovani  301 AGCATGCGCTTCCAATCATTGCCCTTCTGCTTTTGCGGCGCTTCGGTAATACCGAGCGTG
L. infantum  301 AGCATGCGCTTCCAATCATTGCCCTTCTGCTTTTGCGGCGCTTCGGTAATACCGAGTGTG

L. donovani  361 ATTCTGGACCTGGCCTGCGCTGTATGCAGCGGAATCACGGTTTGCTGCACCACACGAAAG
L. infantum  361 ATTCTGGACCTGGCCTGCGCTGTATGCAGCGGAATCACGGTTTTCTGCACCACACGAAAG

L. donovani  421 GGTGCTAGTCATCCCGTCTCGATACTGCCGTCGTGCAGCAGCCGTAGGTGACCTGCTATT
L. infantum  421 GGTGCTAGTCATCCCGTCTCGATACTGCCGTCGTGCAGCAGCCGTAG-TGACCTGCTATT

L. donovani  481 TTAAGGGTCAGTTTGTTGACTTTCTTGCGAGAGATGAACACGCGTTGCCAAATAAAAAAA
L. infantum  480 TTAAGGGTCAGTTTGTTGACTTTCTTGCGAGAGATGAACACGCGTTGCCAAATAAAAAAA

L. donovani  541 GAGCATGCTCTCTCTCTCTCTCTCTGCTGTGGTCCATTTGCGTTTATGCTGCTTTCTTTA
L. infantum  540 GAGCATGCTCTCTCTCTCTCTCTCTGCTGTGGTCCATTTGCGTTTATGCTGCTTTCTTTA

L. donovani  601 CCATTTTCTTCTCAGCCTGCTGGTGCT-CTATTTTTGTGCAGAAGGGTGAACTTTTCGCC
L. infantum  600 CCATTT-CTTCTCAGCCTGCTGGTGCTTCTATTTTTGTGCAGAAGGGTGAACTTTTCGCC

L. donovani  660 CGTAAGGGAGCGACATGGTAGCCAACGACAACTTTCTACAGCCTGTGTGATGTCTGCTCT
L. infantum  659 CGTAAGGGAGCGACATGGTAGCCAACGACAACTTTCTACAGCCTGTGTGATGTCTGCTCT

L. donovani  720 GCTTCGTGCTGCTGCACTCTCAAGGATACGGAGGCTATTCGGCCTATCTACACATTTGCT
L. infantum  719 GCTTCGTGCTGCTGCACTCTCAAGGATACGGAGGCTATTCGGCCTATCTACACATTTGCT

L. donovani  780 TTGGCGTCGGGTGCACAGTGAAGTTTGTGTGCGTGTGTGTGTGCTGTCAGCAATCAATAC
L. infantum  779 TTGGCGTCGGGTGCACAGTGAAGTTTGTGTGCGTGTGTGTGTGCTGTCAGCAATCAATAC

L. donovani  840 GCATACCTTTCAAAAAAGGTGTGCTGGCCTCGTCCACAGCACCGTTTTCCTCCTACGTGG
L. infantum  839 GCATACCTTTCAAAAAAGGTGTGCTGGCCTCGTCCACAGCACCGTTTTCCTCCTACGTGG

L. donovani  900 CGTATTCTCCGAGGATTCAATGCGTTGGCTTTTGCCTGTGTCCGCGAAAGTATATATTGG
L. infantum  899 CGTATTCTCCGAGGATTCAATGCGTTGGCTTTTGCCTGTGTCCGCGAAAGTATATATTGG
```

```
L. donovani  960 CTGTAGTGCATGTCTTTGGTGCGACTACCGCCGTATACTGCTATGCTTTGACATTTTGAG
L. infantum  959 CTGTAGTGCATGTCTTTGGTGCGACTACCGCCGTATACTGCTATGCTTTGACATTTTGAG

L. donovani 1020 GAGCTCCCTCGAGGAAAAAGAAGCGAAAAACAAAACGCCGTTAAGAACTCCCGTGACCGT
L. infantum 1019 GAGCTCCCTCGAGGAAAAAGAAGCGAAAAACAAAACGCCGTTAAGAACTCCCGTGACCGT

L. donovani 1080 TTGAGCAGACTGGGCAGTGAAAACGACGAGTCTGGCAAATGGGCTTTCCTTTTTTTTTCA
L. infantum 1079 TTGAGCAGACTGGGCAGTGAAAACGACGAGTCTGGCAAATGGGCTTTCCTTTTTTTTTCA

L. donovani 1140 TGCTCACCTGTATGATTTTCCTCCGTGCTTATCTTTTGAATTTTCTCTCTTCAGCTCTCT
L. infantum 1139 TGCTCACCTGTATGATTTTCCTCCGTGCTTATCTTTTGAATTTTCTCTCTTCAGCTCTCT

L. donovani 1200 TTTTGCTCTTGCTGCTGATAGCGATGGACGAAAAGTTGTTGCTGCAAAATCTTTCCTTTC
L. infantum 1199 TTTTGCTCTTGCTGCTGATAGCGATGGACGAAAAGTTGTTGCTGCAAAATCTTTCCTTTC

L. donovani 1260 TGCGTTGCTTCAAGATGATCACATATATGACTCATTTTTGTTTCACATCTCTTCTTTCCA
L. infantum 1259 TGCGTTGCTTCAAGATGATCACATATATGACTCATTTTTGTTTCACATCTCTTCTTTCCA

L. donovani 1320 CCTTTTCTCCTTGCTACCCTTTCTTTCTCTTTACTGAAAGCGAACCTTTTTTTCTACCTC
L. infantum 1319 CCTTTTCTCCTTGCTACCCTTTCTTTCTCTTTACTGAAAGCGAACCTTTTTTTCTACCTC

L. donovani 1380 TTAGCATTTCTTCGGTTGAATGCCCGAGTTCACGAAAAGCTGTGAAGGTGAACCGTGTTA
L. infantum 1379 TTAGCATTTCTTCGGTTGAATGCCCGAGTTCACGAAAAGCTGTGAAGGTGAACCGTGTTA

L. donovani 1440 TCAGCAGCGTATTCTTCCCACTACCTCTATCACTTTCTTCTAACACTTCCGCACA-----
L. infantum 1439 TCAGCAGCGTATTCTTCCCACTACCTCTATCACTTTCTTCTAACACTTCCGCACAGACAC

L. donovani 1495 ---------CACACACACACATAAACACACAATTTCGGTGTGCGAATGCAAACCTGAGCG
L. infantum 1499 ACACAGACACACACACACACATAAACACACAATTTCGGTGTGCGAATGCAAACCTGAGCG

L. donovani 1546 AGTGCAAAGCGTGATTCCCATAACTATGAGCGTCACTGCGCATTTACATTTTATTGTTTG
L. infantum 1559 AGTGCAAAGCGTGATTCCCATAACTATGAGCGTCACTGCGCATTTACATTTTATTGTTTG

L. donovani 1606 CTTTTCCTTTTTTTTAACTCTGCGGCATGTCATGCGAAAAGATGAGTGCACATGGCGTGC
L. infantum 1619 CTTTTCCTTTTTTTAAACTCTGCGGCATGTCATGCGAAAAGATGAGTGCACATGGCGTGC

L. donovani 1666 TTCCTGCGTGAGGCTTGGCTTGCCTTGCACGCGCAAGGTGACCCAGCGTCGCGGTAGGAA
L. infantum 1679 TTCCTGCGTGAGGCTTGGCTTGCCTCGCACGCGCAAGGTGACCCAGCGTCGCGGTAGGAA

L. donovani 1726 ATGGATGCTGATGTCGTCTGAAAAAGAAGCAGGCGAAGTGCTATAGGGTTTCGTCGCTAA
L. infantum 1739 ATGGATGCTGATGCCGTCTGAAAAAGAAGCAGGCGAAGTGCTATAGGGTTTCGTCGCTAA

L. donovani 1786 AAGCCTTCCGGTGGCAAACAAACCGCAAAAAAAAGAGAG
L. infantum 1799 AAGCCTTCCGGTGGCAAACAAACCGCAAAAAAAAGAGAG
```
