# Supplementary material for: Species-Specific Antimonial Sensitivity in Leishmania Is Driven by Post-Transcriptional Regulation of AQP1
Source: PLoS Negl Trop Dis. 2015 Feb 25;9(2):e0003500. doi: 10.1371/journal.pntd.0003500 (PMC4340957; doi:10.1371/journal.pntd.0003500)
Supplement: S5 Fig — The 3’-UTRs from each species were cloned and sequenced as described in the materials and methods. Sequences were aligned using ClustalW2 and Boxshade server. The dashes indicate the gaps introduced to maximize sequence alignment. (PDF) [file pntd.0003500.s005.pdf]

Figure S5

```
L. major      1 GCGTGCTTCGGTACCCCTGCCCTCTCTTTCCTGGGCGTACAAACACCACAGCTCGTGCTT
L. tropica    1 GCGTGCTTCGCTACC--CCTGCTCTCCTTGCTGGGCGTACAAACTCCACAGCTCACGCTG

L. major     61 GCTAACCATTTAATATTCTAAATTCATTATTGTTCTTATTTTACTGCTTTTCCCTTAAGC
L. tropica   59 GCTGACCATTTACTTTTTCCAATGCATTGTTTTGG-GGTATTTTTCTGCTTTCCTTGTGC

L. major    121 CTTGATGACGCAAGCCACCTCACCGTTCTATCAGGGTCCAATGGCCACTCTGCGGGAACG
L. tropica  118 CTTGATGGCGCGAGCCACCTCACCGCTCTATCAGGGTCCAGTGCCCACTCTGCGGGAACG

L. major    181 TCAAAAGCCTGCAGCCTGCCCTCGTGTCGGCGGAGAGGCTGGAGCTGGCACTGTGCCGGA
L. tropica  178 ACAAAAGCCTGCAGCCTGCCCTCGTGTCGGCAGACAGGCTTGAGCTGACACTGTACCGGA

L. major    241 GAGACTCTCGGCTACGATGCTGGTTCTATCATCTCACTGCCAGTCGTTTCAGCGACTCGG
L. tropica  238 GAGACTCGCCGCTACAATGCTGTTTCTATCAACCCGCTGCCAGTCGCTTCAGCGACTCAG

L. major    301 CAAGCATGCGCTTCCACTCACT----GTCTGCTTTTGCAGCGCTTCGGTAATACCGAGCG
L. tropica  298 CAAGCATGCGCTTCCACTCACTGCTTTTCTGCTTTTGCGGCGCTTCGGTAATACCGAGCG

L. major    357 TGATCCCGCGCCTAGCCTGCGCTGTATGCAGCTCAATCNCCGATCGCCGCACCACACGAA
L. tropica  358 TGATCCTGGACCTAGCCTGCGCTGTATGCAGCTGAATCACCGATTGCTGCACCACACGAA

L. major    417 ACGGTGCTGGCCAGCCCGGCTCAATGC-----------AGCAACGGTAGGTGAAGTGCTA
L. tropica  418 ACGGTGCTGGTCAGCCCGGCTCGATACAGCCGTCGTGCAGCAGCGGTAGGTGAACTGCTA

L. major    466 TTTTAAGGGTGAGCCTGTCGACTTTTTTGCGATAGACGGACGCGCATTCCCCCCCCCCCA
L. tropica  478 TTTTAAGGGTGAGCTTGTTGACTTTTTCGCGGTAGATGCACACGCGTTGCCAAAACAACA

L. major    526 ACCCAAAAAAAAACGCTCTCTCTCTCTGTTTTGCTTTCNTGCGCTTTGTGTTGCGCTCTT
L. tropica  538 AAGAAAACGCG--CGCTCTCTCTCTGTGTTGTGCTCCCTTTGCGTTTGTCTTGCGTTCTT

L. major    586 TATCATCTG--CTTTTTAGTCCAATGGTGCTCTATTTTTGTGCAGAAGGGTAAACTTGTC
L. tropica  596 ATCCTCTTCTTTTTTCAGTCTGGGTGGTGCTCTCTTTTGGTGCAGATGGGTGAAACTTGT

L. major    644 GC--CGGTAAGG----GAGCAACATGGTGGCCAACGATGACGTTGTCAAACC--TGTGTG
L. tropica  656 TCGCTCGTTAAGGGGGGCGACATGGGTCCGCCAACGATGACGCTCTTCAAAGCTGGTGTG

L. major    696 ATGTTTGCTTCG-CCTCGTACTGCTGCACTATCAAGGA--T-ACGAAG----GCGAATGG
L. tropica  716 ATGTTTGCTCTTGCTTCGTGCTGCTGCCACTTATCAGGAATTACGAAAGGCTACCTCCGG

L. major    748 GTCTATCTACACATTTGCGTCGGCGTCGTGTGCGCAGTGAAGTGAGTGTGTGTGTGTGTG
L. tropica  776 TTCAATCTACACCATTGGCGTCCGGGTCT----------------------------GTG

L. major    808 TGTGTGCTGCGAACGATCGATGCGC-ATGACTTTCAAAAGGTG--TGCAGGTCTCATCCA
L. tropica  808 TGCACCCGTGGAAAAGTTGTGGGTTGTGGACTGCAAAAGAGTGTGTGAGCTTCGGTCCCA

L. major    865 CAGCATCGTTTTCGTCCTAGGTGGCGTCTTCTTCGACAATTCGATGCGTTGGCTATTGCC
L. tropica  868 CAGGCATCGTTTCCTCGTCGATTGCGTCT--TTCCGTGGATCCATTGCGTTGCTATTGGC
```

```
L. major    925 TGTTGCCGTGAAAG-TATATAGTAGCTGTACCGCATGTCTGTGGTTCGGCTACC--ACCG
L. tropica  926 TGTTTTCGTGAAAGTATCTTAGTAGCTGTACTGCGTGCCTTTGGTGCAGCTAACCACCGT

L. major    982 TATACTGCTATGTTTTGACATTTTGAGGAGTTCTCTCTCGCGAAAAC---AAAGCGAAAA
L. tropica  986 AATATGGCTATGTTTTGACATTTGAGGAGCTTCTCTCTCGCGAAAGAAAAAGGCGAAACA

L. major   1039 AGTGCCGTTAAGAACTCTCATGTCCCTTTGAGAAGATTGATCAGTGACAACGACGAGTCT
L. tropica 1046 AGTGCCGTTAAGAACTCTCGTGACCCTTTGAGCAGATTGGGCAGTGAAAACGACAAGTCT

L. major   1099 AGCAAATGGACTTTCCTTT--TTGTTTCAGCCTTGCTCATATGTGTGATGTTCCTACGTG
L. tropica 1106 AGCAAATAGACTTTCCTCTTTTTATTTCAGCCTTGCTCATCTCTATGATGTTCCTACGTG

L. major   1157 CTTATCCTTTGAAGTTTCTTTTCTCAGCG------CTCCTT-TCGCTCTTGCGGCTGATA
L. tropica 1166 CTTATCTGTTGAAGTTTTTCTCTTCTCTTTCAGCGCTCTTTTCTCTCTTGCTGCTGATA

L. major   1210 GCACTGAACAAAACGTTATTGCCGGGAAACCTTT-CCTTTCTGCGTTGTTTTAATATATA
L. tropica 1226 GTACTGAATTAAACGTCATTGCTGCAAAATCTTTTTCTTTCTGTGTTGTTCCAAGATGAT

L. major   1269 TATATATATGTCTGTATCTTTTTTGTGTCTCACCCTCTTCTTTCCACTCTTTCTCCTTG
L. tropica 1286 TATATATGACTCTT----------TTTTGTCTCACCCTTCTTTGCACCCTCTCTCCTTG

L. major   1329 TTCCCCCTTT-TTTC-----GTTACGGAAAGCGAACTGTCTAACGCTTTGCATTTCTTTG
L. tropica 1336 CTACCCCTTTTTTTCTTTACTGAAAGCGAACTTTTGTTCTAACGCTTTGCATTTCTTCG

L. major   1383 GTTGAATGCCAGATTTCACGCGAAGCTGAGAAGGTGAACCTAGTTATCAGCAGCGTACTC
L. tropica 1396 GTTGACTGCCGGATTTCAAGAGAAGCTGAGAAGGTGAACCGTGTTGCCAGCAGTGTACTC

L. major   1443 TTCCTACTACCTCTATCACTTTCTTCTAACACTTCCACACAAACACACACACACACACAC
L. tropica 1456 TTCCCACTACCTCTATCACTTTCTTGTAACACTTCCACACAGACACAGA--CACACACAC

L. major   1503 ACACCATGTTGCTGTGCGAATGCGAACCTGAGTGGGTGCAAAGTGTGATTCCCATAACGA
L. tropica 1514 ACACCATGTCGCTGTGCGAATGCGAACCTGAGTGGCTGCAAAGCGTGATTCCCATAACGA

L. major   1563 TGAGCGTCACTTCACACTTACATTTTCTTGTTTTCTTTTCCTTTTGTTTTGTCACTTTCC
L. tropica 1574 TGAGCGTCACGGCACGTTTACATTTTCTTGATTGCTTTCCTTTCCTTTT---CACTCTCC

L. major   1623 GGCATACCATGGGAAAAAGGTGAGTGCACACGGGGCGCTTCTTGCGTGAGGCTTGGCTTG
L. tropica 1631 GGCA-TATCGTGCGAAAAGATGAGTGCACACGGCGTACTTCTTGCGTGAGGCTTGGCTTG

L. major   1683 CCTTGCACGCACTAGGTGACCCAGCGTGACGGCAGGCAATGGATGCTGATGCTGTTTGAA
L. tropica 1690 CCTTGCACGCGCTAGGTGACCCAGCGTGGCGGTAGGACATAGACGCCGATGCTGTCTGAA

L. major   1743 AAAGAGGCAGGTGAAGTGCTATAAGTTTTCGTCGCAAGGAACTTTCTCGTGGCAAACGAA
L. tropica 1750 AAAGAAGCAGGTGAAGTTCTATAGGGTTTCGTCGCTAAGGGCTTTCCAGTGGC-------

L. major   1803 CGGCAGCAAAAAAAGGGAG
L. tropica      -------------------
```
