# Supplementary material for: Species-Specific Antimonial Sensitivity in Leishmania Is Driven by Post-Transcriptional Regulation of AQP1
Source: PLoS Negl Trop Dis. 2015 Feb 25;9(2):e0003500. doi: 10.1371/journal.pntd.0003500 (PMC4340957; doi:10.1371/journal.pntd.0003500)
Supplement: S6 Fig — The 3’-UTRs from each species were cloned and sequenced as described in the materials and methods. Sequences were aligned using Clustal omega and Boxshade server. The dashes indicate the gaps introduced to maximize sequence alignment. (PDF) [file pntd.0003500.s006.pdf]

Figure S6

```
L. braziliensis    1 AGCTGCACTGCTGCTCTAACTTGCTCTGCCATGTATACAACGTTTAGAGCTCGTACGTAA
L. panamensis      1 AGATGCACTGCTGTGCTAACTTGCTCTGCCATGCCTACAGCGTTTAGAGCTCGTACGTAA

L. braziliensis   61 TGACACGTTATCCTTTGCTTTATTACTACTACCCATACAATATACTGTTTTACACTTGAC
L. panamensis     61 TGACACGTTATCCTTTGCTTTATTACTACTACCCATACAATATACTGTTTTACACTTGAC

L. braziliensis  121 CTGATGACCCGAGGCACCTCACTGCTGTATTAACATTGAGAACCAACTCTGTGGAGAAGC
L. panamensis    121 CTGATGACCCGAGGCACCTCACTGCTGTATCAACATTGAGAACCCACTCTGTGGAGAAGC

L. braziliensis  181 CAAGAGCCTGCAGTATTCCCTCGCATACGACTGTGCAAACTCTTGTAACTGGCAAACATA
L. panamensis    181 CAAGAGCCTGCAGTATTCCCTCACATACGACTGTGCAAACTCTTGTAGCTGGCAAACGTA

L. braziliensis  241 TACAGACAGGTGCTGTACTAGGGAGGCTTACAAGCATCATAAAGCTTGGAGCAGCTAACC
L. panamensis    241 TACAGACAGGTGCTGTGCTAGGGAGGCTTACAAGCATCATAAAGCTTGGAGCAGCTAACC

L. braziliensis  301 TCACATCCTGTTCGCAATCTAGCGAATAAACAAACCCTCCTGCTCTTTATTTTCCGCTGT
L. panamensis    301 TCACATCCTGTTCGCAATCTAGCGAATAAACAAACCCTCCTGCTCTTTATTTTCTGCTGT

L. braziliensis  361 TGCTGCGGCGTGTGAGTGTTGGGCTTAGTCCAGGAGTGATCCTGGGCTATGTGCAGTAGA
L. panamensis    361 TGCTGCAGCGTGTTAGTGTTGGGCTTAGTCCTGGAGTGATCCTGGGCTATGTGCAGTAGA

L. braziliensis  421 ACTGCAGCTGTTTCGACGATCTCCGACAGTATTGACAGCCCCTATTGCTTGAAGTAGCAA
L. panamensis    421 ACTGCAGCTATCTCAACGATCTCCAACAGTATTGACAGCCCCTATTGCTTGAAGTAGCAA

L. braziliensis  481 CAAGGTAGCCGTTGTGGGGAGAACTGTGGTTGTACGTGTCTATGGGTGCCCACGTGCTCG
L. panamensis    481 CAAGGTAGCCGATGTGGGGAGAACTGTGATTGTGCGCGTCTATGGGTGCCCACGTGCTCG

L. braziliensis  541 CCATGGAATAGACACGTTCC------------CCCCCAAAATGCTTTCTCGTTGTCCAGC
L. panamensis    541 CCATGGAGTAGACACGTTCCTTCCCCCCCCCCCCCCCCAAATGCTTTCTCTTTGTCCAGC

L. braziliensis  589 TCCCTTGTTCGTCTTGTTTTATTTTTTATTTTATTGTGCTACCCCTTAGCACTGCTTCTG
L. panamensis    601 TCCCTTGTTCGTCTTGTTTTATTTTTTATTTTATTTTGCTACCCCTTAGCACTGCTTCTG

L. braziliensis  649 CCAGTTATTTGGTTGCTGTACGGGATTTTGCGCAG-GGGCTGCTATTGTCCTCGGCAGGG
L. panamensis    661 CCAGTTATTTGGTTGCTGTACGGGATTTTGCGCAGGGGGCTGCTATTGTCCTCGGCAGGG

L. braziliensis  708 GGGAGCATCATCGCAGTCAAAGACAGACTTGTTAACCAGATGTTGTCTCTTCTGCCCCGC
L. panamensis    721 GGGAGCATCATCACAGTCAAAGACAAACTTGTTAACCAGATGTTGTCTCTTCTGCTCCGC

L. braziliensis  768 GCTGCTGCAAAGGTGTGGAGAAGCAGGCCACTCTGGATATCTCTGTATTTGTTCCCTACG
L. panamensis    781 GCTGCTGCAAAGGTGTGGAGAAGCAGGCCACTCTGGATATCTCTGTATTTGTTCCCTACG

L. braziliensis  828 TGATGCGCGTGATAAGATATATGTTTGCTTTCAAGGATCACCGCGCATAACTGTCCACAG
L. panamensis    841 TGATGTGCGTGATAAGATATATGTTTGCTTTCAAGGATCACCGCGCATAACTGTCCATAG

L. braziliensis  888 AGTGTGCGGGCCCTGTGCCGAGCATCTTCATTGGCGTTCTCTGTTTTGTCGTGACGTTCT
L. panamensis    901 AGTGTGCGGGCCCTGTGCCGAGCATCTTCATTGACGTTCTCTGT-----CGTGACATCCT
```

```
L. braziliensis  948 TCTCCCACATTCCACGTGGCGGTTACAACAACGATCTTACACTGTTGCACGTTGGCATTT
L. panamensis    956 TCTCCCACATTCCACGTGGCGGGTACAACAACGATCTTACACTGTTGCACGTTGGCATTT

L. braziliensis 1008 TGAGGAGGTTTCGCGTAAAACCATACGAGTGTACCCGCAGCTCTGTGGACGCTTGCCCAG
L. panamensis   1016 TGAGGAGGTTTCGCGTAAAACCATACGAGTGTACCCGCAGCTCTGTGGACGCTTGCCCAG

L. braziliensis 1068 AACAGCCAACAAGAAATGAGGAATCCAAGACATTGTCTTTTCTTGCCTGATTGTTTTGAT
L. panamensis   1076 AACAGCCAACAAAAAATGAGGAATCCAAGACATGGTCTTTTCTTGCCTGAATGTTTCGAT

L. braziliensis 1128 GGCTTTTTTGTCGAGGTGTGTGTGTCTTTCTGCAGATTCCCGCCTTTTTTTTTTCATAAC
L. panamensis   1136 GGCTTTTTTGTCGAGGTGTGTGTGTCTTTCTGCAGTTTCCCGCCTTT-TTTTTTCATAAC

L. braziliensis 1188 ACTGCCGCGTGTTTGTGAGCGACCGCAGTGTAGAACTGTCTTAAAAAGAAAATGCAAAAC
L. panamensis   1195 ACTGCCGCGTGTTTGTGAGCGACCGCAGTGTAGAACCGTCTTAAAAAGAAAATGCAAAAC

L. braziliensis 1248 GTTTTTTCTCCTATTTTTTTGTTTTTTTTTTGCTGAAACCAAAATGAAGTTTGTCTCTGC
L. panamensis   1255 GTTTTTTCTCCTATTTTTTTGCTTTT-----GCTCAAACCAAAATGAAGTTTGTCTCTGC

L. braziliensis 1308 GGCTT--TTTTTCCTCCACTCTTTTCGTGCCATGTTTGCTTGAAAATGTGCTGTTTC--C
L. panamensis   1310 GTCTTTTTTTTTTCTCCACTCTGTTTGTGCCATGTTTGCTTGAAAATGTGCTGTTTCCCT

L. braziliensis 1364 CCCTTTTCGGTGCTTCTATTCAATGCTTAAGATGAAAGGAAGTCAAGGCGACGAATTGTA
L. panamensis   1370 CCCTTTTCGGTGCTTCTATTCAATGCTTAAGATAAAAGGAAGTCAAGGCGACGAATTCTA

L. braziliensis 1424 TTATCAACCGTCTACTCTTCCCACTATCTCTGTCCTATTCTACGGCGCACTTCGTACGCA
L. panamensis   1430 TTATCAACCGTCTACTCTTCCCACTATCTCTCTCCTGTTCTACGGCGCACTTCGTACGCA

L. braziliensis 1484 ACCCACCCACCCACCCACCCACAATGCCACTGCAAAATGGGAACTCAGGAGTTGTGAAGA
L. panamensis   1490 ACCCACCC--------ACCCACAATGCCACTGCAAACTGGGAACTCAGGGGTTGTGAAGA

L. braziliensis 1544 GCGGATCCTACAGCTCAGAGTACCCTGCGCCGAAACAGCGAGTTTTTTTGTTGTTGTTTT
L. panamensis   1542 GCGGATCCTACAGCTCGGAGTACACTGCGCCGAAACAGTGAGTTT-TTTGTTGTTGTTTT

L. braziliensis 1604 CTCAGCGGCTAGGATGCGAAAGACTCAGTGCCCACGAGATTCTTCTTGCAGGAGGCCTGG
L. panamensis   1601 CTCAGCGGCTAGGATGCGAAAGACTCAGTGCCCACGAGATTCTTCTTGCAGGGGGCCTGG

L. braziliensis 1664 TTTGCCTCTCAGGCGCCAGGCAAGTGAGTATCGTGGTATCAACTGGATGCTGATGCGGAC
L. panamensis   1661 TTTGCCTCTCAGGCGCCAGGTAAGTGAGTATCGTGGTATTAACGGGAGGCTGATGCGGAC

L. braziliensis 1724 TCAAAGAGAAACGATATACTTTCCATAGGTTTCCGTCGCTACTGACGTCCCAGCGGCGAA
L. panamensis   1721 TCAAAGAGAAGCGATTTACTTTCCATAGGTTTCCGTCGCTACTGACGTCCCAGCGGCGAA

L. braziliensis 1784 AAAAGAGCACGCTGTCAGCATAAG-----
L. panamensis   1781 AAAAAAGCACGCTGTCAGCGTAAGGTAAG
```
