# Supplementary figures and images for: Species-Specific Antimonial Sensitivity in Leishmania Is Driven by Post-Transcriptional Regulation of AQP1
Source: PLoS Negl Trop Dis. 2015 Feb 25;9(2):e0003500. doi: 10.1371/journal.pntd.0003500 (PMC4340957; doi:10.1371/journal.pntd.0003500)

Figure S7

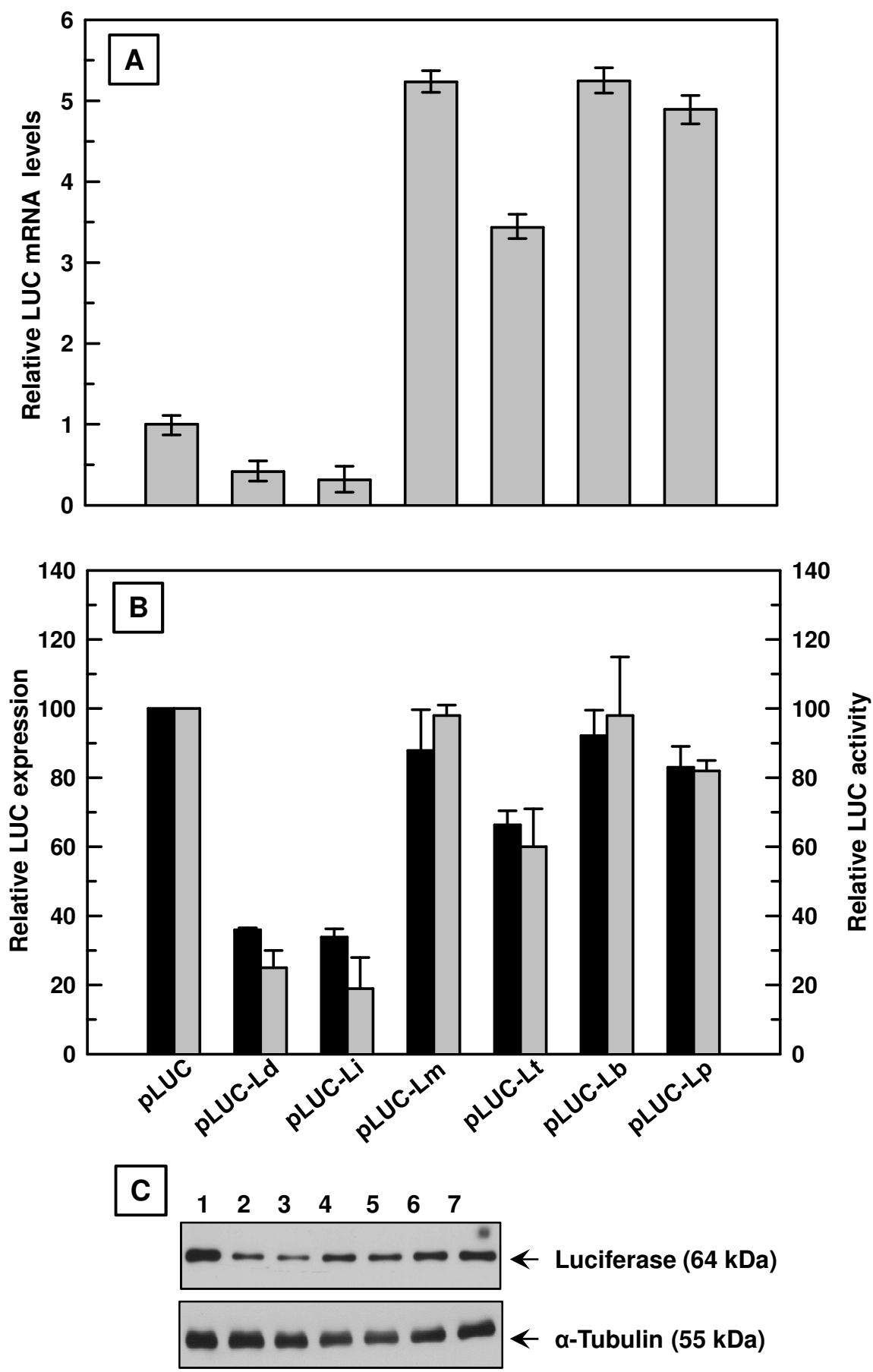

Supplement: S7 Fig — A. LUC mRNA levels: Total RNA was isolated from promastigotes of L. infantum expressing different chimeric constructs of LUC and LUC mRNA expression levels were estimated using qPCR. Relative (with respect to LUC) LUC mRNA expression levels were calculated using 2-ΔΔCt method. Data were expressed as mean ± SD of three independent experiments in triplicate. B. LUC activity and expression: Estimation of LUC activity (□) was carried out using whole cell lysates. Percent LUC activity was calculated keeping vector control at 100%. Data were expressed as mean ± SE of three independent experiments in triplicate. C. Representative western blot analysis of transfected promastigotes: Whole cell (1 x 10^6^/lane) lysates of different transfectants were fractionated on SDS-PAGE and blotted onto nitrocellulose membrane. Levels of LUC expressions were detected using an anti-luciferase antibody. α-tubulin was used as loading control. Lanes: 1. pLUC, 2. pLUC-Ld, 3. pLUC-Li, 4. pLUC-Lm, 5. pLUC-Lt, 6. pLUC-Lb, and 7. pLUC-Lp. Amount of luciferase expression (■) relative to cells transfected with pSPYNEOαLUC was estimated by densitometric analysis using ImageJ software followed by normalization against the amount of α-tubulin of the respective cells. Error bars were calculated from the mean ± SE of two independent experiments. Ld- L. donovani, Li- L. infantum, Lm- L. major, Lt- L. tropica, Lb- L. braziliensis, Lp- L. panamensis. (PDF) [file pntd.0003500.s007.pdf]

Figure S8

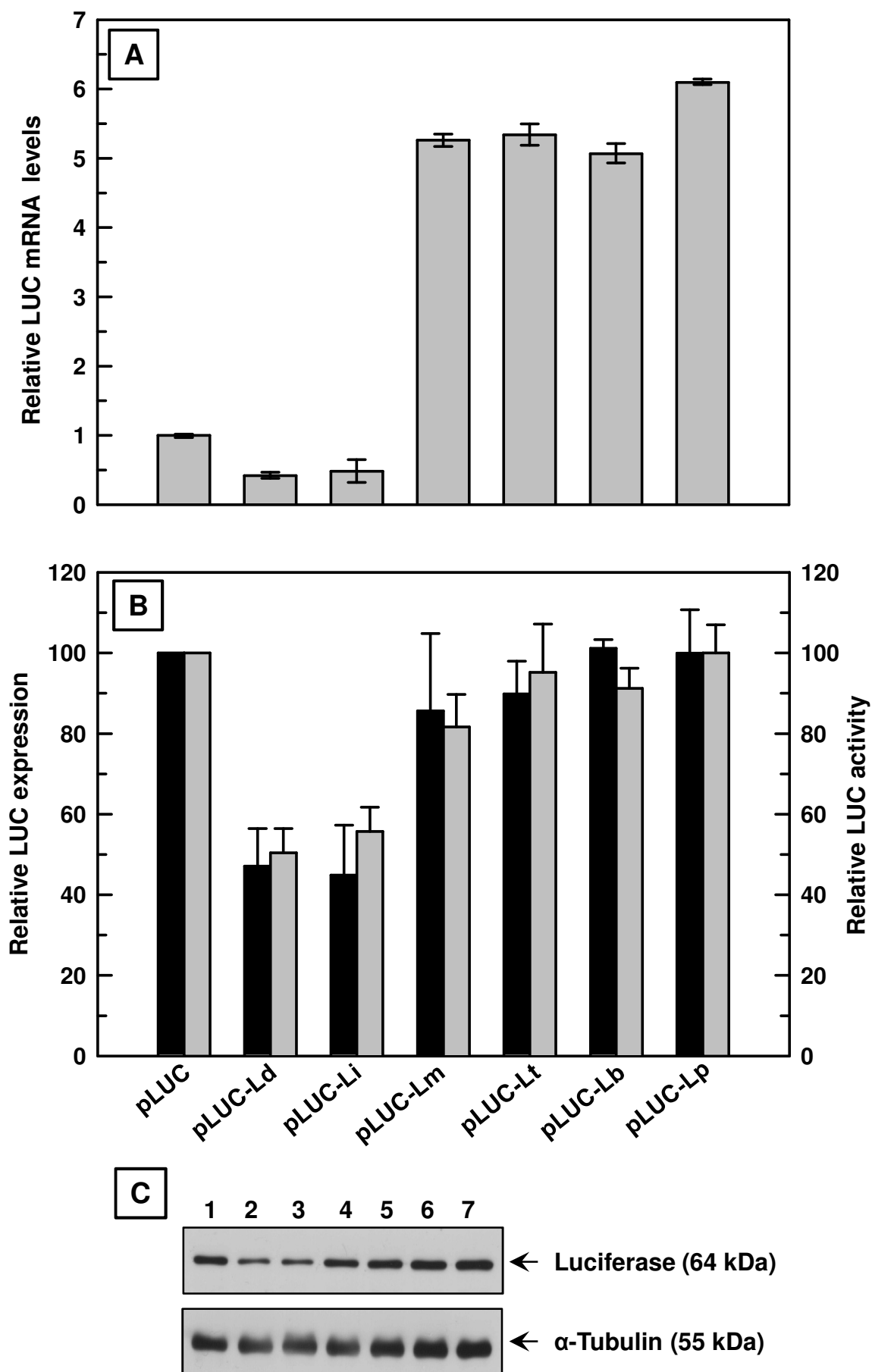

Supplement: S8 Fig — A. LUC mRNA levels: Total RNA was isolated from promastigotes of L. tropica expressing different chimeric constructs of LUC, and LUC mRNA expression levels were estimated using qPCR. Relative (with respect to LUC) LUC mRNA expression levels were calculated using 2-ΔΔCt method. Data were expressed as mean ± SD of three independent experiments in triplicate. B. LUC activity and expression: Estimation of LUC activity (□) was carried out using whole cell lysates. Percent LUC activity was calculated keeping vector control at 100%. Data were expressed as mean ± SE of three independent experiments in triplicate. C. Representative Western blot analysis of transfected promastigotes: Whole cell (1 x 10^6^/lane) lysates of different transfectants were fractionated on SDS-PAGE and blotted onto nitrocellulose membrane. Levels of LUC expressions were detected using an anti-luciferase antibody. α-tubulin was used as loading control. Lanes: 1. pLUC, 2. pLUC-Ld, 3. pLUC-Li, 4. pLUC-Lm, 5. pLUC-Lt, 6. pLUC-Lb, and 7. pLUC-Lp. Amount of luciferase expression (■) relative to cells transfected with pSPYNEOαLUC was estimated by densitometric analysis using ImageJ software followed by normalization against the amount of α-tubulin of the respective cells. Error bars were calculated from the mean ± SE of two independent experiments. Ld- L. donovani, Li- L. infantum, Lm- L. major, Lt- L. tropica, Lb- L. braziliensis, Lp- L. panamensis. (PDF) [file pntd.0003500.s008.pdf]

Figure S9

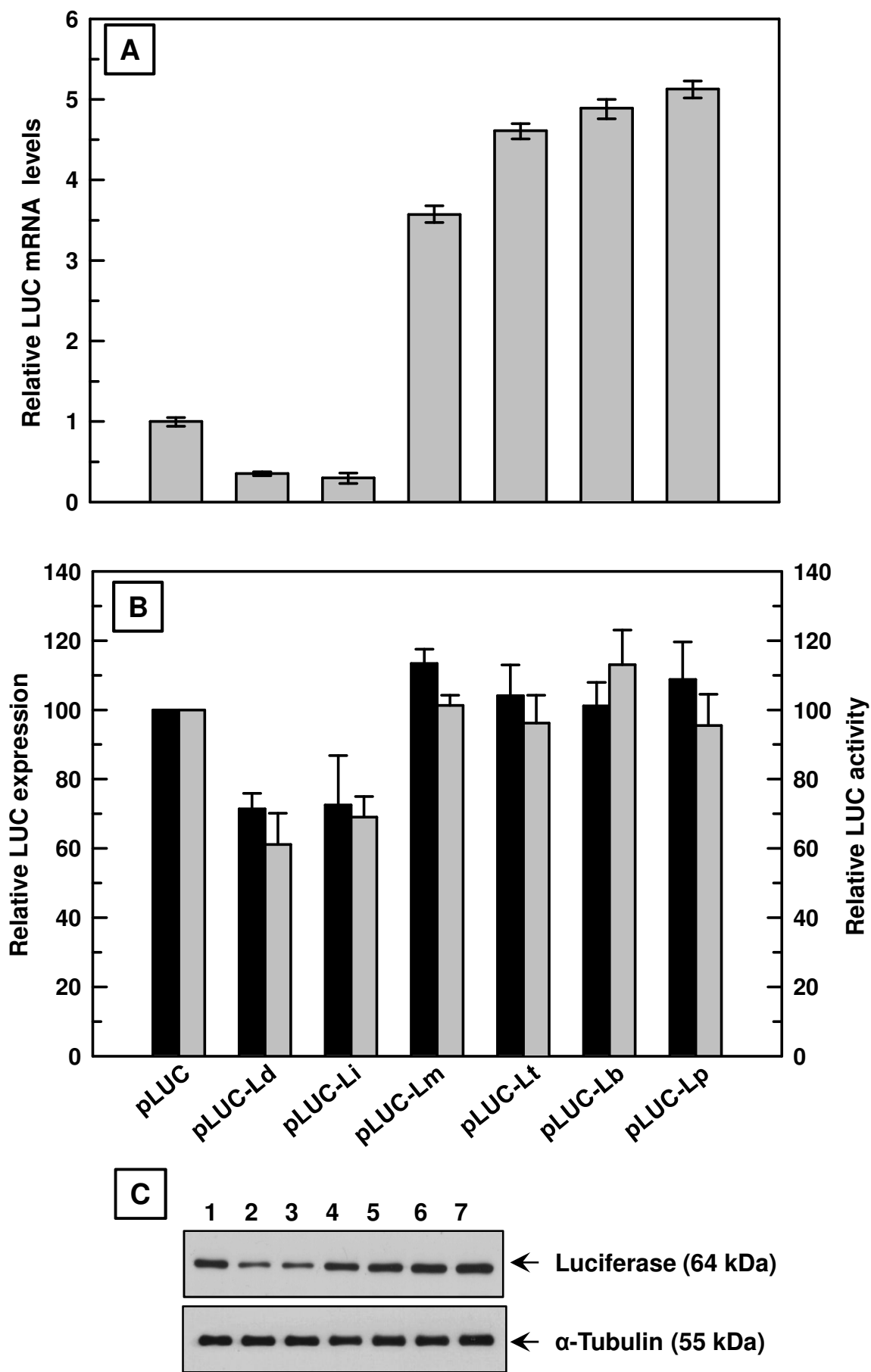

Supplement: S9 Fig — A. LUC mRNA levels: Total RNA was isolated from promastigotes of L. panamensis expressing different chimeric constructs of LUC and LUC mRNA expression levels were estimated using qPCR. Relative (with respect to LUC) LUC mRNA expression levels were calculated using 2-ΔΔCt method. Data were expressed as mean ± SD of three independent experiments in triplicate. B. LUC activity and expression: Estimation of LUC activity (□) was carried out using whole cell lysates. Percent LUC activity was calculated keeping vector control at 100%. Data were expressed as mean ± SE of three independent experiments in triplicate. C. Representative western blot analysis of transfected promastigotes: Whole cell (1 x 10^6^/lane) lysates of different transfectants were fractionated on SDS-PAGE and blotted onto nitrocellulose membrane. Levels of LUC expressions were detected using an anti-luciferase antibody. α-tubulin was used as loading control. Lanes: 1. pLUC, 2. pLUC-Ld, 3. pLUC-Li, 4. pLUC-Lm, 5. pLUC-Lt, 6. pLUC-Lb, and 7. pLUC-Lp. Amount of luciferase expression (■) relative to cells transfected with pSPYNEOαLUC was estimated by densitometric analysis using ImageJ software followed by normalization against the amount of α-tubulin of the respective cells. Error bars were calculated from the mean ± SE of two independent experiments. Ld- L. donovani, Li- L. infantum, Lm- L. major, Lt- L. tropica, Lb- L. braziliensis, Lp- L. panamensis. (PDF) [file pntd.0003500.s009.pdf]

Figure S10

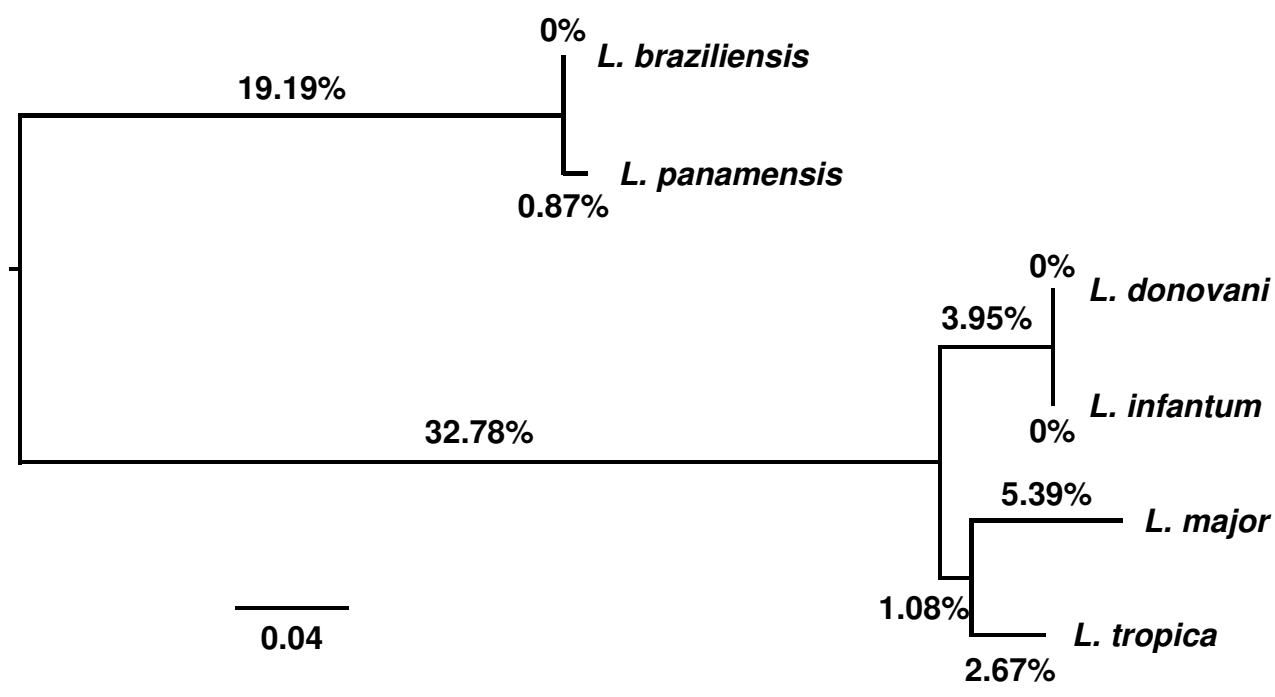

Supplement: S10 Fig — Multiple sequence alignment for the ORF (open reading frame) was done using MUSCLE. This helps to eliminate poorly aligned positions and divergent regions. Phylogeny was built using PhyML program and tree was made using Figtree. Midpoint rooted tree depicts two different clades for mucocutaneous species (L.braziliensis and L.panamensis), cutaneous (L.major and L.tropica) and visceral species (L.donovani and L.infantum) based on their ORF sequences. According to the tree, there is 0.8% difference between the ORF sequences of L.braziliensis and L.panamensis. Sequences are similar for both the visceral species, whereas there is 2.8% difference between L.major and L.tropica sequences from the branch node. (PDF) [file pntd.0003500.s010.pdf]

Figure S12

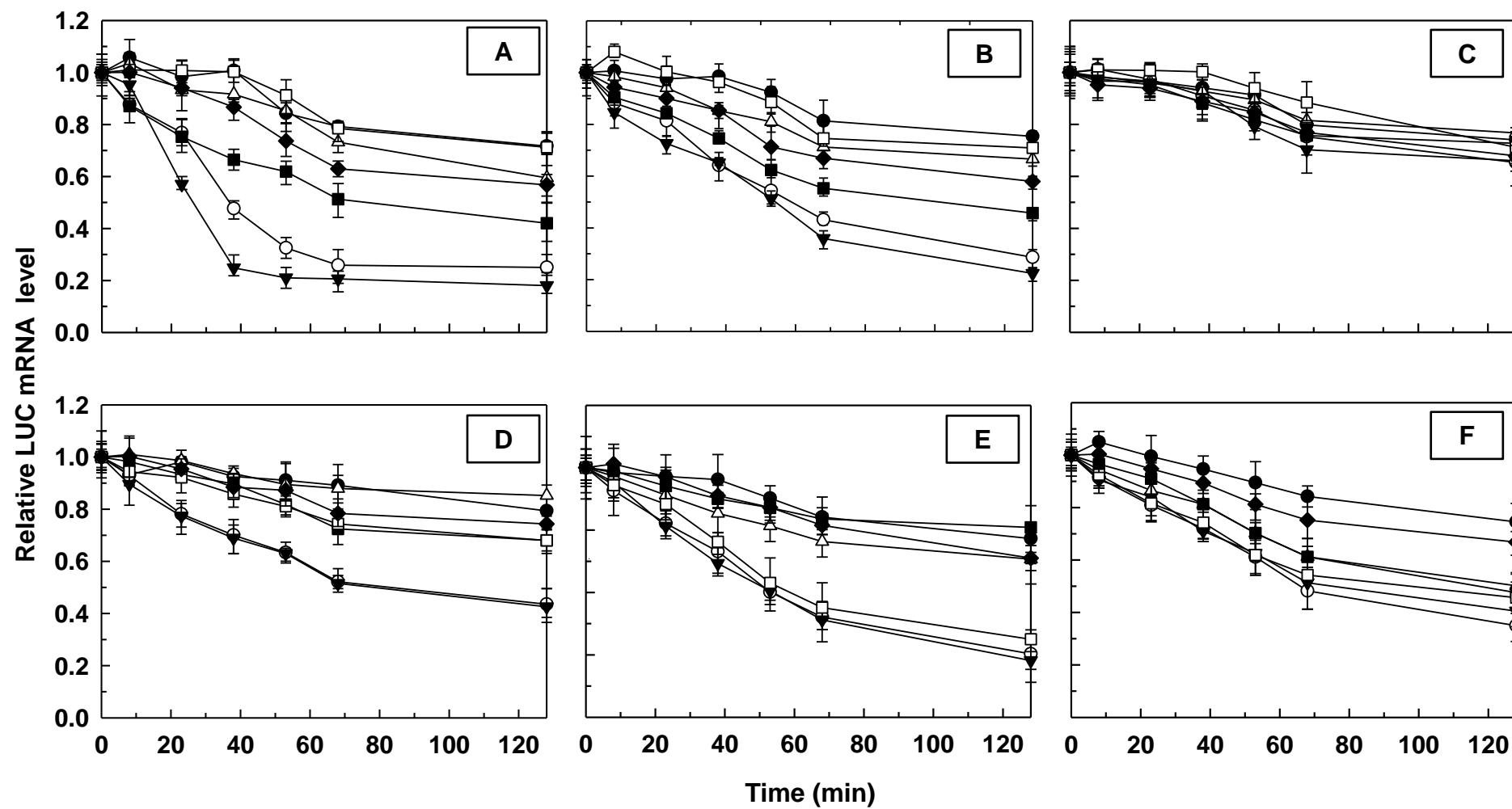

Supplement: S12 Fig — Promastigotes of different species of Leishmania (each transfected with six different AQP1 3’-UTR chimeric constructs as described in Fig. 5A) were exposed to sinefungin followed by actinomycin D. Cells were harvested just before the exposure of actinomycin D (0 minute) and at different time points after the exposure with actinomycin D. Total RNA was isolated, and LUC mRNA levels were estimated using qPCR. Relative (with respect to 0 minute) LUC mRNA levels were calculated using 2-ΔΔCt method. Data were expressed as mean ± SD of three independent experiments in triplicate.-●- pLUC,-○- pLUC-Ld,-▼- pLUC-Li,-△-pLUC-Lm,-■- pLUC-Lt,-□- pLUC-Lb,-◆- pLUC-Lp. A: L. donovani, B: L. infantum, C. L. major, D. L. tropica, E. L. braziliensis, and F. L. panamensis. (PDF) [file pntd.0003500.s012.pdf]
