# Supplementary material for: Species-Specific Antimonial Sensitivity in Leishmania Is Driven by Post-Transcriptional Regulation of AQP1
Source: PLoS Negl Trop Dis. 2015 Feb 25;9(2):e0003500. doi: 10.1371/journal.pntd.0003500 (PMC4340957; doi:10.1371/journal.pntd.0003500)
Supplement: S11 Fig — The protein source and GenBank accession numbers of the aligned sequences are L. donovani (ABQ84980); L. infantum (CAM70318); L. major (XP_001684986); L. tropica (not annotated). Sequences were aligned using ClustalW2 and Boxshade server. The dashes indicate the gaps introduced to maximize sequence alignment. (PDF) [file pntd.0003500.s011.pdf]

Figure S11

```
L. major       1 GCGTGCTTCGGTACCCCTGCCCTCTCTTTCCTGGGCGTACAAACACCACAGCTCGTGCTT
L. tropica     1 GCGTGCTTCGCTA--CCCCTGCTCTCCTTGCTGGGCGTACAAACTCCACAGCTCACGCTG
L. donovani    1 ACGTGCTTCGCTA--CCCCTGCTCTCTATGCTGGGTGTACAAACACCACAGCTCGTGCTG
L. infantum    1 ACGTGCTTCGCTA--CCCCTGCTCTCTATGCTGGGTGTACAAACACCACAGCTCGTGCTG

L. major      61 GCTAACCATTTAATATTCTAAATTCATTATTGTTCTTATTTTACTGCTTTTCCCTTAAGC
L. tropica    59 GCTGACCATTTACTTTTTCCAATGCATTGTTT-TGGGGTATTTTTCTGCTTTCCTTGTGC
L. donovani   59 ACTGACCATTTACTCTTTCCGATTTATTATTATTATTATTTTTCTCTGCTTTCCCTCTGC
L. infantum   59 ACTGACCATTTACTCTTTCCGATTTATTATTATTATTATTTTTCTCTGCTTTCCCTCTGC

L. major     121 CTTGATGACGCAAGCCACCTCACCGTTCTATCAGGGTCCAATGGCCACTCTGCGGGAACG
L. tropica   118 CTTGATGGCGCGAGCCACCTCACCGCTCTATCAGGGTCCAGTGCCCACTCTGCGGGAACG
L. donovani  119 CTTTATGACGCGAGCCACCTCACCGTTGTATCAGGGTCCCGTGCCCACTCTGCGGGAACG
L. infantum  119 CTTTATGACGCGAGCCACCTCACCGTTGTATCAGGGTCCCGTGCCCACTCTGCGGGAACG

L. major     181 TCAAAAGCCTGCAGCCTGCCCTCGTGTCGGCGGAGAGGCTGGAGCTGGCACTGTGCCGGA
L. tropica   178 ACAAAAGCCTGCAGCCTGCCCTCGTGTCGGCAGACAGGCTTGAGCTGACACTGTACCGGA
L. donovani  179 TCGAAAGCCTGCACCCTGCCATCGCCTCGGCGGACAGGCTTGGGCTGACACTGTACCGGA
L. infantum  179 TCGAAAGCCTGCACCCTGCCATCGCCTCGGCGGACAGGCTTGTGCTGACACTGTACCGGA

L. major     241 GAGACTCTCGGCTACGATGCTGGTTCTATCATCTCACTGCCAGTCGTTTCAGCGACTCGG
L. tropica   238 GAGACTCGCCGCTACAATGCTGTTTCTATCAACCCGCTGCCAGTCGCTTCAGCGACTCAG
L. donovani  239 GACACTCGCGGCTACGATGCTGTTTCTATCATCTCGCTGCCAGTCGTTTCGACGACTCAG
L. infantum  239 GACACTCGCGGCTACGATGCTGTTTCTATCATCTCGCTGCCAGTCGTTTCGACGACTCAG

L. major     301 CAAGCATGCGCTTCCACTCAC----TGTCTGCTTTTGCAGCGCTTCGGTAATACCGAGCG
L. tropica   298 CAAGCATGCGCTTCCACTCACTGCTTTTCTGCTTTTGCGGCGCTTCGGTAATACCGAGCG
L. donovani  299 CAAGCATGCGCTTCCAATCATTGCCCTTCTGCTTTTGCGGCGCTTCGGTAATACCGAGCG
L. infantum  299 CAAGCATGCGCTTCCAATCATTGCCCTTCTGCTTTTGCGGCGCTTCGGTAATACCGAGTG

L. major     357 TGATCCCGCGCCTAGCCTGCGCTGTATGCAGCTCAATCNCCGATCGCCGCACCACACGAA
L. tropica   358 TGATCCTGGACCTAGCCTGCGCTGTATGCAGCTGAATCACCGATTGCTGCACCACACGAA
L. donovani  359 TGATTCTGGACCTGGCCTGCGCTGTATGCAGCGGAATCACGGTTTGCTGCACCACACGAA
L. infantum  359 TGATTCTGGACCTGGCCTGCGCTGTATGCAGCGGAATCACGGTTTTCTGCACCACACGAA

L. major     417 ACGGTGCTGGCCAGCCCGGCTCAATG-----------CAGCAACGGTAGGTGAAGTGCTA
L. tropica   418 ACGGTGCTGGTCAGCCCGGCTCGATACAGCCGTCGTGCAGCAGCGGTAGGTGAACTGCTA
L. donovani  419 AGGGTGCTAGTCATCCCGTCTCGATACTGCCGTCGTGCAGCAGCCGTAGGTGACCTGCTA
L. infantum  419 AGGGTGCTAGTCATCCCGTCTCGATACTGCCGTCGTGCAGCAGCCGTAG-TGACCTGCTA

L. major     466 TTTTAAGGGTGAGCCTGTCGACTTTTTTGCGATAGACGGACGCGCATTCCCCCCCCCCCA
L. tropica   478 TTTTAAGGGTGAGCTTGTTGACTTTTTCGCGGTAGATGCACACGCGTTGCCAAAACAACA
L. donovani  479 TTTTAAGGGTCAGTTTGTTGACTTTCTTGCGAGAGATGAACACGCGTTGCCAAATAAAAA
L. infantum  478 TTTTAAGGGTCAGTTTGTTGACTTTCTTGCGAGAGATGAACACGCGTTGCCAAATAAAAA

L. major     526 ACCCAAAAAAAAACGCTCTCTCTCTCTGTTTTGCTTTCNTGCGCTTTGTGTTGCGCTCTT
L. tropica   538 AAGAAAACGCGCGCTCTCTCTCTG--TGTTGTGCTCCCTTTGCGTTTGTCTTGCGTTCTT
L. donovani  539 AAGAGCATGCTCTCTCTCTCTCTCTCTGCTGTGGTCCATTTGCGTTTATGCTGCTTTCTT
L. infantum  538 AAGAGCATGCTCTCTCTCTCTCTCTCTGCTGTGGTCCATTTGCGTTTATGCTGCTTTCTT

L. major     586 TATCATCTGC--TTTTTAGTCCAATGGTGCTCTATTTTTGTGCAGAAGGGTAAACTTGTC
L. tropica   596 ATCCTCTTCTTTTTTCAGTCTGGGTGGTGCTCTCTTTTGGTGCAGATGGGTGAAACTTGT
```

```
L. donovani  599 TACCATTTTC--TTCTCAGCCTGCTGGTGCTCTATTTTTGTGCAGAAGGGTGAACTTTTC
L. infantum  598 TACCATTTCT--TCTCAGCCTGCTGGTGCTTCTATTTTTGTGCAGAAGGGTGAACTTTTC

L. major     644 GCCGG--TAAGGGAG----CAACATGGTGGCCAACGATGACGTTGTC--AAACCTGTGTG
L. tropica   656 TCGCTCGTTAAGGGGGGCGACATGGGTCCGCCAACGATGACGCTCTTCAAAGCTGGTGTG
L. donovani  657 GCCCG--TAAGGGAG--CGACAT--GGTAGCCAACGACAACTTTCTA--CAGCCTGTGTG
L. infantum  656 GCCCG--TAAGGGAG--CGACAT--GGTAGCCAACGACAACTTTCTA--CAGCCTGTGTG

L. major     696 ATGTTTGCTTCG-CCTCGTACTGCTGCACTATCAA--GGATACGAAGGCGAATGGGTCTA
L. tropica   716 ATGTTTGCTCTTGCTTCGTGCTGCTGCCACTTATCAGGAATTACGAAAGGCTACCTCCGG
L. donovani  709 ATGTCTGCTCTG-CTTCGTGCTGCTGCACTCTCAA--GGATACGGAGGCTATTCGGCCTA
L. infantum  708 ATGTCTGCTCTG-CTTCGTGCTGCTGCACTCTCAA--GGATACGGAGGCTATTCGGCCTA

L. major     753 TCTACACATTTGCGTCGGCGTCGTGTGCGCAGTGAAGTGAGTGTGTGTGTGTGTGTGTGT
L. tropica   776 TTCAATCTACACCATTGGCGTCCGGTCTGTGTGCACCCGTGGAAAAGTTGTGGG----T
L. donovani  766 TCTACACATTTGCTTTGGCGTCGGGTGCACAGTGAAGTTTGTGTGCGTGTGTGTG----T
L. infantum  765 TCTACACATTTGCTTTGGCGTCGGGTGCACAGTGAAGTTTGTGTGCGTGTGTGTG----T

L. major     813 GCTGCGAACGATCGATGCGCAT--GACTTTCAAAAGGTGTGCAGGTCTCATCCACAGCAT
L. tropica   832 T------------------GTGGACTGCAAAAGAGTGTGTGAGCTTCGGTCCCACAGGCA
L. donovani  822 GCTGTCAGCAATCAATACGCATACCTTTCAAAAAAGGTGTGCTGGCCTCGTCCACAGCAC
L. infantum  821 GCTGTCAGCAATCAATACGCATACCTTTCAAAAAAGGTGTGCTGGCCTCGTCCACAGCAC

L. major     871 CGTTTTCGTCCTAGGTGGCGTCTTCTTCGACAATTCGATGCGTTGGCTATTGCCTGTTGC
L. tropica   874 TCGTTTCCTCGTCGATTG-CGTCTTTCCGTGGATCCATTGCGTTGCTATTGGCTGTTTTC
L. donovani  882 CGTTTTCCTCCTACGTGGCGTATTCTCCGAGGATTCAATGCGTTGGCTTTTGCCTGTGTC
L. infantum  881 CGTTTTCCTCCTACGTGGCGTATTCTCCGAGGATTCAATGCGTTGGCTTTTGCCTGTGTC

L. major     931 CGTGAAAGTATATAGTAGCTGTACCGCATGTCTGTGGTTCGGCTACCA--CCGTATACTG
L. tropica   933 GTGAAAGTATCTTAGTAGCTGTACTGCGTGCCTTTGGTGCAGCTAACCACCGTAATATGG
L. donovani  942 CGCGAAAGTATATATTGGCTGTAGTGCATGTCTTTGGTGCGACTACCG--CCGTATACTG
L. infantum  941 CGCGAAAGTATATATTGGCTGTAGTGCATGTCTTTGGTGCGACTACCG--CCGTATACTG

L. major     989 CTATGTTTTGACATTTTGAGGAGTTCTCTCTCGCGAAAACAAA---GCGAAAAAGTGCCG
L. tropica   993 CTATGTTTTGACATTTGAGGAGCTTCTCTCTCGCGAAAGAAAAAGGCGAAACAAGTGCCG
L. donovani 1000 CTATGCTTTGACATTTTGAGGAGCTCCCTCGAGGAAAAAGAAGCGAAAAACAAAACGCCG
L. infantum  999 CTATGCTTTGACATTTTGAGGAGCTCCCTCGAGGAAAAAGAAGCGAAAAACAAAACGCCG

L. major    1046 TTAAGAACTCTCATGTCCCTTTGAGAAGATTGATCAGTGACAACGACGAGTCTAGCAAAT
L. tropica  1053 TTAAGAACTCTCGTGACCCTTTGAGCAGATTGGGCAGTGAAAACGACAAGTCTAGCAAAT
L. donovani 1060 TTAAGAACTCCCGTGACCGTTTGAGCAGACTGGGCAGTGAAAACGACGAGTCTGGCAAAT
L. infantum 1059 TTAAGAACTCCCGTGACCGTTTGAGCAGACTGGGCAGTGAAAACGACGAGTCTGGCAAAT

L. major    1106 GGACTTTCCTTTTTGTT--TCAGCCTTGCTCATATGTGTGATGTTCCTACGTGCTTATCC
L. tropica  1113 AGACTTTCCTCTTTTTATTTCAGCCTTGCTCATCTCTATGATGTTCCTACGTGCTTATCT
L. donovani 1120 GGGCTTTCCTTTTTTTTTC------ATGCTCACCTGTATGATTTTCCTCCGTGCTTATCT
L. infantum 1119 GGGCTTTCCTTTTTTTTTC------ATGCTCACCTGTATGATTTTCCTCCGTGCTTATCT

L. major    1164 TTTGAAGTTTCTTTTCTCAGCGCT--------CCTTTCGCTCTTGCGGCTGATAGCACTGA
L. tropica  1173 GTTGAAGTTTTTCTCTTCTCTTTCAGCGCTCTTTTCTCTCTTTGCTGCTGATAGTACTGA
L. donovani 1174 TTTGAATTTTCTCTCTTCAGCTCT--------CTTTTGCTCTTGCTGCTGATAGCGATGG
L. infantum 1173 TTTGAATTTTCTCTCTTCAGCTCT--------CTTTTGCTCTTGCTGCTGATAGCGATGG

L. major    1217 ACAAAACGTTATTGCCGGGAAACCTTTC-CTTTCTGCGTTGTTTTAATATATATATATAT
L. tropica  1233 ATTAAACGTCATTGCTGCAAAATCTTTTTCTTTCTGTGTTGTTCCAAGATGATTATATAT
```

```
L. donovani 1227 ACGAAAAGTTGTTGCTGCAAAATCTTTC-CTTTCTGCGTTGCTTCAAGATGATCACATAT
L. infantum 1226 ACGAAAAGTTGTTGCTGCAAAATCTTTC-CTTTCTGCGTTGCTTCAAGATGATCACATAT

L. major    1276 ATGTCTGTATCTTTTTTGTGTCTCACCCCTCTTCTTTCCACTCTTTCTCCTTGTTCCCC-
L. tropica  1293 GACTCTT----------TTTTGTCTCACCCTTCTTTGCACCCTCTCTCCTTGCTAC-CC
L. donovani 1286 ATGACTC------ATTTTTGTTTCACATCTCTTCTTTCCACCTTTTCTCCTTGCTACCCT
L. infantum 1285 ATGACTC------ATTTTTGTTTCACATCTCTTCTTTCCACCTTTTCTCCTTGCTACCCT

L. major    1335 ------CTTTTTTCGTTACGGAAAGCGAACTGTCTAACGCTTTGCATTTCTTTGGTTGAA
L. tropica  1342 CTTTTTTTCTTTACTGAAAGCGAACTTTTGTTTCTAACGCTTTGCATTTCTTCGGTTGAC
L. donovani 1340 TTCTTTCTCTTTACTGAAAGCGAACCTTTTTTTCTACCTCTTAGCATTTCTTCGGTTGAA
L. infantum 1339 TTCTTTCTCTTTACTGAAAGCGAACCTTTTTTTCTACCTCTTAGCATTTCTTCGGTTGAA

L. major    1389 TGCCAGATTTCACGCGAAGCTGAGAAGGTGAACCTAGTTATCAGCAGCGTACTCTTCCTA
L. tropica  1402 TGCCGGATTTCAAGAGAAGCTGAGAAGGTGAACCGTGTTGCCAGCAGTGTACTCTTCCCA
L. donovani 1400 TGCCCGAGTTCACGAAAAGCTGTGAAGGTGAACCGTGTTATCAGCAGCGTATTCTTCCCA
L. infantum 1399 TGCCCGAGTTCACGAAAAGCTGTGAAGGTGAACCGTGTTATCAGCAGCGTATTCTTCCCA

L. major    1449 CTACCTCTATCACTTTCTTCTAACACTTCCACACAAACACACA------------CACAC
L. tropica  1462 CTACCTCTATCACTTTCTTGTAACACTTCCACACAGACACAGA--------------CAC
L. donovani 1460 CTACCTCTATCACTTTCTTCTAACACTTCCGCACACACA--------------CACACAC
L. infantum 1459 CTACCTCTATCACTTTCTTCTAACACTTCCGCACAGACACACACAGACACACACACACAC

L. major    1497 ACACACACACCATGTTGCTGTGCGAATGCGAACCTGAGTGGGTGCAAAGTGTGATTCCCA
L. tropica  1508 ACACACACACCATGTCGCTGTGCGAATGCGAACCTGAGTGGCTGCAAAGCGTGATTCCCA
L. donovani 1506 ATAAACACACAATTTCGGTGTGCGAATGCAAACCTGAGCGAGTGCAAAGCGTGATTCCCA
L. infantum 1519 ATAAACACACAATTTCGGTGTGCGAATGCAAACCTGAGCGAGTGCAAAGCGTGATTCCCA

L. major    1557 TAACGATGAGCGTCACTTCACACTTACATTTTCTTGTTTTCTTTTCCTTTTGTTTTGTCA
L. tropica  1568 TAACGATGAGCGTCACGGCACGTTTACATTTTCTTGATTGCTTTCCTTTCCTTTTCAC--
L. donovani 1566 TAACTATGAGCGTCACTGCGCATTTACATTTTATTGTTTGCTTTTCCTTTTTTTTAAC--
L. infantum 1579 TAACTATGAGCGTCACTGCGCATTTACATTTTATTGTTTGCTTTTCCTTTTTTTAAAC--

L. major    1617 CTTTCCGGCATACCATGGGAAAAAGGTGAGTGCACACGGGGCGCTTCTTGCGTGAGGCTT
L. tropica  1626 -TCTCCG-GCATATCGTGCGAAAAGATGAGTGCACACGGCGTACTTCTTGCGTGAGGCTT
L. donovani 1624 -TCTGCG-GCATGTCATGCGAAAAGATGAGTGCACATGGCGTGCTTCCTGCGTGAGGCTT
L. infantum 1637 -TCTGCG-GCATGTCATGCGAAAAGATGAGTGCACATGGCGTGCTTCCTGCGTGAGGCTT

L. major    1677 GGCTTGCCTTGCACGCACTAGGTGACCCAGCGTGACGGCAGGCAATGGATGCTGATGCTG
L. tropica  1684 GGCTTGCCTTGCACGCGCTAGGTGACCCAGCGTGGCGGTAGGACATAGACGCCGATGCTG
L. donovani 1682 GGCTTGCCTTGCACGCGCAAGGTGACCCAGCGTCGCGGTAGGAAATGGATGCTGATGTCG
L. infantum 1695 GGCTTGCCTCGCACGCGCAAGGTGACCCAGCGTCGCGGTAGGAAATGGATGCTGATGCCG

L. major    1737 TTTGAAAAAGAGGCAGGTGAAGTGCTATAAGTTTTCGTCGCAAGGAACTTTCTCGTGGCA
L. tropica  1744 TCTGAAAAAGAAGCAGGTGAAGTTCTATAGGGTTTCGTCGCTAAGGGCTTTCCAGTGGC-
L. donovani 1742 TCTGAAAAAGAAGCAGGCGAAGTGCTATAGGGTTTCGTCGCTAAAAGCCTTCCGGTGGCA
L. infantum 1755 TCTGAAAAAGAAGCAGGCGAAGTGCTATAGGGTTTCGTCGCTAAAAGCCTTCCGGTGGCA

L. major    1797 AACGAACGGCAGCAAAAAAGGGAG
L. tropica       ------------------------
L. donovani 1802 AACAAACCGCAAAAAAAAGAGAG-
L. infantum 1815 AACAAACCGCAAAAAAAAGAGAG-
```
