# Supplementary material for: Species-Specific Antimonial Sensitivity in Leishmania Is Driven by Post-Transcriptional Regulation of AQP1
Source: PLoS Negl Trop Dis. 2015 Feb 25;9(2):e0003500. doi: 10.1371/journal.pntd.0003500 (PMC4340957; doi:10.1371/journal.pntd.0003500)
Supplement: S1 Table — B. Copy numbers of AQP1-3’-UTR constructs. (PDF) [file pntd.0003500.s013.pdf]

Table S1A: Copy numbers of *LUC* in presence of species-specific 3' UTRs of AQP1 mRNA at the 3' end

Host species	LUC copy number						
	pLUC	pLUC-Ld	pLUC-Li	pLUC-Lm	pLUC-Lt	pLUC-Lb	pLUC-Lp
L. donovani	1.0	0.9651	1.1167	0.97	1.01	1.0242	1.0141
L. infantum	1.0	1.013	1.026	1.0043	0.9872	0.9437	0.9672
L. major	1.0	0.9438	0.9548	1.0658	1.027	1.2319	1.0026
L. tropica	1.0	0.9409	1.0025	0.9873	1.0069	0.9502	0.9324
L. braziliensis	1.0	1.0053	0.9889	0.9842	1.0042	0.9736	1.0021
L. panamensis	1.0	1.032	1.0211	0.9857	1.0027	0.9847	1.0022

Table S1B: Copy numbers of AQP1-3'-UTR constructs

Host Species	Copy number of AQP1-3'-UTR				
	Vector alone	pLd-Ld	pLd-Lm	pLm-Ld	pLm-Lm
L. donovani	1.0	1.0015	0.9671	1.0120	1.0205
L. major	1.0	0.9725	1.0101	1.0047	0.9953
