# Supplementary material for: Species-Specific Antimonial Sensitivity in Leishmania Is Driven by Post-Transcriptional Regulation of AQP1
Source: PLoS Negl Trop Dis. 2015 Feb 25;9(2):e0003500. doi: 10.1371/journal.pntd.0003500 (PMC4340957; doi:10.1371/journal.pntd.0003500)
Supplement: S2 Table — (PDF) [file pntd.0003500.s014.pdf]

Table S2: List of primers used in this study

Procedure	Species	Primer sequence	Restriction site (underlined)	Target	Amplicon size (bp)
**Mapping of AQP1 3'UTR**	*L. donovani, L. infantum* and *L. major*	Sense: 5'- CGCCACATACCCTAATGTTGC -3'<br>Antisense: 5'-CUACUACUACUAGGCCACGCGTCGACTAGTAC-3'	Not Applicable	AQP1 3'UTR	Not Applicable
	L. tropica	Sense: 5'- GATTTGTCGCCTCGCATACTCTCG -3'<br>Antisense: 5'-CUACUACUACUAGGCCACGCGTCGACTAGTAC-3'			
	L. braziliensis and *L. panamensis*	Sense: 5'- CCCGTATATTCACAGCCATGCTCTG -3'<br>Antisense: 5'-CUACUACUACUAGGCCACGCGTCGACTAGTAC-3'			
**Cloning of AQP1 3'UTR + 200 downstream of PolyA site into LUC vector**	*L. donovani* and *L. infantum*	Sense: 5'- GGATCCACGTGCTTCGCTACCCCTG -3'<br>Antisense: 5'-GGATCCGCCATTTTTTATACCGTGG-3'	BamH1	AQP1 3'UTR	~ 2000
	L. major	Sense: 5'- GGATCCGCGTGCTTCGCTACCCCTG -3'<br>Antisense: 5'-GGATCCCGTTTTTCATACCGTGG-3'	BamH1		
	L. tropica	Sense: 5'- GGATCCGCGTGCTTCGCTACCCCTG -3'<br>Antisense: 5'-GGATCCTCCTACCGTGGACCCGATGC-3'	BamH1		
	L. braziliensis	Sense: 5'- GTCGAC AGCTGCACTGCTGCTC -3'<br>Antisense: 5'-GTCGACCAGAGAGGACTTCCATTGTTATTAC-3'	SalI		
	L. panamensis	Sense: 5'- GTCGAC AGATGCACTGCTGTGC -3'<br>Antisense: 5'-GTCGACCAGAGAGGACTTCCATTGTTATTAC-3'	SalI		
**Cloning of AQP1 ORF and 3'UTR + 200 downstream of PolyA site into pSP72-YHYG-αtubIR vector**	*L. donovani*	Sense: 5'- GGATCCATGCACGAGGAAGAGCAGG -3'<br>Antisense: 5'- TCTAGACTAGAAGTTGGGTGGAATG -3'	BamH1<br>XbaI	AQP1 ORF	846
	L. major	Sense: 5'- GGATCCATGCATGAGGAAGAGGAGGACC -3'<br>Antisense: 5'- TCTAGACTAACAGCTGGGCGGAATGAT -3'	BamH1<br>XbaI	AQP1 ORF	846
	L. donovani	Sense: 5'- TCTAGAACGTGCTTCGCTACCCCTG -3'<br>Antisense: 5'- TCTAGAGCCATTTTTTATACCGTGG -3'	XbaI	AQP1 3'UTR	~ 2000
	L. major	Sense: 5'- TCTAGAGCGTGCTTCGGTACC -3'<br>Antisense: 5'- TCTAGACGTTTTTCATACCGTGG -3'	XbaI	AQP1 3'UTR	~ 2000
**Mapping of LUC 3'UTR**	All 6 species of this study	Sense: 5'- CAACACCCCAACATCTTCGACGC -3'<br>Antisense: 5'-CUACUACUACUAGGCCACGCGTCGACTAGTAC-3'	Not Applicable	LUC 3'UTR	Not Applicable
**QPCR**	*L. donovani and L. infantum*	Sense: 5'- CTGTGTCTTTGGTGCCTTTCC -3'<br>Antisense: 5'- GCCTTTTGGGCGTCGTC -3'	Not Applicable	AQP1	129
	L. major and *L. tropica*	Sense: 5'- CAGTCCAACTCAAGCTACCTG -3'<br>Antisense: 5'- AGGTACCAAAGACACAGTTCG -3'			136
	L. braziliensis and *L. panamensis*	Sense: 5'- GCGGTGTGGAGTGAGATATTC -3'<br>Antisense: 5'- GAGTTGATACCTGTCGTGATACC -3'			149
	All 6 species of this study	Sense: 5'- GTGTTGGGCGCGTTATTTATC -3'<br>Antisense: 5'- TAGGCTGCGAAATGTTCATACT -3'	Not Applicable	LUC	97
	L. donovani and *L. infantum*	Sense: 5'- CGTGGAGCGTGTGATGTATT -3'<br>Antisense: 5'- CTGTGCCACTAACTCCTTCATC -3'	Not Applicable	MRPA	76

**QPCR**	*L. major*	Sense: 5'- GCGCAGTACGACAAGATCA -3'<br>Antisense: 5'- CTCCACCATGCTGTGGAATA -3'	Not Applicable	MRPA	108
	L. tropica	Sense: 5'- CTGACGGAAACGCTCAACT -3'<br>Antisense: 5'- TCCACTTCGTGGGTGTAGTA -3'			98
	L. braziliensis and *L. panamensis*	Sense: 5'- GATGGACCACGGTGTTGTT -3'<br>Antisense: 5'- GGAGAAGGTGGTGGAATTCTTT -3'			
	L. donovani, L. infantum and *L. tropica*	Sense: 5'- GGAGATAGACAAGGCCATCAAG -3'<br>Antisense: 5'- TGTCGTTGATGAAGTCAGAGC -3'	Not Applicable	GAPDH	101
	L. major	Sense: 5'- GAGATCGACAAGGCCATCAA -3'<br>Antisense: 5'- AGCTGCGGTTGTCGTTAATA -3'			109
	L. braziliensis and *L. panamensis*	Sense: 5'- GGTGAATGGCTACCACATCAA -3'<br>Antisense: 5'- GCCAGTCGACTCAATCACATAG -3'			100
	L. donovani and *L. infantum*	Sense: 5'- AGGTGAGCGACGTTGTTATC -3'<br>Antisense: 5'- GTAGCCGCCATCCACTTT -3'	Not Applicable	PTR1	83
	L. major	Sense: 5'- GTGAACAACGCCTCTTCTTTC -3'<br>Antisense: 5'- CGTCTCCATTGCCTCTCTATC -3'			96
	L. tropica	Sense: 5'- AGGTGAGCGACGTTGTTATC -3'<br>Antisense: 5'- CTGTAGCCACCATCCACTTT -3'			85
	L. braziliensis	Sense: 5'- GCTCCTATTTCCCTCGTTCAG -3'<br>Antisense: 5'- GTAGGCAGAGGCGTTGTTTA -3'			99
	L. panamensis	Sense: 5'- CACCCATTTCCCTCGTTCA -3'<br>Antisense: 5'- GTAGGCAGAGGCGTTGTTTA -3'			98
	L. donovani and L. major	Sense: 5'- CGACGTCTGTCGAGAAGTTT -3'<br>Antisense: 5'- TTGTAGAAACCATCGGCG -3'	Not Applicable	*Hygromycin phosphotransferase*	154
