# Supplementary material for: Species-Specific Antimonial Sensitivity in Leishmania Is Driven by Post-Transcriptional Regulation of AQP1
Source: PLoS Negl Trop Dis. 2015 Feb 25;9(2):e0003500. doi: 10.1371/journal.pntd.0003500 (PMC4340957; doi:10.1371/journal.pntd.0003500)
Supplement: S3 Table — (PDF) [file pntd.0003500.s015.pdf]

Table S3: Relative (to *L. donovani*) expression of GAPDH mRNA with respect to β-tubulin mRNA in different species of *Leishmania*.

Host Species	β-tubulin
L. donovani	1.0
L. infantum	0.989
L. major	1.004
L. tropica	1.021
L. braziliensis	1.001
L. panamensis	1.004
